# Gene expression changes implicate specific peripheral immune responses to Deep and Lobar Intracerebral Hemorrhages in humans

**DOI:** 10.1016/j.hest.2022.04.003

**Published:** 2022-04-22

**Authors:** Bodie Knepp, Bradley P. Ander, Glen C. Jickling, Heather Hull, Alan H. Yee, Kwan Ng, Fernando Rodriguez, Paulina Carmona-Mora, Hajar Amini, Xinhua Zhan, Marisa Hakoupian, Noor Alomar, Frank R. Sharp, Boryana Stamova

**Affiliations:** a Department of Neurology, School of Medicine, University of California at Davis, Sacramento, CA, USA; b Department of Medicine, Division of Neurology, University of Alberta, Edmonton, Canada

**Keywords:** RNA expression, Blood, Intracerebral hemorrhage, Deep hemorrhage, Lobar hemorrhage, Amyloid, T Cells, Neutrophils

## Abstract

The peripheral immune system response to Intracerebral Hemorrhage (ICH) may differ with ICH in different brain locations. Thus, we investigated peripheral blood mRNA expression of Deep ICH, Lobar ICH, and vascular risk factor-matched control subjects (n = 59). Deep ICH subjects usually had hypertension. Some Lobar ICH subjects had cerebral amyloid angiopathy (CAA). Genes and gene networks in Deep ICH and Lobar ICH were compared to controls. We found 774 differentially expressed genes (DEGs) and 2 co-expressed gene modules associated with Deep ICH, and 441 DEGs and 5 modules associated with Lobar ICH. Pathway enrichment showed some common immune/inflammatory responses between locations including Autophagy, T Cell Receptor, Inflammasome, and Neuroinflammation Signaling. Th2, Interferon, GP6, and BEX2 Signaling were unique to Deep ICH. Necroptosis Signaling, Protein Ubiquitination, Amyloid Processing, and various RNA Processing terms were unique to Lobar ICH. Finding amyloid processing pathways in blood of Lobar ICH patients suggests peripheral immune cells may participate in processes leading to perivascular/vascular amyloid in CAA vessels and/or are involved in its removal. This study identifies distinct peripheral blood transcriptome architectures in Deep and Lobar ICH, emphasizes the need for considering location in ICH studies/clinical trials, and presents potential location-specific treatment targets.

## 1. Introduction

Intracerebral hemorrhage (ICH) makes up 10–15% of all strokes.^1-6^ ICH can occur in Lobar (cortical) or Deep intraparenchymal brain regions^7^ with one year mortality rates of 57% and 51% for those locations, respectively.^6,8^ Lobar ICH tends to have higher hemorrhage volumes than Deep ICH.^9^ Deep ICH tends to be associated with hypertension, while Lobar ICH tends to be associated with cerebral amyloid angiopathy (CAA), though other factors can contribute in both locations.^3,4,6^ High blood pressure affects arterial blood vessel walls in the brain, increasing the potential for a rupture leading to Deep ICH.^10^ CAA is the result of Amyloid Beta (Aβ) deposition in and around blood vessels in the brain, leading to decreased vascular integrity and propensity for Lobar microbleeds and Lobar ICH.^11^ Because of the different risk factors, it has been suggested that the different hemorrhage locations have different pathophysiologies.^12,13^ However, relatively little is known about the molecular underpinnings of any such differences. Since the peripheral immune system responds to ICH,^14-16^ we examined the human peripheral whole blood transcriptome to find similarities and differences between Deep and Lobar ICH responses at gene-level and gene co-expression network level. We found common enrichment in many immune, inflammatory, and cell death pathways between locations, as well as some responses unique to Deep ICH and Lobar ICH. These unique responses may help elucidate different molecular mechanisms of damage and repair in the two ICH locations, and the associated genes and pathways may guide the search for novel location-specific therapeutic targets.

## 2. Methods

Detailed methods can be found in the Supplemental Manuscript.

### 2.1. Subjects, arrays, and data processing

We analyzed 59 subjects: 9 with Lobar ICH, 19 with Deep ICH, and 31 vascular risk factor matched controls (VRFC, C) (Table 1). Subjects with Deep ICH had hemorrhages in the basal ganglia, thalamus, cerebellum, and pons/brainstem detected by CT or MRI scans. Subjects with Lobar ICH had hemorrhages anywhere in the cortex and could extend into adjacent white matter detected by CT or MRI scans. Cerebral Amyloid Angiopathy (CAA) was diagnosed as probable or possible with appropriate MRI sequences according to modified Boston Criteria.^17^ Peripheral whole blood was collected from each subject via venipuncture in PAXgene tubes at a single time-point (within 4.2 and 101.3 hours, average 50.2 hours, post *ictus* in Deep ICH subjects; and within 37.7 and 124.3 hours, average 71.5 hours, post *ictus* in Lobar ICH subjects) (Table 1). Isolated RNA was processed and hybridized on Gene-Chip^®^ Human Transcriptome Arrays (HTA) 2.0 (Affymetrix, Santa Clara, CA) to examine the coding (mRNA) and some of the noncoding human transcriptome.

### 2.2. Differential expression analysis

Differential expression (DE) analysis was conducted at the gene level. The ANCOVA (Analysis of Covariance) model included Age, Group (Deep ICH, Lobar ICH, or VRFC), Sex, and Group*Sex interaction. Significant DE for each ICH Location comparison was defined as the overlap of Group-significant genes (Benjamini Hochberg False Discovery Rate (BH) multiple test corrected p < 0.05) and contrast-significant genes (p < 0.005; Fold-Change > ∣1.2∣) for the contrasts Deep ICH vs. VRFC, Lobar ICH vs. VRFC, and Deep ICH vs. Lobar ICH.

We also investigated sex differences. Due to a limited number of female subjects, the sex-specific results are pilot in nature and need to be reproduced in larger sample sizes. Sex-specific gene lists were selected using modified criteria due to the smaller sample size: genes passing p < 0.005 and Fold-Change > ∣1.2∣ for a contrast were considered significant (contrasts: Deep ICH Males vs. VRFC Males; Deep ICH Females vs. VRFC Females; Lobar ICH Males vs. VRFC Males; Lobar ICH Females vs. VRFC Females). Identification of sex-specific genes associated with ICH in each location was done by overlapping corresponding male- and female- lists.

### 2.3. Weighted gene co-expression network construction and analysis

Two co-expression networks were generated: Deep ICH + VRFC subjects (DeepICHandVRFC) and Lobar ICH + VRFC subjects (LobarICHandVRFC). Weighted Gene Co-Expression Network Analysis (WGCNA) was run in R to generate networks of modules (groups of co-expressed genes).^18,19^ Hub genes were defined as the most interconnected genes in each module and represent potential master regulators.^20,21^ Module significance for Group and other technical and clinical variables (including age, sex, and vascular risk factors) was assessed using a *t*-test or a Pearson correlation to the module’s eigengene (first principal component of expression) for categorical and continuous clinical parameters, respectively (p < 0.05).^18^ Cytoscape was used to visualize significant networks.^22,23^

### 2.4. Biological enrichment

Enrichment in blood cell type-specific genes was identified using hypergeometric probability in R (*phyper*) by overlapping our per-gene lists and location-associated modules with lists of blood cell type-specific genes (p < 0.05).^24,25^ Ingenuity Pathway Analysis (IPA^®^, QIAGEN) was performed on all gene lists as previously described^26^ to identify significant Canonical Pathways, Disease and Function terms, and Upstream Regulators (BH p < 0.05). IPA predicts activation (Z ≥ 2) or inhibition (Z ≤ − 2) states of its results based on up- or down-regulation in our gene lists and IPA’s knowledge-base from the literature.^27,28^ DAVID Functional Annotation Bioinformatics Resources Database was used for Gene Ontology (GO) term enrichment (BH p < 0.05).^29,30^

## 3. Results

### 3.1. Subject demographics

Subjects’ demographic and clinical characteristics are presented in Table 1. No statistically significant difference was found between Deep ICH, Lobar ICH, and VRFC groups for age, race, sex, diabetes, hypertension, smoking status, or hyperlipidemia. A total of 14/19 Deep ICH had hypertension, and a total of 4/9 Lobar ICH had possible or probable CAA. Deep ICH patients presented earlier following symptom onset (mean 50.2 hours) compared to those with Lobar ICH (mean 71.5 hours; p = 0.041). We examined the effect of time on the hierarchical clustering distribution of the differentially expressed genes between subjects with Deep and Lobar ICH and found they did not cluster by time and the main clustering was driven by the ICH location (SFigure 1). Additionally, Deep ICH subjects were younger (mean age 56.3 years) than Lobar ICH subjects (mean age 68.2 years; p = 0.026). Age was included in the ANCOVA.

### 3.2. Gene-level differential expression based on ICH location reveals common and specific transcriptional response

Expression of 995 genes were significant for Group (BH p < 0.05). One thousand three hundred and fifty-five genes were DE in Deep ICH vs. VRFC; 629 were DE in Lobar ICH vs. VRFC; 94 were DE in Deep ICH vs. Lobar ICH (p < 0.005, FC > ∣1.2∣) (SFigure 2).

#### 3.2.1. Genes differentially expressed in Deep ICH vs. VRFC – DeepPerGene list

**Fig. 1.**
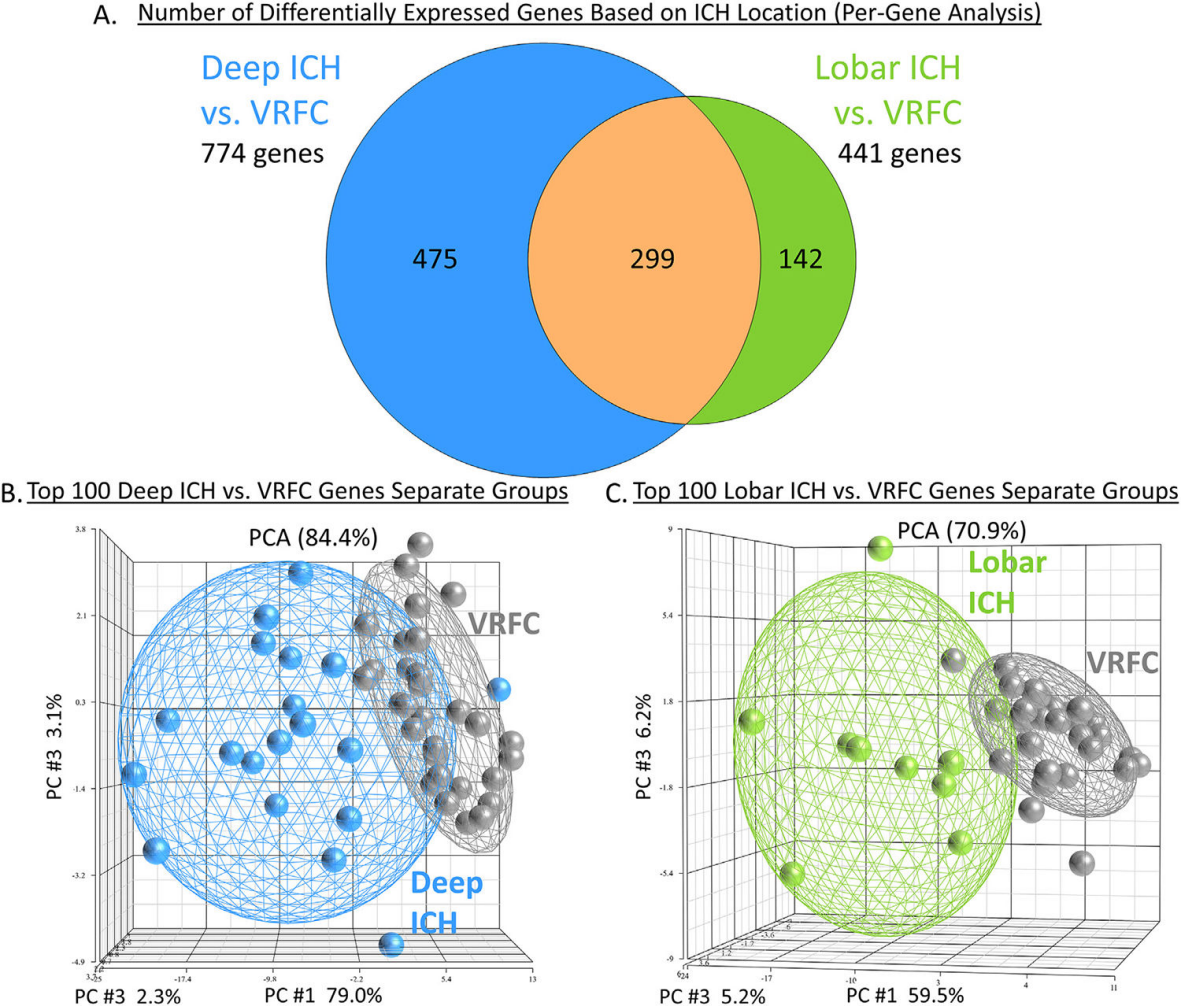
Venn Diagrams of differentially expressed gene lists from the per-gene analyses of Deep ICH vs. VRCF and Lobar ICH vs. VRFC (A). The genes from these lists pass p < 0.005 and FC > ∣1.2∣ for the specific contrast, as well as BH < 0.05 for Group. Deep ICH vs. VRFC is hereafter referred to in the text as DeepPerGene; Lobar ICH vs. VRFC is hereafter referred to in the text as LobarPerGene. Principal Component Analyses (PCA) of the top 100 most differentially expressed genes in DeepPerGene (B) and LobarPerGene (C). Ellipsoids in (B) and (C) represent 2 standard deviations from the centroid of each group. ICH – intracerebral hemorrhage; VRFC – vascular risk factor control.

**Fig. 2.**
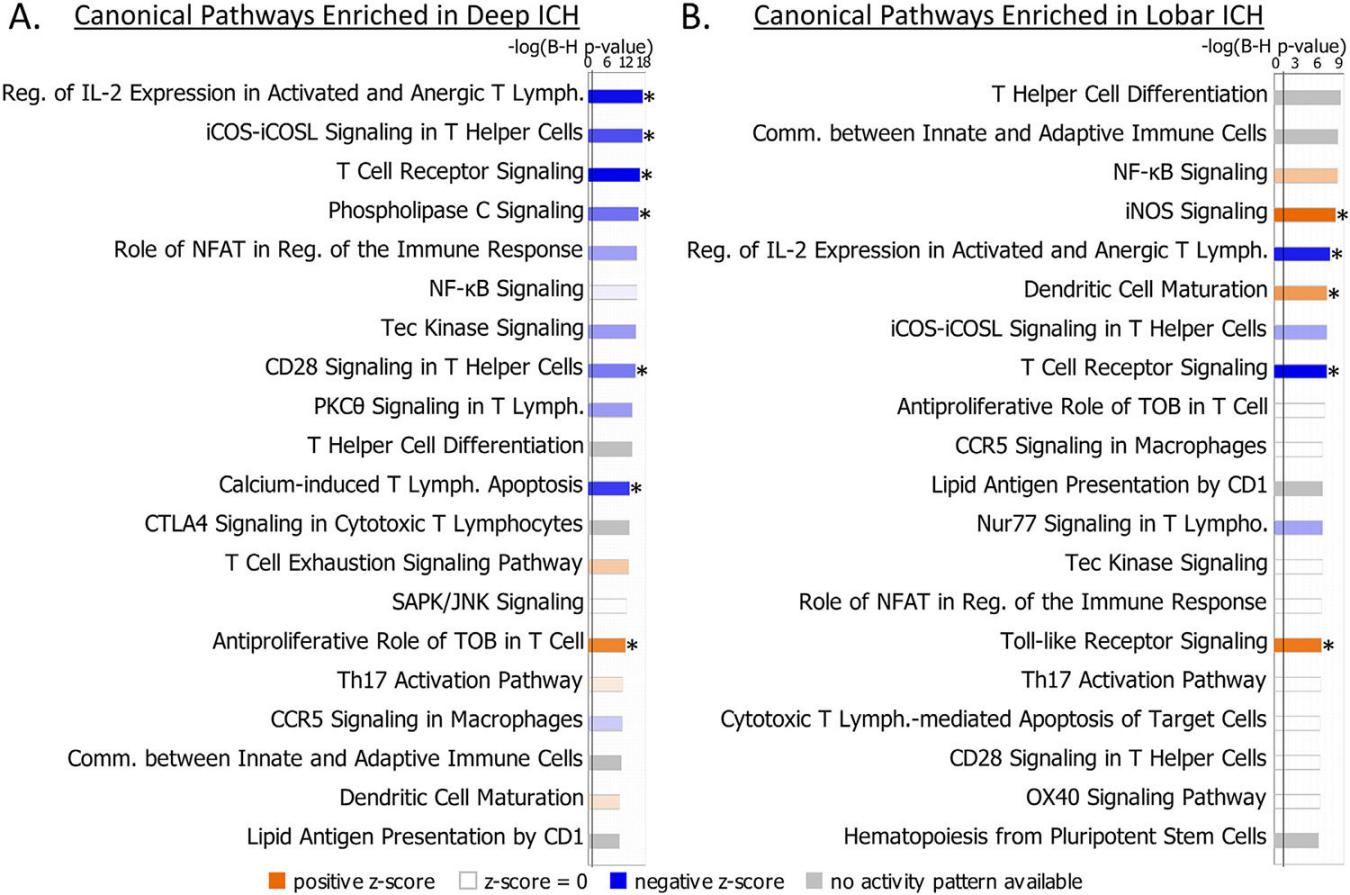
Pathway enrichment presented for DeepPerGene (Deep ICH vs. VRFC) (A) and LobarPerGene (Lobar ICH vs. VRFC) (B) gene lists. The top 20 relevant significant pathways are displayed. Bar shading represents activity pattern prediction (blue for suppression/negative Z-score and orange for activation/positive Z-score) where darker color represents larger absolute Z-score; * represents statistically significant activity pattern prediction (Z ≥ 2, significant activation in the ICH subgroup compared to VRFC; Z ≤ −2, significant suppression in the ICH subgroup compared to VRFC). X-axis: −log_10_ (BH p-value). Any pathway having −log_10_ (BH p value) > 1.3 (corresponding to BH p < 0.05; depicted by vertical black line) is significant. Lymph. – Lymphocyte; Reg. – Regulation; Comm. – Communication.

**Fig. 3.**
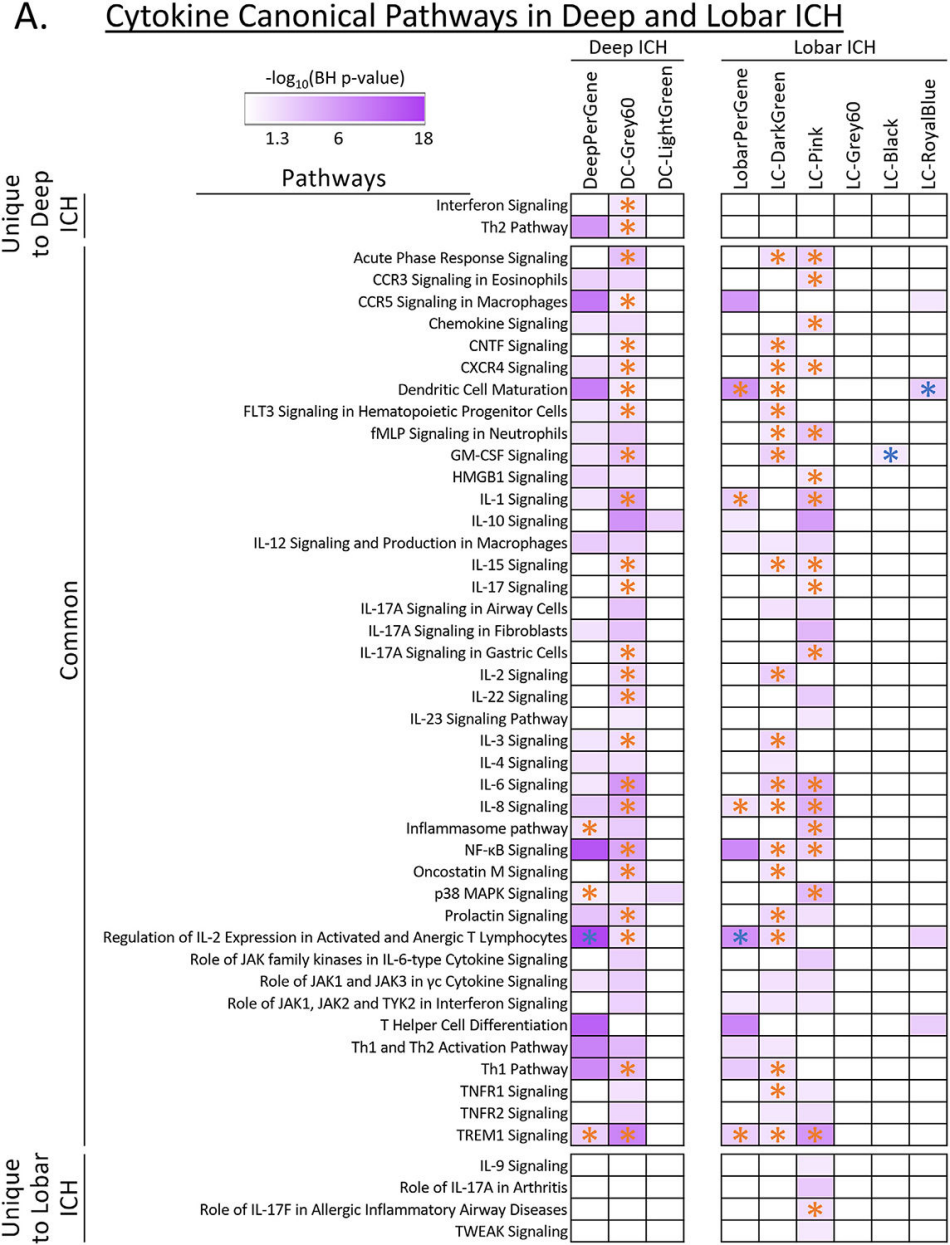

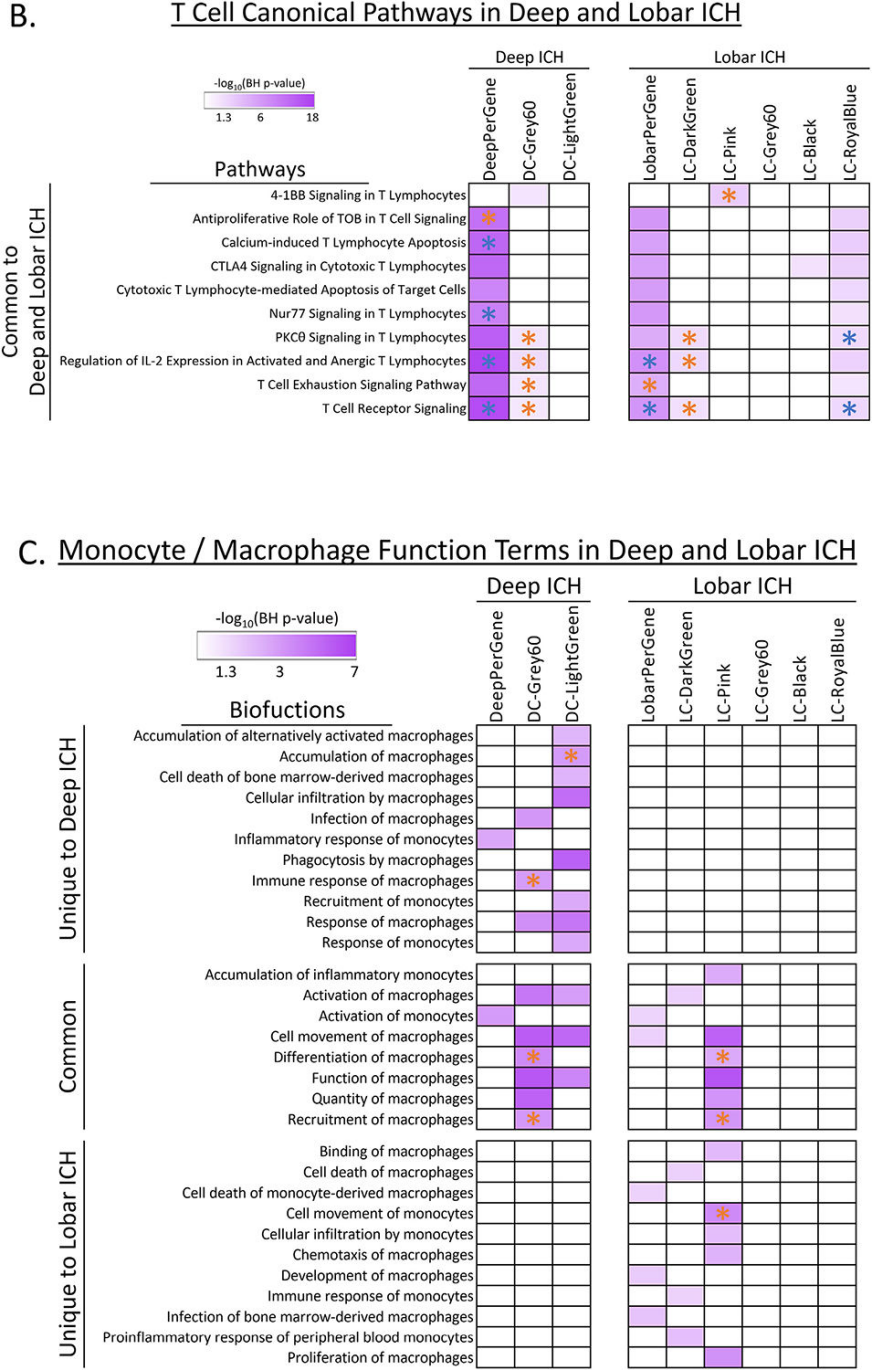

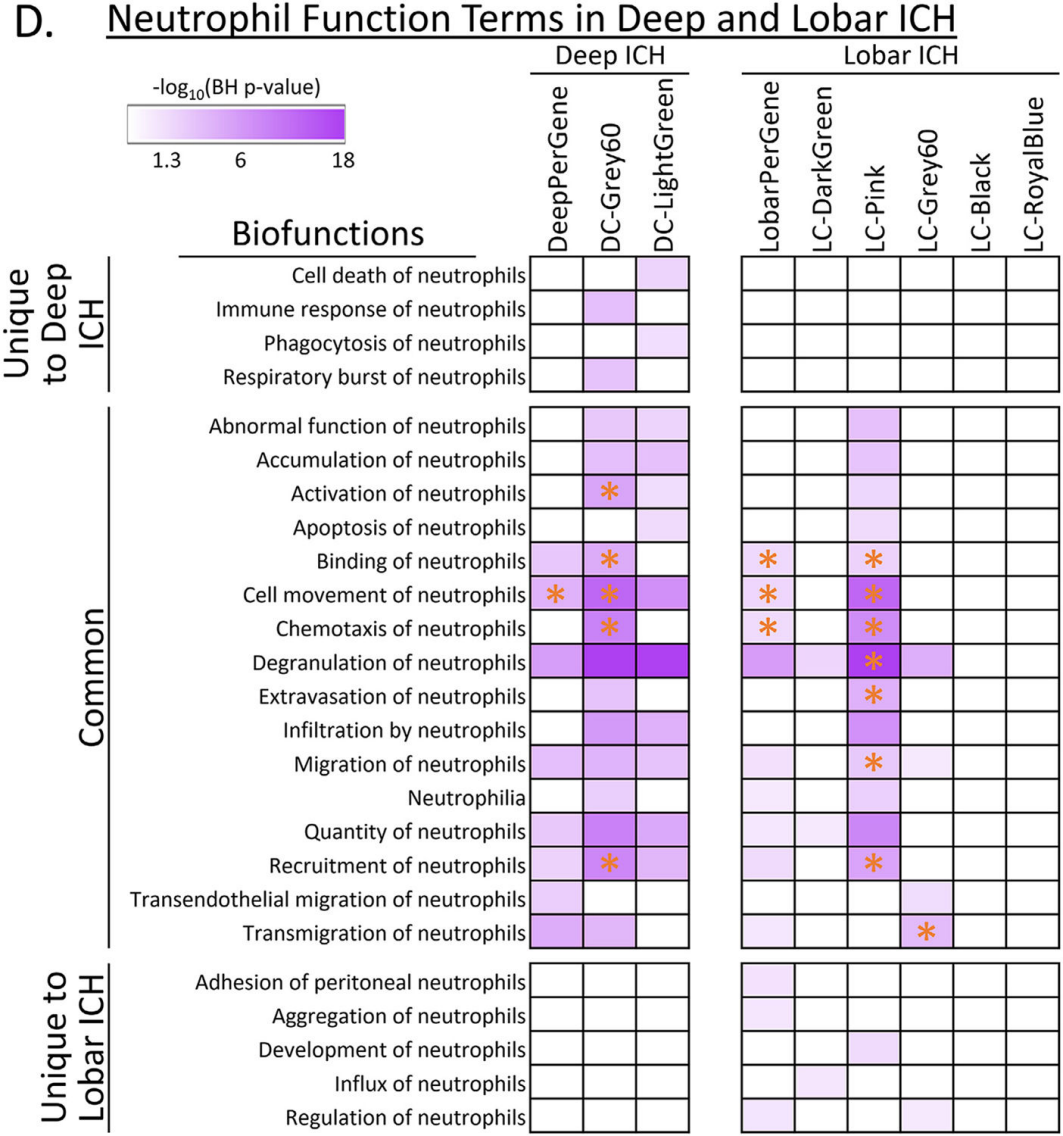
Heatmaps depicting relevant Cytokine Signaling (A) and T Cell (B) pathways and heatmaps depicting relevant Monocyte and Macrophage (C) and Neutrophil (D) biofunctions. Data presented for pathways/functions where at least one location-associated list was significant. Purple shading represents −log_10_(BH p value) where 1.3 corresponds to a BH p value of 0.05; higher −log_10_(BH p value) corresponds to lower (more significant) BH p value. Non-significant pathways/functions are displayed as white cells. * Function/pathway has a significant activity pattern prediction. If asterisk is orange, the pathway is activated in the ICH subgroup vs. VRFC; if asterisk is blue, the pathway is suppressed in the ICH subgroup vs. VRFC.

**Fig. 4.**
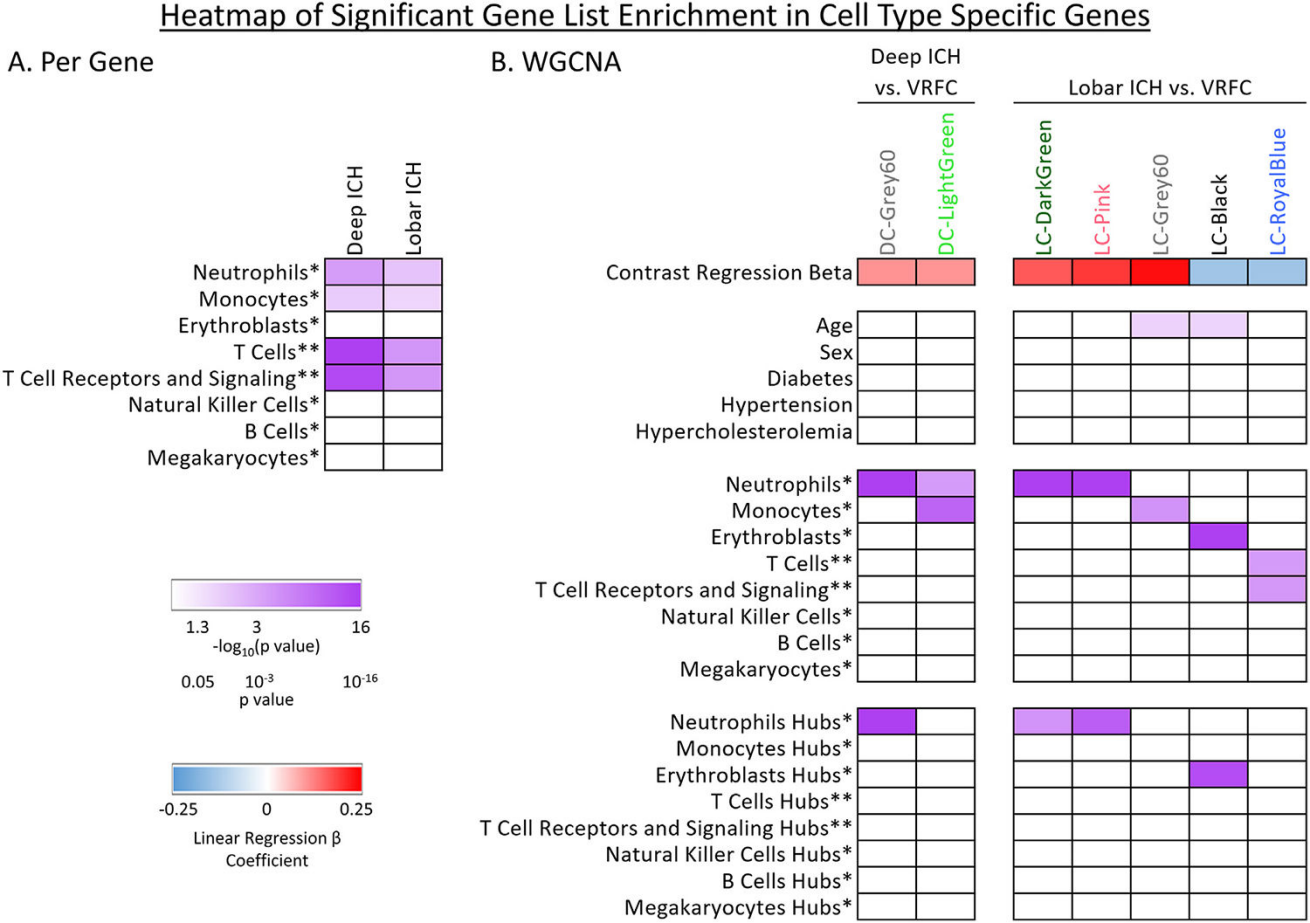
Enrichment in cell-type specific gene lists for the per-gene lists (A) and WGCNA modules (B). Purple shading represents −log_10_(p value) where 1.3 corresponds to a p value of 0.05; higher −log_10_(p value) corresponds to lower (more significant) p value. Non-significant hypergeometric probabilities are displayed as white cells. In panel (A), Deep ICH results are based on genes differentially expressed in Deep ICH vs. VRFC, and Lobar ICH – genes differentially expressed in Lobar ICH vs. VRFC. In panel (B), blue and red shading represents the beta coefficient for Group in a linear regression on the module eigengene (red represents genes upregulated in ICH; blue - downregulated in ICH). Significance for clinical parameters is presented in rows under Contrast Regression Beta; enrichment of hub gene lists in cell-type specific lists presented at the bottom. *Cell-type specific list from Watkins et al.^24^; **Cell-type specific list from Chtanova et al.^25^ For more comprehensive coverage of T cell-specific genes, ST1 and ST2 from Chtanova et al. were used; no overlaps with Watkins et al. Th and Tc lists were found.

The intersection between the 995 Group-significant and the 1,355 Deep ICH vs. VRFC-significant genes was 774 genes (Deep ICH DEGs; hereafter called DeepPerGene) (Fig. 1A; SFigure 2A; STable 1A). The top 100 genes (ranked by BH) of DeepPerGene list differentiated most of the Deep ICH from VRFC subjects in Principal Component Analysis (PCA) (Fig. 1B) and unsupervised hierarchical clustering (HC) (SFigure 3). Functional annotation of the DeepPerGene list is presented in STables 2A, 3A, and 4A. It was enriched in 156 pathways (STable 2A), of which 9 were activated (including iNOS, Toll-Like Receptor (TLR), and Neuroinflammation Signaling) and 9 were suppressed in Deep ICH compared to controls (including several T-cell pathways). The top 20 relevant significant canonical pathways are presented in Fig. 2A. Significant cytokine and T cell canonical pathways are presented in Fig. 3A and 3B, and significant Monocyte and Neutrophil biofunctions in Fig. 3C and 3D. The DeepPerGene list was also enriched in Monocyte, Granulocyte (mainly Neutrophil), T Cell, T Cell Receptor and T Cell Receptor Signaling-specific gene lists (Fig. 4A, STable 5A).

#### 3.2.2. Genes differentially expressed in Lobar ICH vs. VRFC – LobarPerGene list

The intersection between the 995 Group-significant and the 629 Lobar ICH vs. VRFC-significant genes was 441 (Lobar ICH DEGs; hereafter called LobarPerGene) (Fig. 1A; SFigure 2B; STable 1B). The top 100 genes (ranked by BH) of LobarPerGene differentiated most of the Lobar ICH from VRFC subjects in PCA (Fig. 1C) and HC (SFigure 4). The LobarPerGene list was enriched in 59 pathways (STables 2B,3B,4B), of which 11 were activated (including iNOS, TLR, T Cell Exhaustion, and IL-1 Signaling) and 3 were suppressed (including T Cell Receptor Signaling and Antioxidant Action of Vitamin C) in Lobar ICH compared to controls (SFigure 5). The top 20 relevant significant canonical pathways are presented in Fig. 2B. Significant cytokine and T cell canonical pathways are presented in Fig. 3A and 3B. Like DeepPerGene, LobarPerGene was enriched in Monocyte, Neutrophil, T Cell, and T Cell Receptor Signaling specific gene lists (Fig. 4A, STable 5B) and Monocyte and Neutrophil biofunctions (Fig. 3C,3D; STable 3B).

#### 3.2.3. Genes differentially expressed in Deep ICH vs. Lobar ICH – DeepVsLobar list

**Fig. 5.**
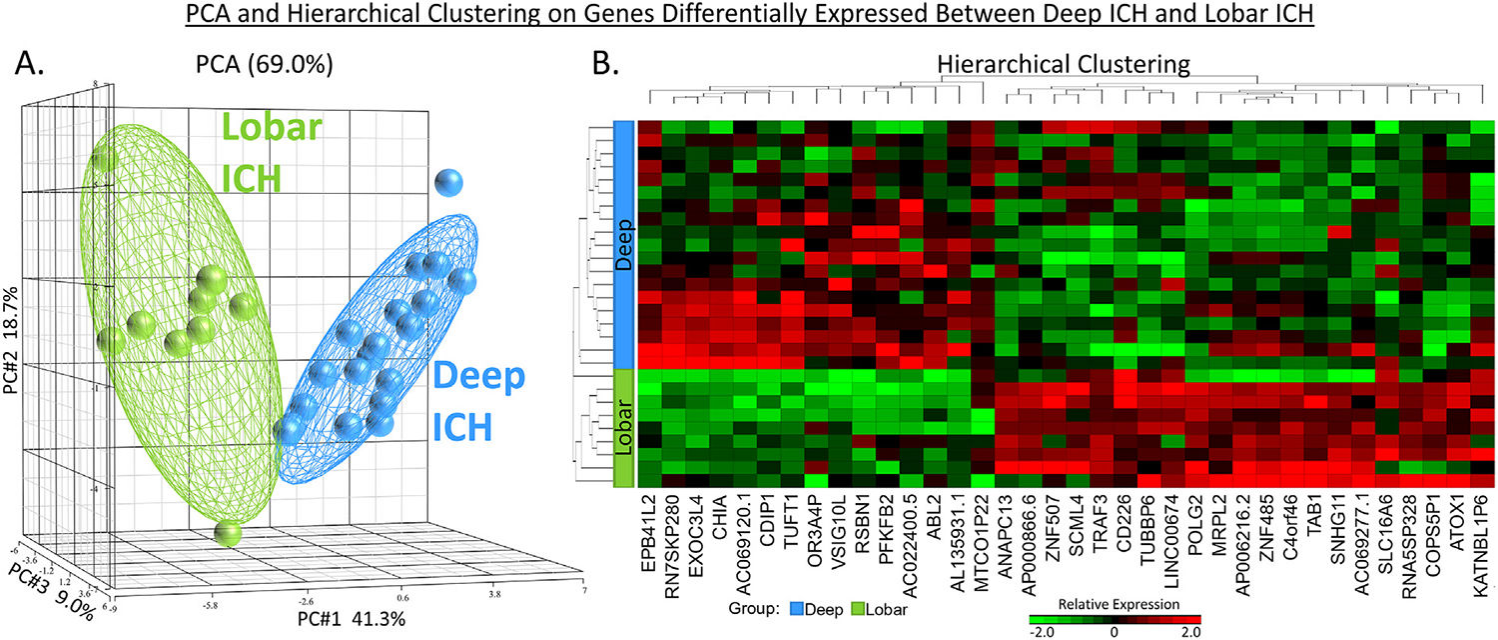
PCA (A) and Hierarchical Clustering (B) on the Deep ICH vs. Lobar ICH gene list (hereafter referred to as DeepVsLobar). These 36 genes differentiated Deep and Lobar ICH subjects. Ellipsoids in (A) represent 2 standard deviations from the centroid of each group.

The intersection between the Group significant and Deep ICH vs. Lobar ICH significant genes was 36 (hereafter called DeepVsLobar) (SFigure 2C; STable 1C). These 36 genes differentiated Deep ICH from Lobar ICH patients on PCA and HC (Fig. 5), providing additional evidence for transcriptome differences between Deep and Lobar ICH. The DeepVsLobar gene list was also able to separate most subjects in the 3 Groups (Deep ICH, Lobar ICH, and VRFC) on HC (SFigure 6). It was not significantly enriched in any biological pathways, GO terms, or cell type specific lists (STable 5C). However, it contained genes involved in immune, inflammatory, and other relevant processes (like Autophagy, IL-1, -6, -10, and -15 Signaling, and iNOS, TLR, NF-κB, TGF-β, WNT/β-catenin, and Neuroinflammation Signaling) (SFigure 7; STables 6ABC).

### 3.3. Weighted gene co-expression networks uncover specific transcriptome architecture in Deep and Lobar ICH

**Fig. 6.**
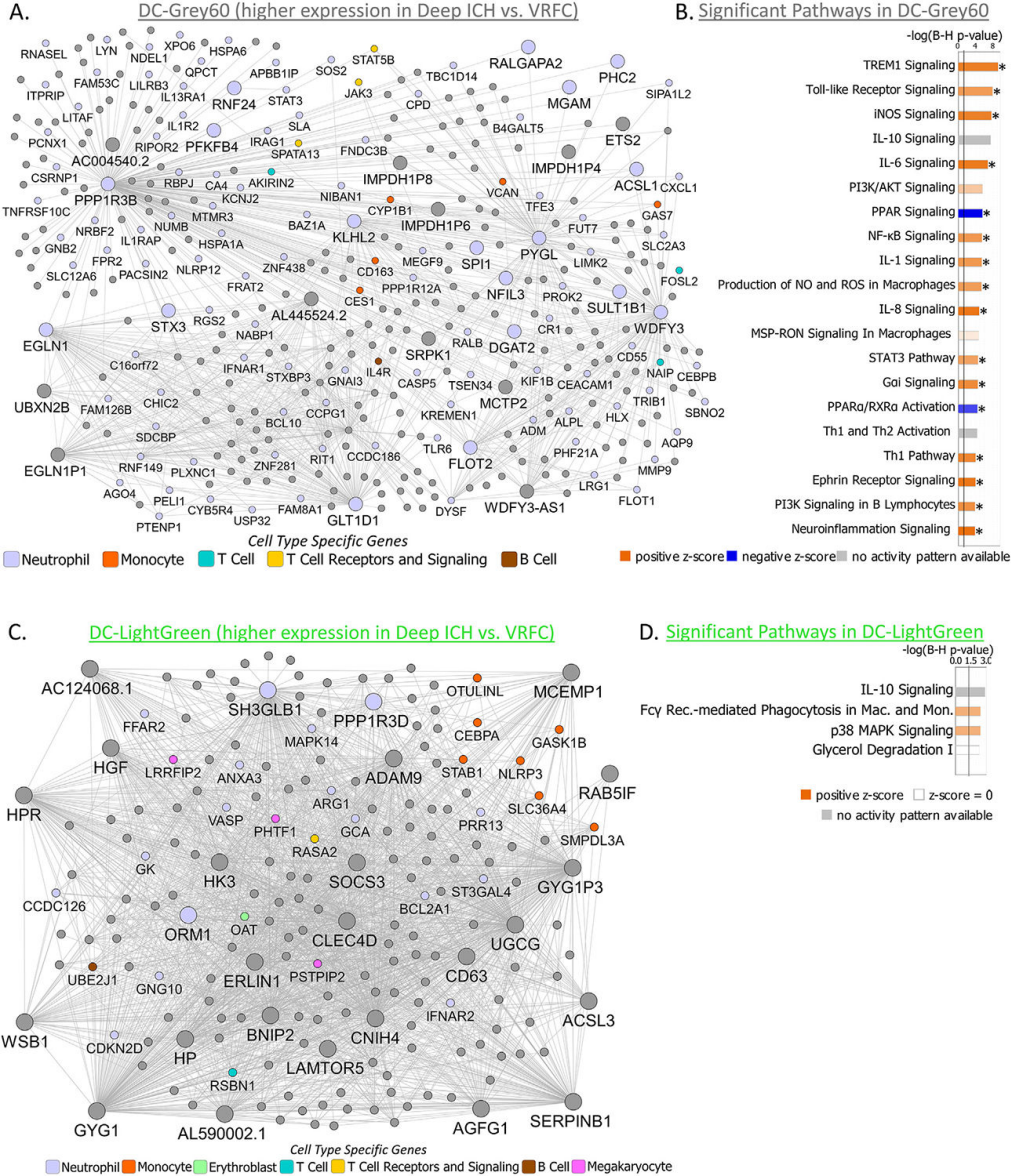
Network diagrams for the Deep ICH modules DC-Grey60 (A) and DC-LightGreen (C) show connectivity of hubs and genes within modules. Nodes represent genes within the module; edges represent connections based on co-expression between genes. Weaker connections and nodes with fewer connections have been filtered out to increase legibility. Larger nodes with large labels are hub genes, representing potential master regulators. Genes are grey by default and colored if they are cell type specific. Hubs, cell type specific genes, and other selected genes labeled. In panel (A), the genes *SOS2*, *SLA*, *APBB1IP*, and *STAT3* were members of both the Neutrophil specific and T Cell Receptors and Signaling specific gene lists and were colored as Neutrophil-specific. Pathway enrichment presented for DC-Grey60 (B) and DC-LightGreen (D). The top 20 relevant significant pathways are displayed. Significance threshold −log_10_(BH p value) of 1.3 (corresponds to BH p value of 0.05) depicted by a vertical black line. Higher −log_10_(BH p value) corresponds to lower (more significant) BH p value. Bar shading represents activity pattern prediction (blue for suppression/negative Z-score and orange for activation/positive Z-score) where darker color represents larger absolute Z-score; * represents statistically significant activity pattern prediction (Z ≥ 2, significant activation in Deep ICH compared to Controls; Z ≤ −2, significant suppression in Deep ICH compared to Controls). NO – Nitric Oxide; ROS – Reactive Oxygen Species; Rec. – Receptor; Mac. – Macrophages; Mon. – Monocytes.

**Fig. 7.**
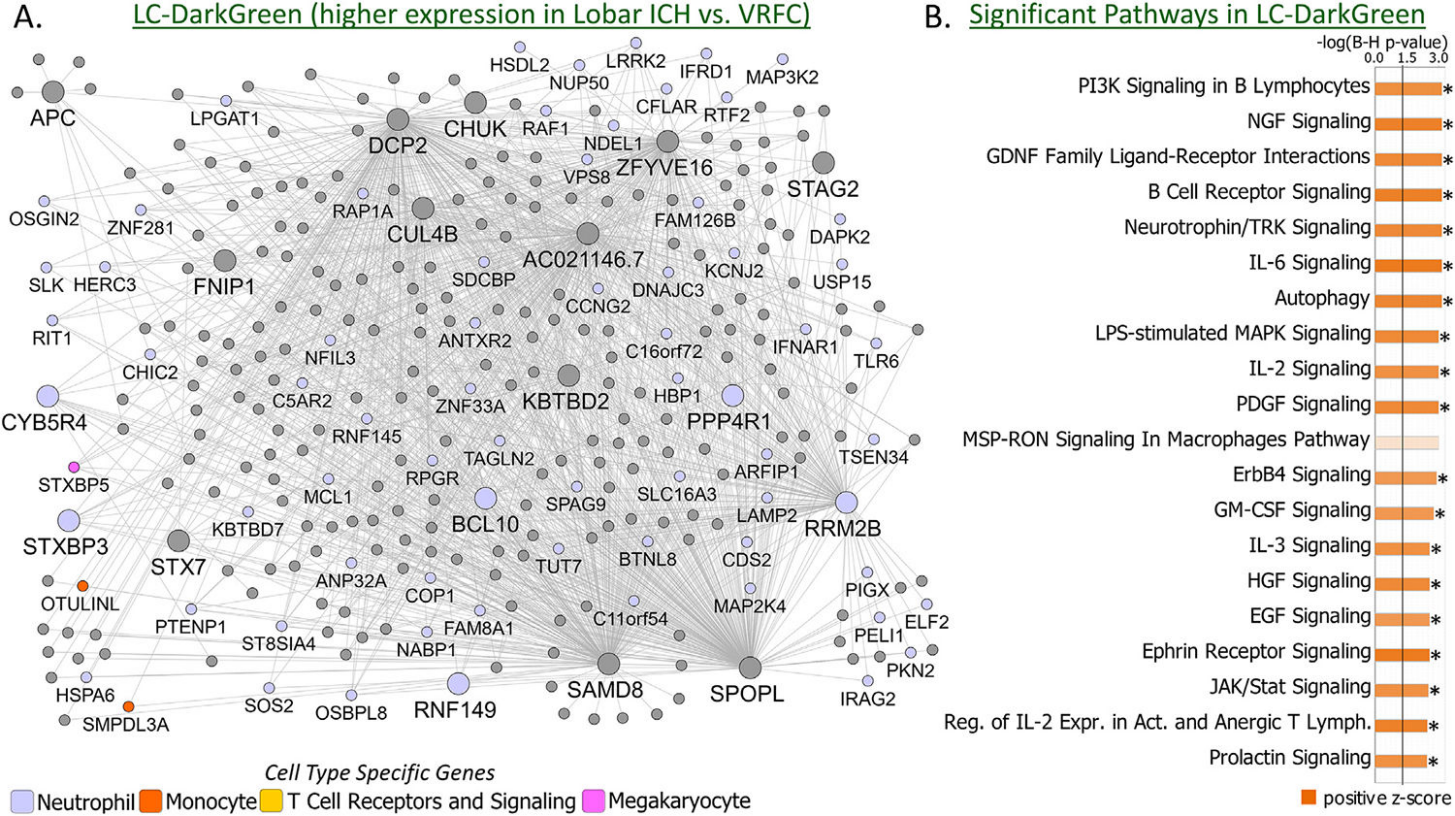

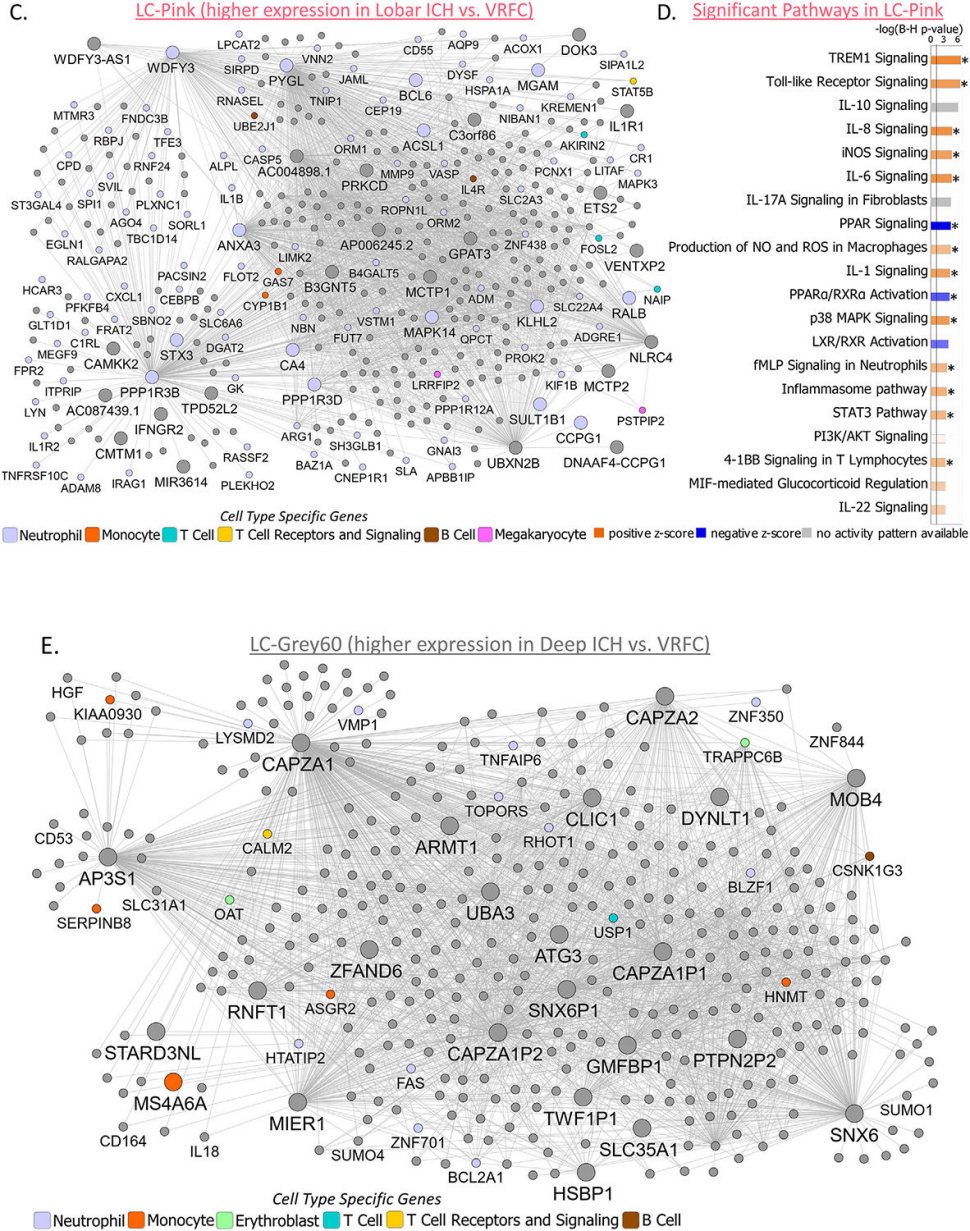

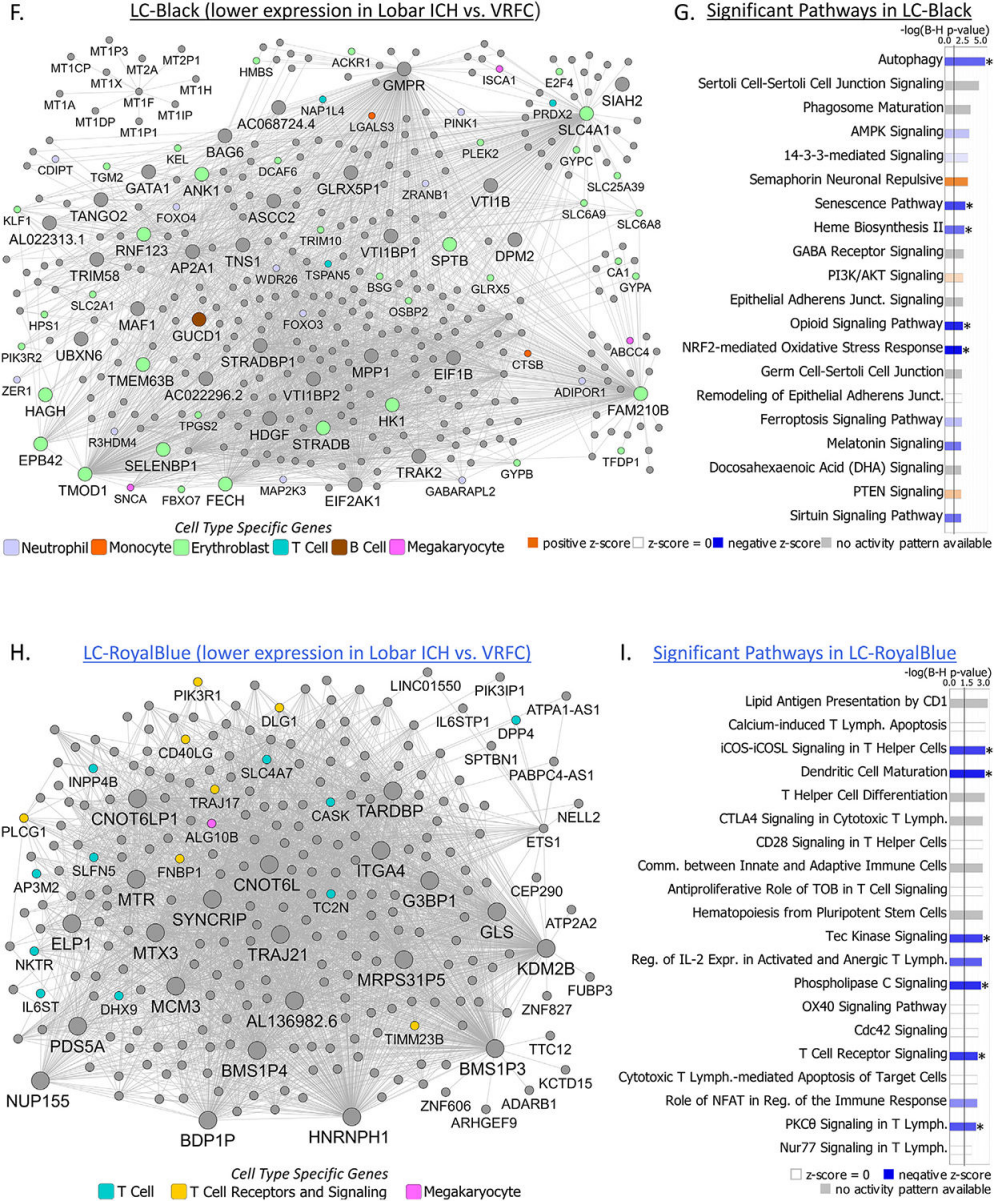
Network diagrams for the Lobar ICH modules LC-DarkGreen (A), LC-Pink (C), LC-Grey60 (E), LC-Black (F), and LC-RoyalBlue (H) show connectivity of hubs and genes within modules. Nodes represent genes within the module; edges represent connections based on co-expression between genes. Weaker connections and nodes with fewer connections have been filtered out to increase legibility. Larger nodes with large labels are hub genes, representing potential master regulators. Genes are grey by default and colored if they are cell type specific. Hubs, cell type specific genes, and other selected genes are labeled. In panel (A), the genes *SOS2* and *OSBPL8* were members of both the Neutrophil specific and T Cell Receptors and Signaling specific gene lists and were colored as Neutrophil-specific. In panel (C), the genes *SLA* and *APBB1IP* were members of both the Neutrophil specific and T Cell Receptors and Signaling specific gene lists and were colored as Neutrophil-specific. In panel (H), the gene *CASK* was a member of both the T Cell specific and the T Cell Receptors and Signaling specific gene lists and was colored as T Cell-specific. Pathway enrichment presented for LC-DarkGreen (B), LC-Pink (D), LC-Black (G), and LC-RoyalBlue (I); LC-Grey60 had no significant pathway enrichment. The top 20 relevant significant pathways are displayed. Significance threshold −log_10_(BH p value) of 1.3 (corresponds to BH p value of 0.05) depicted by a vertical black line. Higher −log_10_(BH p value) corresponds to lower (more significant) BH p value. Bar shading represents activity pattern prediction (blue for suppression/negative Z-score and orange for activation/positive Z-score) where darker color represents larger absolute Z-score; * represents statistically significant activity pattern prediction (Z ≥ 2, significant activation in Lobar ICH compared to Controls; Z ≤ −2, significant suppression in Lobar ICH compared to Controls). Reg. – Regulation; Lymph. – Lymphocytes; Expr. – Expression; Act. – Activated; NO – Nitric Oxide; ROS – Reactive Oxygen Species; Junct. – Junction; Comm. – Communication.

Modules of co-expressed genes significantly associated with ICH and the top 20 relevant canonical pathways significantly enriched in each module are presented in Fig. 6 for Deep ICH and Fig. 7 for Lobar ICH.

#### 3.3.1. Gene co-expression modules associated with Deep ICH

WGCNA identified 30 co-expressed gene modules plus one module of non-co-expressed genes in the DeepICHandVRFC network (SFigure 8). Hereafter we refer to modules from this network with the prefix DC- for Deep ICH and Control. DC-Grey60 and DC-LightGreen modules were uniquely significant for Group (Deep ICH vs. VRFC) and both were upregulated in Deep ICH (Fig. 4B – positive contrast regression beta; STables 7A,8A). DC-Grey60 was enriched in 185 pathways, with 102 activated (including Autophagy, TLR, iNOS, IL-6, and NF-κB Signaling) and 3 suppressed (including PPAR and PPARα/RXRα Activation) (Figs. 3A,6B; STables 2C,3C,4C). It was also enriched in BEX2 (brain expressed X-linked 2) Signaling, a pathway involved in neuroprotective autophagy, and showed a trend toward suppression in Deep ICH (Z = −1.3) (SFigure 9). DC-LightGreen was enriched in 4 pathways (including IL-10 Signaling and Fcγ Receptor-mediated Phagocytosis in Macrophages and Monocytes) (Figs. 3A,6D; STables 2D,3D,4D). DC-Grey60 and DC-LightGreen were enriched in Neutrophil specific genes; DC-LightGreen was enriched in Monocyte specific genes (Fig. 4B; STables 5DE).

#### 3.3.2. Gene co-expression modules associated with Lobar ICH

WGCNA identified 32 modules of co-expressed genes plus one module of non-co-expressed genes in the LobarICHandVRFC network (SFigure 10). Hereafter we refer to modules from this network with the prefix LC- for Lobar ICH and Control. LC-Black, LC-DarkGreen, LC-Grey60, LC-Pink, and LC-RoyalBlue modules were significant for Group (Lobar ICH vs. VRFC) (Fig. 4B; Stables 7B,8B). Of these, LC-Black and LC-Grey60 were also significant for Age. The remainder were unique to Group (Fig. 4B; STable 7B). LC-DarkGreen, LC-Grey60, and LC-Pink were upregulated in Lobar ICH (Fig. 4B – positive contrast regression beta), whereas LC-Black and LC-RoyalBlue were downregulated (Fig. 4B – negative contrast regression beta). LC-DarkGreen was enriched in 149 pathways, with 97 activated (including Autophagy, NGF, B Cell Receptor, and IL-6 Signaling) and 3 suppressed (PPAR, PPARα/RXRα Activation, and Antioxidant Action of Vitamin C) (Figs. 3A,7B; STables 2E,3E,4E). LC-Pink was enriched in 159 pathways, with 84 activated (including Amyloid Processing (SFigure 11), TLR, IL-1, IL-6, and IL-8 Signaling) and 3 suppressed (PPAR, PPARα/RXRα Activation, LXR/RXR Activation) (Figs. 3A,7D; STable 2F,3F,4F). LC-Grey60 had no canonical pathways passing BH-corrected p < 0.05 (STables 3G,4G). LC-Black was enriched in 74 pathways, of which 20 were suppressed (including Autophagy and NRF2-mediated Oxidative Stress Response) (Fig. 7G; STables 2G,3H,4H). LC-RoyalBlue was enriched in 34 pathways, 8 of which were suppressed (including several T-cell pathways) (Figs. 3B,7I; STables 2H,3I,4I). LC-DarkGreen and LC-Pink were enriched in Neutrophil specific genes; LC-Grey60 in Monocytes; LC-Black in Erythroblasts; and LC-RoyalBlue in T Cell and T Cell Receptor Signaling (Fig. 4B; STables 5F-J).

#### 3.3.3. Module Hubs

DC-Grey60 Hubs, LC-DarkGreen Hubs, and LC-Pink Hubs were enriched in Neutrophil specific genes, and LC-Black Hubs in Erythroblast specific genes (Fig. 4B; STables 5K-Q). DC-Grey60 Hubs were enriched in one pathway (Glycogen Degradation III), and LC-Pink Hubs in 68 (including iNOS Signaling, NRF2-Mediated Oxidative Stress Response, and PPARα/RXRα Activation) (STables 2IJ). LC-Black Hubs were enriched in GO term Cortical Cytoskeleton and LC-RoyalBlue Hubs in Protein Binding and Nucleotide Binding (STables 4JK). Hub genes by module are presented in Table 2, and complete hub biological findings are listed in STables 2IJ,3J-O,4JK,5K-Q.

### 3.4. Deep and Lobar ICH significant canonical pathways across Per-Gene and network analyses

Deep ICH gene lists and modules were significantly enriched in 235 canonical pathways, while Lobar ICH lists were enriched in 301. Of these, 209 pathways were common to both locations, leaving 26 unique to Deep ICH and 92 unique to Lobar ICH (SFigure 12). The Lobar ICH-unique pathways included Apoptosis Signaling, BMP Signaling (activated in two Lobar ICH modules), Neurotrophin/TRK Signaling (activated in one Lobar ICH module), VEGF Signaling, Necroptosis Signaling, and Amyloid Processing, as well as NRF2-mediated Oxidative Stress Response and Heme Biosynthesis II – both suppressed in LC-Black, a module with no significant overlap with any DC modules. The Deep ICH-unique pathways included activation of the Th2 Pathway, Interferon Signaling, and CREB Signaling in Neurons in the DC-Grey60 module; and BEX2 Signaling with a trend towards suppression in DC-Grey60.

### 3.5. Gene-level sex differences in Deep and Lobar ICH provide evidence for sex- and location-specific peripheral immune response to ICH

**Fig. 8.**
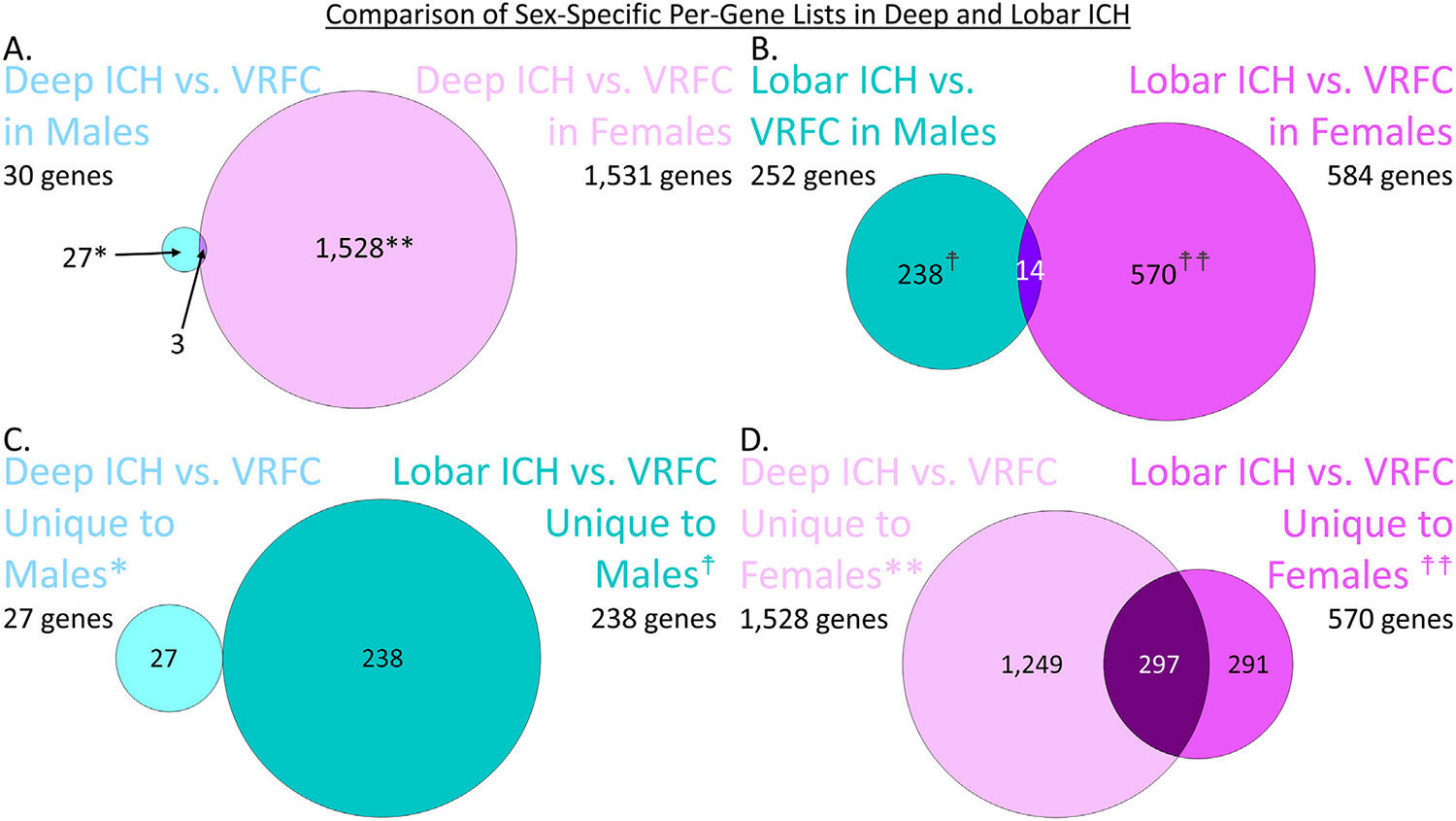
Comparison of Male and Female differentially expressed genes in Deep ICH vs. VRFC (A) and Lobar ICH vs. VRFC (B). Lists are genes passing p < 0.005 and FC > ∣1.2∣ for the specific contrast. Comparison of the Male unique gene lists in Deep ICH and Lobar ICH (C) and the Female unique gene lists in Deep ICH and Lobar ICH (D). *These genes are the Male-specific Deep ICH genes hereafter referred to as Male-DvC and analyzed in IPA and DAVID. **These genes are the Female-specific Deep ICH genes hereafter referred to as Female-DvC and analyzed in IPA and DAVID. ^†^These genes are the Male-specific Lobar ICH genes hereafter referred to as Male-LvC and analyzed in IPA and DAVID. ^††^These genes are the Female-specific Lobar ICH genes hereafter referred to as Female-LvC and analyzed in IPA and DAVID.

Expression of 30 genes was significant for the Deep ICH Males vs. VRFC Males; 1,531 for Deep ICH Females vs. VRFC Females; 252 for Lobar ICH Males vs. VRFC Males; and 584 for Lobar ICH Females vs. VRFC Females (Fig. 8AB; STable 9).

#### 3.5.1. Sex-specific genes in Deep ICH

**Fig. 9.**
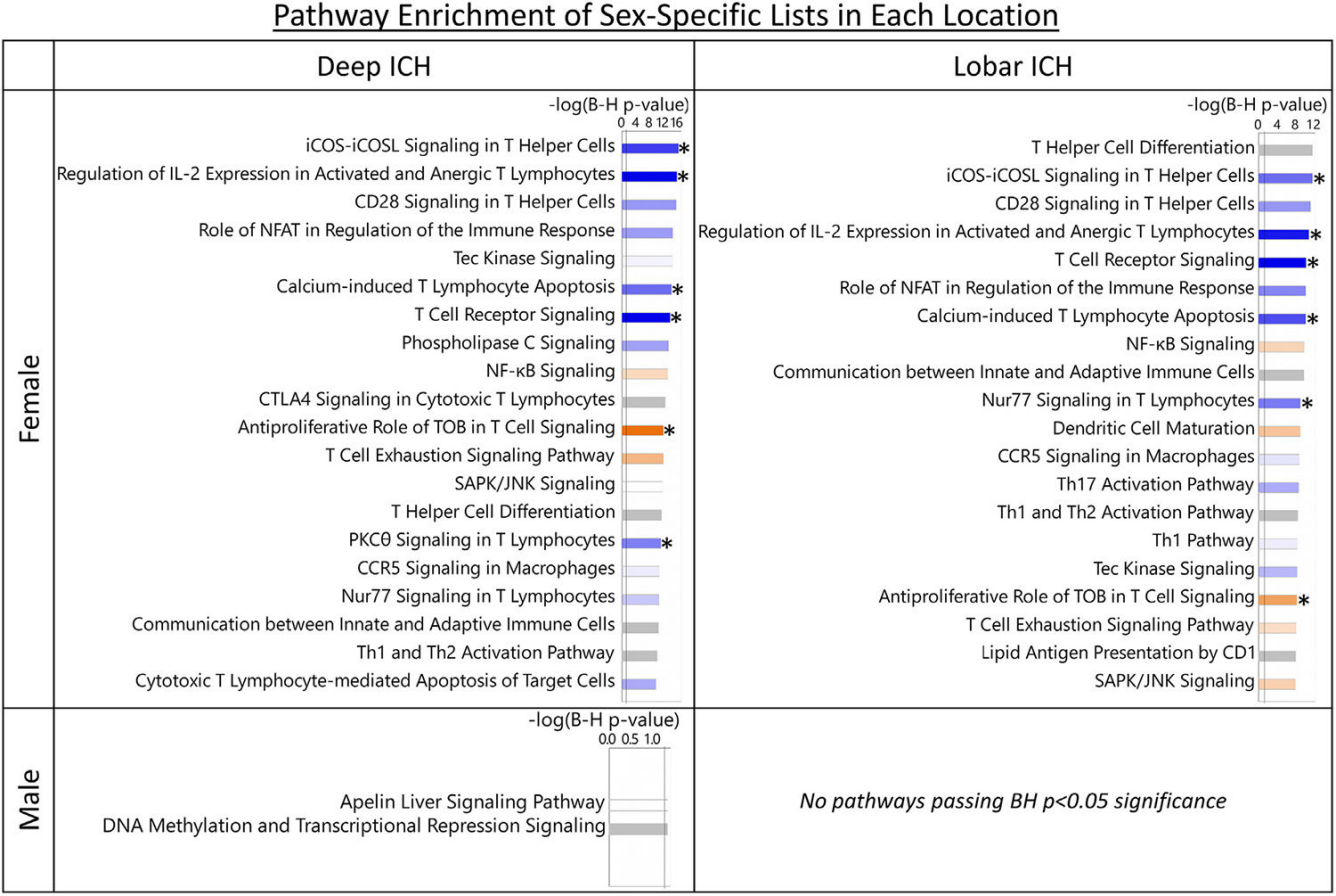
Pathway enrichment presented for sex-specific per gene lists in each location. The Y axis indicates Female lists (top) and the Male lists (bottom). The X-axis indicates Deep ICH (left) and Lobar ICH (right). The top 20 relevant significant pathways are displayed. No pathways were significant in Male-LvC. Bar shading represents activity pattern prediction (blue for suppression/negative Z-score and orange for activation/positive Z-score) where darker color represents larger absolute Z-score; * represents statistically significant activity pattern prediction (Z ≥ ∣2∣). Color legend as in Fig. 2.

Twenty-seven genes were unique to Deep ICH Males vs. VRFC Males (hereafter called Male-DvC for Male Deep ICH vs. Male Control) when compared to Deep ICH Females vs. VRFC Females (Fig. 8A; STable 9A). The 27 genes were significantly enriched in 2 pathways: DNA Methylation and Transcriptional Repression Signaling, and Apelin Liver Signaling Pathway (Fig. 9; STable 2K,3P). The Male-DvC genes had no significant enrichment in cell type-specific lists (SFigure 13; STable 5R). One thousand five hundred twenty-eight genes were unique to Deep ICH Females vs. VRFC Females (hereafter called Female-DvC for Female Deep ICH vs. Female Control) when compared to Deep ICH Males vs. VRFC Males (Fig. 8A; STable 9B). The 1,528 genes were enriched in 148 pathways, with 7 activated (including Antiproliferative Role of TOB in T Cell Signaling and Fcγ Receptor-mediated Phagocytosis in Macrophages and Monocytes) and 6 suppressed (including several T-cell pathways) (Fig. 9; STables 2L,3Q,4L). The Female-DvC genes were also enriched in Neutrophil, T Cell, and T Cell Receptor and Signaling specific gene lists (SFigure 13; STable 5S).

#### 3.5.2. Sex-specific genes in Lobar ICH

Two hundred thirty-eight genes were unique to Lobar ICH Males vs. VRFC Males (hereafter called Male-LvC for Male Lobar ICH vs. Male Control) when compared to Lobar ICH Females vs. VRFC Females (Fig. 8B; STable 9C). The 238 genes were not enriched in biological pathways or cell type specific lists, though it was enriched in GO term Protein Binding (Fig. 9; SFigure 13; STables 4M,5T). Five hundred seventy genes were unique to Lobar ICH Females vs. VRFC Females (hereafter called Female-LvC for Female Lobar ICH vs. Female Control) when compared to Lobar ICH Males vs. VRFC Males (Fig. 8B; STable 9D). The 570 genes were enriched in 65 pathways, with 6 activated (including Antiproliferative Role of TOB in T Cell, TLR, and iNOS Signaling) and 6 suppressed (including several T-cell pathways) (Fig. 9; STables 2M,3R,4N). Female-LvC was also enriched in Neutrophil, T Cell, and T Cell Receptor and Signaling specific gene lists (SFigure 13; STable 5U).

#### 3.5.3. Comparing sex-specific genes in Deep and Lobar ICH

There was no overlap between the Male-specific genes in the Deep and Lobar locations (Fig. 8C). Two hundred ninety-seven genes overlapped between the Female-specific genes from the two ICH locations, leaving 1,249 unique for Deep ICH and 291 unique for Lobar ICH (Fig. 8D).

## 4. Discussion

**Fig. 10.**
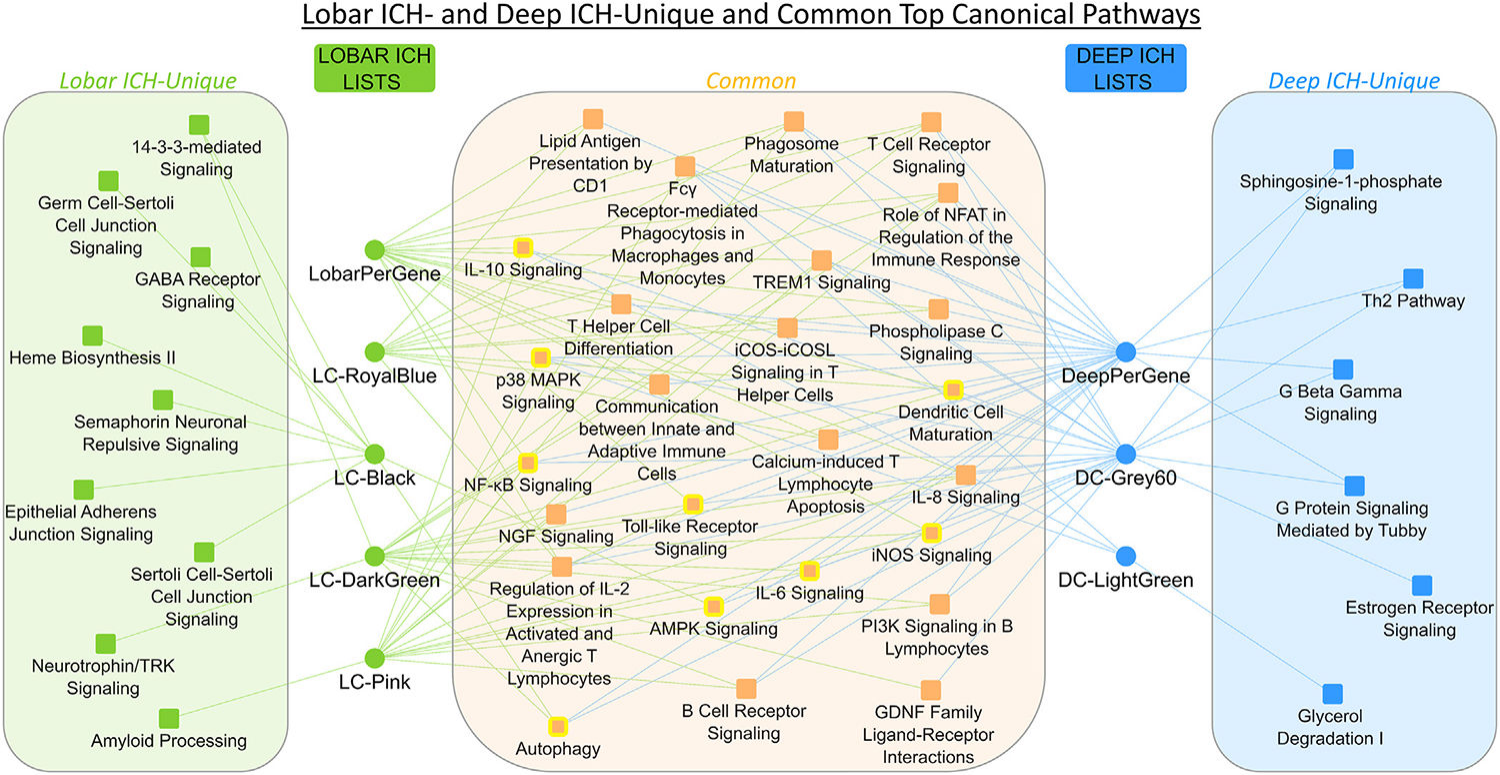
Top pathways in each analysis showcase common and different responses in Deep ICH and Lobar ICH. Listed pathways represent the top 5 most significant in each gene list and module, the top 5 most significant unique to each location not already listed, and other selected pathways. Circles represent modules/per-gene lists associated with each location; squares represent canonical pathways. Pathways with yellow outline have DeepVsLobar (Deep ICH vs. Lobar ICH) gene involvement. This involvement indicates that even in common pathways, there may be differences between locations. LC-Grey60 was omitted from the figure since it did not have BH p < 0.05-passing pathways.

Differences in the peripheral blood transcriptome architecture were identified for Deep and Lobar ICH that differentiated the groups. These include differentially expressed genes and location-specific gene co-expression modules; two modules were associated with Deep ICH and five with Lobar ICH. They were enriched in many immune cell specific gene lists, pathways, functions, and GO terms common to Deep and Lobar ICH as well as responses unique to each location (Fig. 10). Common responses included immune, inflammatory, oxidative stress, growth factor (GF), and angiogenesis related processes. Deep ICH-unique responses included CREB Signaling, dopaminergic neuronal cell death, and Th2 responses; while Lobar ICH-unique included RNA processing, various protein processing, and amyloid related responses. These data provide evidence for molecular differences between Deep and Lobar ICH which reinforce the need for location-stratified analyses and clinical trials to identify potential location-specific treatments.

### 4.1. Blood cell response to ICH

#### 4.1.1. Myeloid cell response

Neutrophils respond to human ICH and infiltrate hematoma and perihematomal brain regions, potentially contributing to damage through pro-inflammatory signaling, Reactive Oxygen Species (ROS), and Blood-Brain Barrier (BBB) breakdown.^31-33^ Later, polarized neutrophils become neuroprotective, partially through enhanced iron scavenging.^34^ Through the enrichment of granulocyte (mainly neutrophil) specific genes, we show evidence for a robust neutrophil response to ICH common to both Deep and Lobar ICH. (Fig. 4). Co-expression modules were significant for the Granulocyte Adhesion and Diapedesis pathway, which regulates neutrophil movement from blood vessels to target tissue. The fMLP Signaling in Neutrophils pathway was common to Deep and Lobar ICH. fMLP activates neutrophils, which induces ROS generation, cell migration, and enzyme secretion.^35^ *FPR1* and *FPR2* initiate fMLP signaling in neutrophils^36^ and were up-regulated in both ICH locations. Neutrophil-related functions such as degranulation, activation, migration, and recruitment were also common to both Deep and Lobar ICH.

Monocytes also infiltrate the brain after ICH,^14,37^ likely contributing to post-stroke damage early^37-39^ and recovery via hematoma clearance later.^2,40,41^ Both Deep and Lobar ICH gene lists were enriched in Monocyte specific genes (Fig. 4) as well as functions like Activation of Monocytes, Cell Movement of Macrophages, and Differentiation of Macrophages. Accumulation of Alternatively Activated Macrophages was unique to Deep ICH, while Accumulation of Inflammatory Monocytes was unique to Lobar ICH. Both Deep and Lobar ICH lists were enriched for Fcγ Receptor-mediated Phagocytosis in Macrophages and Monocytes, which contributes to hemoglobin clearance in Subarachnoid Hemorrhage (SAH)^42^ and may also occur in ICH. We have previously shown DE genes were associated with inflammatory pathways in blood monocytes from ICH patients^43^ and showed some monocyte-specific genes correlated with ICH and edema volumes.^44^ Future temporal studies of gene expression are needed to elucidate the peripheral monocytes’ contribution to early injury and later recovery following ICH.

#### 4.1.2. Lymphocyte response

CD4^+^ Helper T (Th) and CD8^+^ Cytotoxic T cells are associated with ICH, with Th cells migrating to perihematomal brain regions.^14,45^ After ICH, Th1 cells contribute to inflammation, BBB breakdown, and neuronal apoptosis, while Th2 cells suppress the inflammatory response.^45^ Cytotoxic T cells also contribute to brain injury after ICH^46^ and initiate BBB breakdown.^47^ Regulatory T Cells, however, are associated with neuroprotection after ICH, potentially by protecting the BBB.^48,49^ Our data support a T Cell response to Deep and Lobar ICH. The DE genes in Deep ICH and Lobar ICH, as well as a Lobar ICH module were significantly enriched in T Cell and T Cell Receptor Signaling specific genes (Fig. 4). We have previously shown suppressed T Cell Receptor Signaling with increasing ICH and Edema volume in peripheral whole blood following human ICH.^44^ However, in this study we found lists associated with both suppression and activation of the T Cell Receptor Signaling pathway in both Deep and Lobar ICH (Fig. 3B). Our data also showed T Cell surface receptors (TCR), including *CD28* and *CD3E*, and various TCR subunits were down-regulated in both ICH locations compared to controls. TCR and CD3 proteins form a complex on T Cells^50^ beginning a signaling cascade through MAP Kinases for initial naive T cell activation.^51^ Complete activation can occur through co-stimulation of the CD28 receptor.^51^ Src-family kinase (SFK) LCK functions downstream of many T Cell surface receptors and SFKs are involved in T cell development, proliferation, survival, and function.^52^ *LCK*, and downstream targets *ZAP70* and the PI3K complex (via *PIK3R1*), were down-regulated in Deep ICH vs. Control. Though both Deep and Lobar ICH per-gene lists showed suppression of the overall T Cell Receptor Signaling pathway, some inflammatory outputs were upregulated in Lobar ICH via *CHUK*, which activates NF-κB (SFigure 5).^53,54^ NFAT was downregulated in Deep ICH via decreased levels of *NFATC2* and *NFATC3*, and is involved in T Cell Activation.^55^ This implies that, despite a common decrease in overall T Cell Receptor Signaling, there may be differences in T Cell effects on the inflammatory responses between ICH locations.

The Th1 and Th2 Activation, Th1 (activated in both locations), and Th17 Activation Pathways were also common to Deep and Lobar ICH, as were *IFNGR1* and *IFNGR2* (IFNγ Receptors). Though the role of IFNγ signaling in Th1 differentiation is debated,^56^ it may be important in autocrine Th1 differentiation and function.^57-59^ The Th2 Pathway itself was unique to and activated in Deep ICH. GO term Positive Regulation of IL-4 Production was significant in Deep ICH. *IL4R*, *JAK2*, and *JAK3* (upregulated in Deep ICH) could lead to IL-4 Receptor activation of JAK signaling cascades for transcriptional regulation in T cells. Notably, IL-4 helps initiate Th2 differentiation and development.^60,61^ Differing responses of T-helper subtypes may also contribute to differences in the inflammatory response between ICH locations.

#### 4.1.3. Erythroblast response

Erythroblasts are immature nucleated red blood cells (NRBC) found in peripheral blood after ICH.^62,63^ A Lobar ICH module downregulated in ICH was enriched in erythroblast specific genes and biofunctions while Deep ICH was not. Erythropoietin signaling, which regulates RBC generation,^63^ was common between locations. A detailed discussion of the erythroblast response is in the Supplemental Manuscript.

### 4.2. Inflammatory signaling

#### 4.2.1. Neuroinflammation after ICH

After ICH, extravascular blood initiates inflammation, affecting ionic membrane pumps and contributing to cerebral edema formation and secondary injury^1,14,45^ which is exacerbated by BBB breakdown.^45^ Indeed, Neuroinflammation Pathways were activated in both Lobar and Deep ICH. Inflammasomes, including NLRC4^64,65^ and NLRP3,^66^ contribute to neuroinflammation by activating pro-inflammatory enzymes and cytokines following stroke.^67^ The Inflammasome pathway was predicted activated in both Deep and Lobar ICH. *NLRC4* was present in Lobar (as a hub) and Deep modules, and *NLRP3* was present in Deep and Lobar ICH modules. P2X7R, an activator of the NLRP3 inflammasome, is a potential target for ICH treatments,^68^ and P2X7R-siRNA (small interfering RNA) decreased NLRP3 Inflammasome activity and improved outcomes in a rat ICH model.^69^ *NLRC3*, decreased in Lobar ICH, inhibits NLRP3 Inflammasome activity, and as such may be a potential treatment target.^70^ NLRP12 is an NLR protein which has been reported as both pro- and anti-inflammatory in various *in vitro* and animal models.^71^ *NLRP12* was present in both Deep and Lobar modules.

Lipopolysaccharide (LPS) is a Pathogen-Associated Molecular Pattern (PAMP) molecule found in the cell wall of Gram-negative bacteria.^72^ It induces pro-inflammatory signaling^72-74^ and is elevated in serum after human ICH.^75^ In this study, LPS was identified as an upstream regulator for several Deep and Lobar ICH modules and was generally predicted to be activated. This implicates LPS as a potential pro-neuroinflammatory molecule after ICH. Additionally, the Complement System pathway and Activation of Complement Factor function were common to both ICH locations. The complement cascade could play a role in post-ICH damage through pro-inflammatory edema exacerbation, cytokine release, and induction of iron toxicity. However, it also could play a healing role by clearing apoptotic cells, facilitating hematoma clearance, and promoting neurogenesis.^1,76,77^ As such, complement’s involvement in ICH damage and repair needs more study.

#### 4.2.2. Cytokine signaling

After ICH, a large number of cytokines are released that contribute to secondary injury by compromising the BBB, exacerbating edema formation and immune cell invasion.^14,76,78^ In this study, many cytokine signaling pathways were overrepresented in both Deep and Lobar ICH such as pro-inflammatory IL-17, IL-23, TNFR1, TNFR2, and IL-1 Signaling^76^ and anti-inflammatory IL-4, IL-10, and TGF-β Signaling (Fig. 3A).^76^ The balance between these pro- and anti-inflammatory cytokines likely contributes to the damage-repair balance after ICH regardless of location. Modulation of this system to treat ICH could be complex. More details are presented in the Supplemental Manuscript.

#### 4.2.3. Oxidative stress

After ICH, neutrophil degranulation, mitochondrial dysfunction, and iron from hematoma breakdown can contribute to oxidative stress, which can exacerbate BBB breakdown.^79^ The Production of Nitric Oxide (NO) and ROS in Macrophages pathway was common and predicted activated in both ICH locations. The Deep ICH hub gene *SPI1* (encoding PU.1) regulates NADPH oxidase genes^80^ which contribute to ROS generation.^81^ iNOS Signaling was predicted to be activated in Deep and Lobar ICH. iNOS generates reactive nitrogen species (RNS)/ROS after ICH. Its knockout reduced edema volumes.^82^ Common functions between the two ICH locations also included Biosynthesis of ROS, Generation and Synthesis of ROS, and Metabolism of ROS. Targeting oxidative stress after ICH might reduce injury and improve outcomes in both locations.

Though NRF2-Mediated Oxidative Stress Response was a Lobar-unique pathway, *NRF2* (aka *NFE2L2*) itself was a member of a significant Deep ICH module. In animal models of Deep striatal ICH, NRF2 was neuroprotective and involved in hematoma clearance.^83-85^ NRF2 promotes expression of neuroprotective genes and could increase antioxidant activity after ICH.^79^ NRF2 also upregulates expression of *HMOX1* (encoding HO-1, heme oxygenase 1), associated with Deep ICH. HO-1 promotes antioxidant generation and degrades heme.^86^ HO-1 reduces oxidative stress by generating CO (which inhibits NADPH ROS generation) and biliverdin (which scavenges ROS and RNS).^87^ Additionally, the Antioxidant Action of Vitamin C pathway was suppressed in both locations. Ischemic stroke patients with lower Vitamin C levels had worse outcomes.^88^ Since Vitamin C levels decrease after cerebral hemorrhages^89^ this may contribute to worse outcomes and represent an ICH treatment target. Additionally, Lobar ICH was associated with downregulation of 7 Metallothionein-encoding genes, 8 Metallothionein pseudogenes, and one Metallothionein-like gene. Metallothionein is involved in wound healing in the CNS^90^ and is upregulated in brain after experimental ICH.^91,92^ Metallothionein is an antioxidant after ICH-related iron release and may be neuroprotective.^91,93^ Downregulation of antioxidant molecules and pathways after ICH may contribute to oxidative stress induced damage, and as such pose promising potential treatment targets.

#### 4.2.4. Growth factor (GF) signaling

Higher serum levels of various GFs are associated with better Modified Rankin Scale (mRS; a severity scale) outcomes in human ICH patients at 3 months.^94,95^ In this study, many GF signaling pathways were common to Deep and Lobar ICH as discussed in the Supplemental Manuscript. Additionally, there were three GF pathways unique to Lobar ICH: Angiopoietin Signaling, Neurotrophin/TRK Signaling, and VEGF Signaling. High Angiopoietin-1 and VEGF serum levels have been associated with good outcomes after ICH,^95^ though other studies show a deleterious effect of high VEGF levels.^96^ In animal ICH models, Neurotrophin treatment improved recovery and neurogenesis^97^ and reduced neuronal apoptosis.^98^.

#### 4.2.5. Autophagy

Autophagy regulates the degradation of unneeded or malformed proteins and organelles via lysosomes to maintain normal cell function.^99^ Oxidative stress can induce autophagy.^100^ After ICH, iron-oxidized species may contribute to brain injury by activating autophagy.^99^ In this study, the Autophagy pathway was common between locations. Additionally, biofunction Autophagy of Neurons was significant in Lobar ICH. *TLR4* was common and TLR Signaling was significant and usually activated in Deep and Lobar ICH. LPS induces autophagy via activation of TLR4.^101^ Resatorvid treatment inhibited autophagy and neuron loss in rat TBI possibly via TLR4 signaling.^102^ Some autophagy-related genes (ATG) aid in the formation of autophagosomes.^103^ Several were decreased in Lobal ICH vs. VRFC including *MAP1LC3B* (aka *LC3B* and *ATGF8*), *GABARAP*, *GABARAPL2* (human Atg8 orthologs), and *ATG9A*. *ATG12* was increased Lobar ICH vs. VRFC. ATG12 initiates the ATG12 conjugation system,^103^ which is involved in autophagosome maturation.^104^ ATG9A is required for autophagosome formation^103^ and may help transfer materials to the developing autophagosome.^105^ Autophagy was predicted significantly suppressed in one module in Lobar ICH, and included downregulated *SQSTM1* (an autophagy receptor connecting autophagosomes to their cargo),^106^ *MAP1LC3B* (involved in phagophore membrane elongation),^103,107,108^ *GABARAP*, and *GABARAPL2* (both involved in late stage autophagosome formation).^108^ This suggests that regulation of autophagy may be particularly important in Lobar ICH.

### 4.3. Cell death

#### 4.3.1. Cell death pathways

Various cell death mechanisms can be induced after ICH including mechanical stress, inflammatory pathways, and toxic molecules like iron and ROS, among other factors.^2,109,110^ Thus, apoptosis, pyroptosis, necroptosis, ferroptosis, autophagy and necrosis can contribute to cell loss after ICH.^109-111^ Genes from each of these pathways were associated with Deep and/or Lobar ICH. The function “Apoptosis” was common between locations but was activated in some modules and suppressed in others, indicating complex regulation of cell death in ICH. Detailed discussion of our Apoptosis findings can be found in the Supplemental Manuscript.

Ferroptosis Signaling Pathways trended towards activation in Deep ICH (Z = 1.89) and trended towards suppression in Lobar ICH (Z = −0.91). Ferroptosis occurs after hemorrhagic strokes and is induced by lipid ROS via intracellular iron.^109,112^ *ALOX5* (aka 5-LOX and Arachidonate 5-Lipoxygenase), up-regulated in Deep ICH, is a major contributor to buildup of oxidized lipids.^109,112,113^ N-acetylcysteine, which inhibits ALOX5 oxidation of lipids, improved outcomes in experimental ICH.^109,112^ Intracellular iron can also be sequestered into the iron storage protein complex ferritin.^109^ *FTL*, a part of the ferritin complex, was down-regulated in Lobar ICH. Promoting ferritin gene expression may produce more storage for free intracellular iron, reducing the oxidation of lipids and limiting ferroptosis.^114^ Ferroptosis inhibitor ferrostatin-1 reduced ferroptosis and improved outcomes in experimental ICH.^109,115,116^ Additionally, *HSF1* and *HSPB1*, downregulated in Lobar ICH, normally aid in the removal of iron and lipid ROS from cells and inhibit ferroptosis.^109^ Modulating ferroptosis particularly in Lobar ICH could improve outcomes.

Necroptosis was uniquely enriched in Lobar ICH with the Necroptosis Signaling Pathway and biofunction Necroptosis of Oligodendrocytes being significant. Necroptosis can be induced following ICH through TNF signaling, TLR signaling, and interferons.^109^ *TNFRSF10B* (TNF Receptor Superfamily 10b), *IFNAR1* (Interferon Alpha And Beta Receptor Subunit 1), and *TLR4* were increased in Lobar ICH. *TAB1*, which was higher in Lobar ICH than Deep ICH, forms part of the TNFR1 signaling complex in Necroptosis Signaling. TAB1, in addition to TAB2 and TAB3 (regulated in Deep and Lobar ICH in this study), form a complex with TAK1 in response to TNFR1 and TLR signaling.^117^ *CFLAR* (coding cFLIP), also associated with Lobar ICH, regulates necroptosis.^118^ Higher proportions of the cFLIP_S_ isoform promotes necroptotic pathways over cell survival or apoptosis.^118^ *PELI1* was a Lobar ICH gene that is an E3 ubiquitin ligase which promotes necroptotic cell death pathways and modulates cFLIP expression to inhibit apoptosis.^119^ The data suggest that potential Necroptosis treatments should target Lobar ICH.

Autophagy dysregulation can result in autophagic cell death.^120^ The Autophagic Cell Death function was regulated in Lobar ICH and included *TP53INP1*. TP53INP1 is present in the autophagosome when autophagy is induced and can promote autophagic cell death pathways.^121^

Overall, these results show common cell death signaling responses to both Lobar and Deep ICH through apoptosis and ferroptosis, while also providing evidence for potential differences in apoptosis, ferroptosis, necroptosis and autophagic cell death between ICH locations.

#### 4.3.2. Dopaminergic pathways implicated in Deep ICH

The BEX2 Signaling Pathway was associated with Deep ICH. Cell Death of Dopaminergic Neurons, a functional output of this pathway, showed potential upregulation via downregulated BEX2 (SFigure 9). BEX2 is a transcription factor involved in isoflavone induced autophagy, clearing toxins and preventing dopaminergic cell death in neuroblastoma cell lines.^122^ Isoflavone treatment may increase BEX2 induced autophagy which may prevent dopaminergic neuron apoptosis.^122^ Since the striatum is densely innervated by dopaminergic fibers, this might account for the association of dopamine pathways with Deep ICH.^123^ *PLXNC1*, also associated with Deep ICH, plays a role in dopaminergic circuit formation.^124^ *EGLN1/PHD2* was a Deep ICH hub gene in our study and is involved in Loss of Induced Pluripotent Stem Cell Derived Dopaminergic Neurons. PHD2 is known to play a role in iron homeostasis in dopaminergic neurons.^125^

### 4.4. Protein processing including amyloid processing pathways are unique to Lobar ICH

#### 4.4.1. Protein ubiquitination

Ubiquitin was associated with Lobar ICH. It is a small protein that covalently links to target proteins, marking them for degradation.^126^ Ubiquitination regulates neuroinflammation and autophagy, and is required for normal neuronal development and function.^126-129^ Ubiquitin proteases, ligases, and deubiquitination proteins regulate injury in experimental ICH, potentially via oxidative stress and neuronal apoptosis.^130-132^ We have previously shown ICH and ICH relative perihematomal edema volume were significantly enriched in protein ubiquitination pathways.^44,133^ In this study a number of down-regulated genes in Lobar ICH were strongly enriched in ubiquitination terms, whereas Deep ICH was not. The Lobar-associated pathways and functions included Protein Ubiquitination Pathway, Ubiquitination, Ubiquitination of Protein, Deubiquitination of Protein, Polyubiquitination, Ubiquitin-Dependent Protein Catabolic Process, Ubiquitin-Protein Transferase Activity, and Ubiquitin Protein Ligase Binding. Moreover, LC-Black genes had no significant overlap with the genes in any of the two Deep ICH modules (data not shown), signifying it is a highly Lobar-specific module. *BAG6*, a Lobar ICH hub gene, is associated with ubiquitination of mislocalized proteins^134^ and newly synthesized, defective protein products.^135^ *SIAH2*, another Lobar ICH hub gene and E3 ubiquitin ligase, is induced by hypoxia and impairs HIF-1α degradation, and thus changes HIF-1α target expression.^136^ *SPOPL*, another Lobar ICH hub gene, associates with SPOP to downregulate E3 ubiquitin ligase activity.^137,138^ The heavy involvement of protein ubiquitination processes in genes that were down-regulated in Lobar ICH and upregulation of ubiquitin inhibitors suggests wide-spread down-regulation of protein ubiquitination following Lobar ICH.

Ischemia-linked oxidative stress results in mass-misfolding of proteins, with ubiquitination working to clear these misfolded proteins.^139^ One Lobar ICH module was significant for the Unfolded Protein Response. Ubiquitination E2, E3, and deubiquitinating enzymes play a role in degradation of Aβ,^140^ and Aβ also regulates the ubiquitin system. It competitively binds to ubiquitin,^141^ inhibits ubiquitin-mediated proteolysis,^142^ and impairs proteasome function.^140,143^ When Aβ is bound to Ubiquitin, it is broken down more slowly (through the Insulin Degrading Enzyme), but it is also less likely to form protein deposits.^141^ Since Ubiquitin helps clear Aβ, it may be a location-specific treatment target for Lobar ICH caused by CAA in order to prevent future hemorrhages. Apomorphine treatment decreases intraneuronal Aβ and increases proteasome activity.^140,144^ It is possible that it has similar effects on vascular Aβ deposits. In animal models and *in vitro*, Sulforaphane reduced Aβ levels,^140,145,146^ potentially through upregulation of proteasomal subunits,^140,145^ which could reduce Aβ levels in CAA.

#### 4.4.2. Ubiquitin-like modifications

Various ubiquitin-like post-translational modifications can also be added to proteins. Lobar ICH was associated with two forms of ubiquitin-like post-translational modifiers (SUMOylation and NEDDylation) while Deep ICH was not. One ubiquitin-like modifier gene, *UBA3* (ubiquitin-like modifier activating enzyme 3), was a Lobar ICH hub gene. This specific E1 enzyme associates with AppBp1 (amyloid beta precursor binding protein) to activate NEDD8, another ubiquitin-like post-translational modifying protein.^147,148^ Additional discussion of the Lobar ICH specific Ubiquitin-Like modifications can be found in the Supplemental Manuscript.

#### 4.4.3. Amyloid proteins and protein processing

CAA is caused by the deposition of amyloid in the meningeal and intracerebral vasculature. At least 6 proteins (encoded by genes *APP*, *CST3*, *TTR*, *GSN*, *PRNP*, and *ITM2B*) have been shown to form amyloid fibrils and contribute to CAA in humans.^149,150^ Amyloid Precursor Protein (*APP*) was associated with a Lobar ICH module in this study. Though it was not significant in the per gene analysis, it was a member of a negative beta coefficient module (*APP* FC = −1.02). *GSN*, the causative protein in Finnish amyloidosis,^149^ was associated with Lobar ICH. *PSEN1* was associated with both Lobar and Deep ICH. It acts as the catalytic component of γ-secretase, which cleaves precursors into Aβ.^151^ None of the 6 causative CAA genes were associated with Deep ICH.

Lobar ICH was also associated with pathways, functions, and GO terms relating to Amyloid processing. As CAA is caused by vascular amyloid deposition, amyloid processing likely plays a role in Lobar ICH. Indeed, one Lobar ICH module was enriched for genes in the Amyloid Processing pathway, which encompasses molecular signaling in response to Amyloid buildup with suggested activation of Microtubule Instability and suggested suppression of Axonal Transport (SFigure 11). Biofunction Amyloidosis was enriched in Deep and Lobar ICH. No other amyloid terms were enriched in Deep ICH. One Lobar ICH module was significant for Transport of Protein function, and another trended towards significance for the RAGE Receptor Binding GO term, which is involved in Aβ clearance.^149,152,153^ Another Lobar ICH module was nearly significant for the function Degradation of Protein Fragment with hub gene *BAG6*, which is involved in ubiquitin-mediated protein degradation.^154^

One Deep ICH module was enriched in Phagocytosis by Microglia and Phagocytosis by Neuroglia, neither being present in Lobar ICH. Microglial phagocytosis is an Aβ clearance mechanism^155^ which could differ between Lobar and Deep locations. *LRP1*, also associated with Deep ICH, is a clearance receptor that aids transport of Aβ out of the brain and into blood vessels.^155^ *CTSB* and *CTSS*, both associated with Lobar ICH, are cysteine proteases involved in the degradation of Aβ.^155^ Neither associated with Deep ICH. Reelin Signaling in Neurons pathway (suppressed in Lobar ICH) inhibits Aβ’s ability to form amyloid fibrils.^156^ *MMP9* and *MBP* genes, which are involved in Aβ degradation, were associated with Lobar and Deep ICH. These differentially expressed pathways may help explain why CAA affects cortex and not deep structures and may play a role in the progression of CAA and the pathogenesis of Lobar ICH.

### 4.5. Enrichment in RNA processing, trafficking, splicing, and degradation is Lobar ICH-specific

Splicing dysfunction is associated with many diseases, and the minor spliceosome is implicated in stress-induced gene expression regulation.^157^ We have previously shown differential alternative splicing in ICH^158,159^ and have found enrichment in alternative splicing processes at the gene and network level in ICH.^44,133^ Here, we find enrichment in various RNA processing, splicing, and degradation processes in Lobar (mainly LC-RoyalBlue) but not Deep ICH, with genes being down regulated in Lobar ICH vs. Control. Moreover, LC-RoyalBlue (like LC-Black) had no significant overlap with any of the two Deep ICH modules, signifying it too is a highly Lobar-specific module. Though it is likely splicing plays a role in Deep ICH, these results point to potential differential alternative splicing between ICH locations. Alternative splicing level analyses could unveil additional differences between Deep and Lobar ICH responses. Additional discussion of these findings is in the Supplemental Manuscript.

### 4.6. Platelets and blood coagulation

Blood coagulation and platelets play a key role in ICH onset and progression. Platelets form the initial vessel plug and seal, and the coagulation cascade forms a reinforcing fibrin clot.^160^ Coagulopathies and platelet dysfunction are potential causes of hemorrhagic stroke and can lead to hematoma expansion.^160-162^ In this study, both Deep and Lobar ICH were enriched in platelet functions including Aggregation of Blood Platelets, Degranulation of Blood Platelets, and Function of Blood Platelets. Platelets bind to the injured vessel surface and are activated, degranulating and aggregating other circulating platelets.^163^ Both Deep and Lobar ICH lists were also enriched in Thrombopoietin Signaling. Thrombopoietin plays a major role in platelet production, and is regulated (cleared) by existing platelets and megakaryocytes.^164^ Deep ICH was also uniquely enriched in the GP6 Signaling Pathway. GP6 is a collagen receptor only found on platelets that is involved in platelet activation, dense granule secretion, and thrombus formation.^165^

After plug formation, coagulation forms a fibrin clot to reinforce the plug.^163^ Deep and Lobar ICH were enriched in the function Coagulation of Blood; one Deep ICH module’s hubs were enriched in Anticoagulation of Blood with gene *ORM1*. ORM1 decreases the body’s ability to make thrombin, therefore inhibiting clot formation.^166^ Polymorphisms in *ORM1* have an impact on warfarin anticoagulant activity, making it a potential marker for determining an individual’s dosage.^167^ *F5* (Factor 5; associated with both locations) increases clotting by promoting thrombin formation in the common coagulation pathway.^160,168^ Thrombin Signaling was significant in both locations and usually activated. One Lobar module had predicted suppression of the pathway. This tight regulation of thrombin is important in the ICH response as over-coagulation could lead to thromboembolism and undercoagulation could cause hematoma expansion.^160^ *F13A1* (associated with Lobar ICH; FC = 1.06 in Lobar ICH vs. VRFC) codes for Coagulation Factor XIII A Chain. FXIII aids in crosslinking fibrin and stabilizing the clot.^160,169^ ICH therapeutics targeting platelets and clotting could improve outcomes by preventing hematoma expansion. Vitamin K, recombinant activated Factor 7, and prothrombin complex concentrate are common treatments for ICH caused by coagulopathies. Platelet transfusions have also limited hemorrhage enlargement. However, a balance between pro- and anti-coagulation mechanisms must be sought to help ensure safe treatments.^160^

### 4.7. Direct comparison of Deep and Lobar ICH implicates different molecular responses to each

Though the direct comparison of Deep and Lobar ICH was not enriched in any pathways, a number of the differentially expressed genes (including the following) are involved in immune and inflammatory pathways (SFigure 7). *TRAF3* (aka TNF Receptor Associated Factor 3) is involved in Neuroinflammation Signaling, Autophagy, NF-κB Signaling, Protein Ubiquitination, Regulation of Cytokine Production, Generation of Th1 Cells, and B Cell Activating Factor Signaling. *TAB1* (aka TGF-Beta Activated Kinase 1 (MAP3K7) Binding Protein 1) is involved in IL-(1,6,10), iNOS, TLR, PPAR, TGF-β, NF-κB and p38 MAPK Signaling pathways, and Dendritic Cell Maturation. *CD226* is involved in Crosstalk Between Dendritic Cells and NK Cells, NK Cell Signaling, Frequency of iNKT1 and iNKT2 Cells, Regulation of Immune Response, and Cytokine Production. *ABL2* (aka ABL Proto-Oncogene 2, Non-Receptor Tyrosine Kinase) is involved in PDGF Signaling, RhoA Signaling, IL-15 Production, Maturation of Dendritic Spines, and Maturation of Synapse. *MRPL2* (aka Mitochondrial Ribosomal Protein L2) is involved in Mitochondrial Translation, RNA Binding, and poly(a) RNA Binding. *RSBN1* is a T Cell specific gene, and *EXOC3L4* is a Megakaryocyte specific gene. Overall, 21/36 of the differentially expressed genes in Deep vs. Lobar ICH were higher in Lobar ICH (including *TRAF3*, *TAB1*, *CD226*, *MRPL2*) and 15 were higher in Deep ICH (including *ABL2*, *RSBN1*, *EXOC3L4*). Details are in STables 5C,6ABC.

### 4.8. Sex differences in immune response to Deep and Lobar hemorrhages

A pilot analysis of sex differences in Deep and Lobar ICH was performed. We have previously shown Females tend to have more differentially expressed mRNA-coding genes than Males,^170,171^ though this was reversed when examining lncRNA (long noncoding RNA).^172^ Here we found Females had more differentially expressed genes in both ICH locations than their male counterparts. Many T Cell related pathways, T Cell and T Cell Receptor and Signaling-specific genes were significant and predicted suppressed in Female but not Male ICH. Sex differences have also been described in T Cells in healthy subjects.^173^ *NRF2*, which is neuroprotective after ICH, was associated and increased in Male Lobar but not Female ICH. Proteases involved in Aβ processing were found upregulated in Male Lobar ICH along with suppression of transcription via DNA epigenetic modifications. DNA methylation is implicated in both the risk and pathophysiology of stroke.^174^ These ICH sex differences highlight the importance of including sex when assessing ICH injury mechanisms and outcomes. Additional discussion of these findings can be found in the Supplemental Manuscript.

## 5. Conclusions

We show transcriptome differences in peripheral blood of Deep and Lobar ICH patients. These differences point to both common and different immune and inflammatory responses in the two locations. Our findings emphasize the importance of including ICH location as a factor in future studies and clinical trials as well as the potential importance of considering patient sex. Additionally, these results give evidence that location-specific treatments may be appropriate to target the specific pathophysiology associated with Deep and Lobar ICH.

## 6. Limitations

The subject numbers are small, indicating the findings will need to be validated in much larger, independent cohorts. The results are based on the whole blood transcriptome, which includes transcriptomes of all peripheral blood cell types. We report some genes as being cell-type-specific. This was done based on comparing our gene lists to cell-type specific gene lists from the literature, which have been derived from isolated blood cell types in healthy individuals. It is possible that in disease, some cell-type specific genes are expressed in other cell types. Thus, single cell transcriptomic studies in ICH are needed to address the cell-specificity of expression in ICH and how it compares to controls. Additionally, some of the observed differences may be due to changes in cell numbers of specific cell subtypes^15^ though our recent study^43^ showed differential expression of genes after stroke in isolated Monocytes and Neutrophils. Additionally, the sex-specific analysis in this study provide pilot results for potential sex differences in Deep and Lobar ICH as sex is an important factor in ICH pathology and pathophysiology.^5,175-177^ Due to this limited sample size, all CAA cases in Lobar ICH were in Male subjects. These discoveries must be validated in a cohort containing more female subjects. Since clinical severity, hematoma volumes and edema volumes were not controlled for in this study, and since they can affect gene expression,^44^ they need to be considered in future studies. Future studies will need to compare Deep ICH only associated with hypertension to Lobar ICH only associated with CAA to better clarify the differences due to these different ICH causes, rather than just considering location as in this study.

## Supplementary Material

1

2

3

4

## Figures and Tables

**Table 1:** Subject Demographics.

Demographics	Vascular Risk Factor Controls	Deep Intracerebral Hemorrhage^a^	Lobar Intracerebral Hemorrhage^b^
Subjects (#)	31	19	9
Sex (M, F), # (%)	22 (71%), 9 (29%)	15 (79%), 4 (21%)	6 (67%), 3 (33%)
Diabetes, # (%)	5 (16%)	2 (11%)	1 (11%)
Hypertension, # (%)	21 (68%)	14 (74%)	5 (56%)
Hypercholesterolemia, # (%)	11 (35%)	2 (11%)	3 (33%)
Race, # (%)			
Asian	6 (19%)	0 (0%)	2 (22%)
Black/African American	1 (3%)	3 (16%)	2 (22%)
White	18 (58%)	9 (47%)	2 (22%)
Other/Unknown	6 (19%)	7 (37%)	3 (33%)
Age (years, Mean ± SD)	62.2 ± 12.2	56.3 ± 12.8	68.2 ± 11.8
Min, Max	34, 85.3	37, 91.6	50.2, 83.8
Q1, Q2/Median, Q3	54.9, 63.3, 68.9	49.1, 55.4, 59.6	59.7, 67.6, 79.9
Time Since Event (hours, Mean ± SD)	–	50.2 ± 31.3	71.5 ± 20.8
Min, Max	–	4.2, 101.3	37.7, 98.2
Q1, Q2/Median, Q3	–	22.7, 39.6, 80.7	54.4, 73.7, 89.2
Smoking, # (%)			
Yes – Present	6 (19%)	6 (32%)	1 (11%)
Yes – Past	12 (39%)	3 (16%)	3 (33%)
Never	13 (42%)	10 (53%)	2 (22%)
Unknown	0 (0%)	0 (0%)	3 (33%)

a Hemorrhages in in the basal ganglia, thalamus, cerebellum, and pons/brainstem.

b Hemorrhages in the cortex that could extend into adjacent white matter.

**Table 2:** Hub Genes for the 2 modules significant for Deep ICH and 5 modules significant for Lobar ICH.

DC-Grey60 Hubs	DC-LightGreen Hubs	LC-Black Hubs		LC-DarkGreen Hubs	LC-Grey60 Hubs	LC-Pink Hubs		LC-RoyalBlue Hubs
AC004540.2	AC124068.1	AC022296.2	MAF1	AC021146.7	AP3S1	AC004898.1	MAPK14	AL136982.6
ACSL1	ACSL3	AC068724.4	MPP1	APC	ARMT1	AC087439.1	MCTP1	BDP1P
AL445524.2	ADAM9	AL022313.1	RNF123	BCL10	ATG3	ACSL1	MCTP2	BMS1P3
DGAT2	AGFG1	ANK1	SELENBP1	CHUK	CAPZA1	ANXA3	MGAM	BMS1P4
EGLN1	AL590002.1	AP2A1	SIAH2	CUL4B	CAPZA1P1	AP006245.2	MIR3614	CNOT6L
EGLN1P1	BNIP2	ASCC2	SLC4A1	CYB5R4	CAPZA1P2	B3GNT5	NLRC4	CNOT6LP1
ETS2	CD63	BAG6	SPTB	DCP2	CAPZA2	BCL6	PPP1R3B	ELP1
FLOT2	CLEC4D	DPM2	STRADB	FNIP1	CLIC1	C3orf86	PPP1R3D	G3BP1
GLT1D1	CNIH4	EIF1B	STRADBP1	KBTBD2	DYNLT1	CA4	PRKCD	GLS
IMPDH1P4	ERLIN1	EIF2AK1	TANGO2	PPP4R1	GMFBP1	CAMKK2	PYGL	HNRNPH1
IMPDH1P6	GYG1	EPB42	TMEM63B	RNF149	HSBP1	CCPG1	RALB	ITGA4
IMPDH1P8	GYG1P3	FAM210B	TMOD1	RRM2B	MIER1	CMTM1	STX3	KDM2B
KLHL2	HGF	FECH	TNS1	SAMD8	MOB4	DNAAF4-CCPG1	SULT1B1	MCM3
MCTP2	HK3	GATA1	TRAK2	SPOPL	MS4A6A	DOK3	TPD52L2	MRPS31P5
MGAM	HP	GLRX5P1	TRIM58	STAG2	PTPN2P2	ETS2	UBXN2B	MTR
NFIL3	HPR	GMPR	UBXN6	STX7	RNFT1	GPAT3	VENTXP2	MTX3
PFKFB4	LAMTOR5	GUCD1	VTI1B	STXBP3	SLC35A1	IFNGR2	WDFY3	NUP155
PHC2	MCEMP1	HAGH	VTI1BP1	ZFYVE16	SNX6	IL1R1	WDFY3-AS1	PDS5A
PPP1R3B	ORM1	HDGF	VTI1BP2		SNX6P1	KLHL2		SYNCRIP
PYGL	PPP1R3D	HK1			STARD3NL			TARDBP
RALGAPA2	RAB5IF				TWF1P1			TRAJ21
RNF24	SERPINB1				UBA3			
SPI1	SH3GLB1				ZFAND6			
SRPK1	SOCS3							
STX3	UGCG							
SULT1B1	WSB1							
UBXN2B								
WDFY3								
WDFY3-AS1								

## Abbreviations:

ANCOVA: Analysis of Covariance
ANOVA: Analysis of Variance
Aβ: Amyloid Beta
BBB: Blood-Brain Barrier
BH: Benjamini-Hochberg multiple test correction
CAA: Cerebral Amyloid Angiopathy
CNS: Central Nervous System
DC: Deep ICH and Control
DE: Differential Expression/Differentially Expressed
DEG: Differentially Expressed Gene
DvC: Deep ICH vs. Control
FC: Fold Change
GF: Growth Factor
GO: Gene Ontology
HC: Hierarchical Clustering
HTA: Human Transcriptome Array
ICH: Intracerebral Hemorrhage
IPA: Ingenuity Pathway Analysis
IS: Ischemic Stroke
LC: Lobar ICH and Control
LvC: Lobar ICH vs. Control
NK: Natural Killer
NKT: Natural Killer T
PCA: Principal Component Analysis
SAH: Subarachnoid Hemorrhage
SD: Standard Deviation
TBI: Traumatic Brain Injury
Th: T Helper
VRFC: Vascular Risk Factor-matched Control
WGCNA: Weighted Gene Co-expression Network Analysis

Complete abbreviations list (including gene symbols) can be found in the Supplemental Abbreviations.
