# Supplementary material for: Gene expression changes implicate specific peripheral immune responses to Deep and Lobar Intracerebral Hemorrhages in humans
Source: Brain Hemorrhages. Author manuscript; Available in PMC 2023 Mar 16. (PMC10019834; doi:10.1016/j.hest.2022.04.003)
Supplement: 1 [file NIHMS1871387-supplement-1.pdf]

Abbreviations

ABL2: ABL Proto-Oncogene 2, Non-Receptor Tyrosine Kinase
Act.: Activated
AD: Alzheimer's Disease
ALOX5: arachidonate 5-lipoxygenase
ANCOVA: Analysis of Covariance
ANOVA: Analysis of Variance
APBB1IP: amyloid beta precursor protein binding family B member 1 interacting protein
APP: amyloid beta precursor protein
AppBp1: amyloid beta precursor binding protein
APT: Array Power Tools
ARE: AU-rich elements
ATG: Autophagy-related Genes
ATG12: autophagy related 12
ATG9A: autophagy related 9A
ATGF8: (aka MAP1LC3B and LC3B) microtubule associated protein 1 light chain 3 beta
ATP: adenosine triphosphate
Aβ: Amyloid Beta
BAFF: (aka TNFSF13B) TNF superfamily member 13b
BAG6: BAG cochaperone 6
BBB: Blood-Brain Barrier
BCL10: BCL10 immune signaling adaptor
BCL-2: B-cell lymphoma 2
BCL2A1: BCL2 related protein A1
BCL2L1: BCL2 like 1
BCL2L11: (aka BIM) BCL2 like 11
BCL2L13: (aka BCL-RAMBO) BCL2 like 13
BCL6: BCL6 transcription repressor
BCL-RAMBO: (aka BCL2L13) BCL2 like 13
BEX2: brain expressed X-linked 2
BH: Benjamini-Hochberg multiple test corection
BIM: (aka BCL2L11) BCL2 like 11
BMP: Bone morphogenetic proteins
BP: Biological Process
CAA: Cerebral Amyloid Angiopathy
cAMP: Cyclic adenosine monophosphate
CASK: calcium/calmodulin dependent serine protein kinase
CASP1: caspase 1
CASP3: caspase 3
CASP4: caspase 4
CASP5: caspase 5
CC: Cellular Compartment
CCL2: C-C motif chemokine ligand 2
CCR3: C-C motif chemokine receptor 3
CCR5: C-C motif chemokine receptor 5
CCR7: C-C motif chemokine receptor 7
CD: Cluster of Differentiation
CD226: Cluster of Differentiation 226
CD28: Cluster of Differentiation 28
CD3E: Cluster of Differentiation 3 Epsilon
CD3G: Cluster of Differentiation 3 Gamma
CD4: Cluster of Differentiation 4
CD48: Cluster of Differentiation 48
CFLAR: CASP8 and FADD like apoptosis regulator
cFLIP: Cellular FLICE (FADD-like IL-1β-converting enzyme)-inhibitory protein
CHUK: component of inhibitor of nuclear factor kappa B kinase complex
CNS: Central Nervous System
CO: Carbon Monoxide
Comm.: Communication
CREB: cAMP-Response-Element Binding protein
CST3: cystatin C
CTSB: cathepsin B
CTSS: cathepsin S
DC-: Deep ICH and Control
DDX19A: DEAD-box helicase 19A
DDX21: DExD-box helicase 21
DDX5: DEAD-box helicase 5
DE: Differential Expression/Differentially Expressed
DEG: Differentially Expressed Gene
DHX29: DExH-box helicase 29
DHX36: DEAH-box helicase 36
DHX9: DExH-box helicase 9

DNAJB11: DnaJ heat shock protein family (Hsp40) member B11
DR5: (aka TNFR2 and TNFRSF1B) TNF receptor superfamily member 1B
DvC: Deep ICH vs. Control
EDN2: endothelin 2
EGLN1: (aka PHD2) egl-9 family hypoxia inducible factor 1
ELP1: elongator acetyltransferase complex subunit 1
ErbB2: erb-b2 receptor tyrosine kinase 2
ErbB3: erb-b2 receptor tyrosine kinase 3
ErbB4: erb-b2 receptor tyrosine kinase 4
EXOC3L4: exocyst complex component 3 like 4
Expr.: Expression
F13A1: coagulation factor XIII A chain
F5: Factor 5
FAK: Focal adhesion kinase
FC: Fold Change
FDR: False Discovery Rate
FGF: Fibroblast growth factor
FGF23: fibroblast growth factor 23
fMLP: N-Formylmethionyl-leucyl-phenylalanine
FNIP1: folliculin interacting protein 1
FPR1: formyl peptide receptor 1
FPR2: formyl peptide receptor 2
FTL: ferritin light chain
G3BP1: G3BP stress granule assembly factor 1
GABA: gamma-Aminobutyric acid
GABARAP: GABA type A receptor-associated protein
GABARAPL2: GABA type A receptor associated protein like 2
GCCN: GC Correction
GF: Growth Factor
GM-CSF: granulocyte-macrophage colony-stimulating factor
GO: Gene Ontology
GP6: glycoprotein VI platelet
GSN: gelsolin
HC: Hierarchical Clustering
HGF: Hepatocyte growth factor
HIF-1α: Hypoxia-inducible factor 1-alpha
HLA-DOA: major histocompatibility complex, class II, DO alpha
HMGB1: high mobility group box 1
HMOX1: (aka HO-1) heme oxygenase 1
hnRNA: heterogeneous nuclear RNA
hnRNP: Heterogeneous nuclear ribonucleoprotein
HNRNPA1: heterogeneous nuclear ribonucleoprotein A1
HNRNPH1: heterogeneous nuclear ribonucleoprotein H1
HNRNPQ: (aka SYNCRIP) synaptotagmin binding cytoplasmic RNA interacting protein
HNRNPR: heterogeneous nuclear ribonucleoprotein R
HO-1: (aka HMOX1) Heme oxygenase-1
HSF1: heat shock transcription factor 1
HSPB1: heat shock protein family B (small) member 1
HTA: Human Transcriptome Array
HTRA2: HtrA serine peptidase 2
ICH: Intracerebral Hemorrhage
IFNAR1: Interferon Alpha And Beta Receptor Subunit 1
IFNGR1: interferon gamma receptor 1
IFNGR2: interferon gamma receptor 2
IFNγ: Interferon gamma
IGF-1: Insulin-Like Growth Factor 1
IGF1R: Insulin-Like Growth Factor 1 Receptor
IKK: IκB kinase
IL: Interleukin
IL10RB: interleukin 10 receptor subunit beta
IL18: interleukin 18
IL18BP: interleukin 18 binding protein
IL18R1: interleukin 18 receptor 1
IL18RAP: interleukin 18 receptor accessory protein
IL1B: interleukin 1 beta
IL1R1: interleukin 1 receptor type 1
IL1R2: interleukin 1 receptor type 2

IL1RAP: interleukin 1 receptor accessory protein
IL1RN: interleukin 1 receptor antagonist
IL4R: interleukin 4 receptor
iNKT: Invariant natural killer T
iNOS: Inducible Nitric Oxide Signaling
IPA: Ingenuity Pathway Analysis
IS: Ischemic Stroke
ITM2B: integral membrane protein 2B
IκBα: (aka NFKBIA) NFKB inhibitor alpha
IκBζ: (aka NFKBIZ) NFKB inhibitor zeta
JAK: Janus kinase
JAK2: Janus kinase 2
JAK3: Janus kinase 3
Junct.: Junction
KIR2DL4: killer cell immunoglobulin like receptor, two Ig domains and long cytoplasmic tail 4
LC-: Lobar ICH and Control
LC3B: (aka MAP1LC3B and ATGF8) microtubule associated protein 1 light chain 3 beta
LCK: LCK proto-oncogene, Src family tyrosine kinase
lncRNA: Long Non-Coding RNA
LPS: Lipopolysaccharide
LRP1: LDL receptor related protein 1
LSD: Least Significant Difference
LvC: Lobar ICH vs. Control
LXR: Liver X Receptor
Lymph.: Lymphocyte
Mac.: Macrophages
MAP1LC3B: (aka LC3B and ATGF8) microtubule associated protein 1 light chain 3 beta
MAP3K1: mitogen-activated protein kinase kinase kinase 1
MAP3K7: (aka TAK1) mitogen-activated protein kinase kinase kinase 7
MAPK: mitogen-activated protein kinase
MAPK14: mitogen-activated protein kinase 14
MBP: myelin basic protein
MCM3: minichromosome maintenance complex component 3
MF: Molecular Function
MHC: major histocompatibility complex
MIR19A: microRNA 19a
MIR4777: microRNA 4777
MIR495: microRNA 495
MMP9: matrix metallopeptidase 9
Mon.: Monocytes
mRNA: Messenger RNA
MRPL2: Mitochondrial Ribosomal Protein L2
mRS: modified Rankin Scale
MyD88: Myeloid differentiation primary response 88
NADPH: Nicotinamide adenine dinucleotide phosphate
NEDD: Neural Precursor Cell Expressed, Developmentally Down-Regulated
NEDD4: NEDD4 E3 ubiquitin protein ligase
NEDD4L: NEDD4 like E3 ubiquitin protein ligase
NEDD8: NEDD8 ubiquitin like modifier
NFAT: Nuclear factor of activated T-cells
NFAT1: (aka NFATC2) nuclear factor of activated T cells 2
NFAT4: (NFATC3) nuclear factor of activated T cells 3
NFATC2: (aka NFAT1) nuclear factor of activated T cells 2
NFATC3: (aka NFAT4) nuclear factor of activated T cells 3
NFE2L2: (aka NRF2) NFE2 like bZIP transcription factor 2
NFKB1: Nuclear Factor Kappa B Subunit 1
NFKBIA: (aka IκBα) NFKB inhibitor alpha
NFKBIZ: (aka IκBζ) NFKB inhibitor zeta
NF-κB: Nuclear factor kappa B
NGF: Nerve growth factor
NIHSS: National Institutes of Health Stroke Scale
NK: Natural Killer
NKT: Natural Killer T

NLR: nucleotide-binding domain and leucine-rich repeat containing
NLRC3: NLR family CARD domain containing 3
NLRC4: NLR family CARD domain containing 4
NLRP12: NLR family pyrin domain containing 12
NLRP3: NLR family pyrin domain containing 3
NO: Nitric Oxide
NQO2: N-ribosyldihydronicotinamide:quinone reductase 2
NRBC: nucleated red blood cell
NRF2: (aka NFE2L2) NFE2 like bZIP transcription factor 2
NRG1: neuregulin 1
ORM1: orosomucoid 1
OSBPL8: oxysterol binding protein like 8
P2RX7: (aka P2X7R) purinergic receptor P2X 7
P2X7R: (aka P2RX7) purinergic receptor P2X 7
PAMP: Pathogen-Associated Molecular Pattern
PCA: Principal Component Analysis
PDGF: Platelet-derived growth factor
PELI1: pellino E3 ubiquitin protein ligase 1
PHD2: (aka EGLN1) egl-9 family hypoxia inducible factor 1
PI3K: phosphoinositide 3-kinase **PIK3R1:** phosphoinositide-3-kinase regulatory subunit 1
PLXNC1: plexin C1
PPAR: Peroxisome proliferator-activated receptor
PRNP: prion protein
PSEN1: presenilin 1
PSMA6: proteasome 20S subunit alpha 6
Q1: First Quartile
Q2: Second Quartile
Q3: Third Quartile
RAGE: Receptor for Advanced Glycation Endproducts
RBC: Red blood cell
Rec.: Receptor
Reg.: Regulation
RIN: RNA Integrity Number
RNP: ribonucleoprotein
RNS: Reactive Nitrogen Species
ROS: Reactive Oxygen Species
RPKM: Reads Per Kilobase Million
RSBN1: round spermatid basic protein 1
RXR: Retinoid X Receptor
RXRα: Retinoid X Receptor alpha
SAH: Subarachnoid Hemorrhage
SD: Standard Deviation
SFK: Src-Family Kinase
SIAH2: siah E3 ubiquitin protein ligase 2
siRNA: small interfering RNA
SLA: Src like adaptor
SOS2: SOS Ras/Rho guanine nucleotide exchange factor 2
SPI1: Spi-1 proto-oncogene
SPOP: speckle type BTB/POZ protein
SPOPL: speckle type BTB/POZ protein like
SPPL2A: signal peptide peptidase like 2A
SQSTM1: sequestosome 1
SST: Single Space Transformation
STAT3: signal transducer and activator of transcription 3
SUMO: Small Ubiquitin-like Modifier
SUMO1: small ubiquitin like modifier 1
SUMO1P3: SUMO1 pseudogene 3
SUMO4: small ubiquitin like modifier 4
SYNCRIP: (aka HNRNPQ) synaptotagmin binding cytoplasmic RNA interacting protein
TAB1: TGF-Beta Activated Kinase 1 (MAP3K7) Binding Protein 1
TAB2: TGF-beta activated kinase 1 (MAP3K7) binding protein 2
TAB3: TGF-beta activated kinase 1 (MAP3K7) binding protein 3
TAK1: (aka MAP3K7) mitogen-activated protein kinase kinase kinase 7
TARDBP: TAR DNA binding protein

TBI: Traumatic Brain Injury
TCR: T Cell Receptor
TGFBR1: transforming growth factor beta receptor 1
TGFBR3: transforming growth factor beta receptor 3
TGF-β: Transforming growth factor beta
Th: T Helper
TIE1: tyrosine kinase with immunoglobulin like and EGF like domains 1
TIE2: TEK receptor tyrosine kinase
TLR: Toll-Like Receptor
TLR1: toll like receptor 1
TLR10: toll like receptor 10
TLR2: Toll-Like Receptor 2
TLR4: Toll-Like Receptor 4
TLR5: toll like receptor 5
TLR6: toll like receptor 6
TLR8: toll like receptor 8
TMEM59: transmembrane protein 59
TNF: Tumor Necrosis Factor
TNFAIP6: TNF alpha induced protein 6
TNFAIP8: TNF alpha induced protein 8
TNFAIP8L2: TNF alpha induced protein 8 like 2
TNFR1: (aka TNFRSF1A) TNF receptor superfamily member 1A
TNFR2: (aka TNFRSF1B and DR5) TNF receptor superfamily member 1B
TNFRSF10B: TNF Receptor Superfamily 10b
TNFRSF10C: TNF receptor superfamily member 10c
TNFRSF1A: (aka TNFR1) TNF receptor superfamily member 1A
TNFRSF1B: (aka TNFR2 and DR5) TNF receptor superfamily member 1B
TNFSF10: (aka TRAIL) TNF Superfamily Member 10
TNFSF13B: (aka BAFF) TNF superfamily member 13b
TNF-α: Tumor necrosis factor alpha
TOB: Transducer Of ERBB2
TP53INP1: Tumor Protein P53 Inducible Nuclear Protein 1
TRAF1: TNF receptor associated factor 1
TRAF3: TNF Receptor Associated Factor 3
TRAF5: TNF receptor associated factor 5
TRAIL: (aka TNFSF10) TNF-related apoptosis-inducing ligand
TRBV29-1: T cell receptor beta variable 29-1
Treg: Regulatory T
TREM1: triggering receptor expressed on myeloid cells 1
TRK: tyrosine receptor kinase
TSP1: thrombospondin 1
TTR: Transthyretin
UBA3: ubiquitin-like modifier activating enzyme 3
UBE2I: Ubiquitin Conjugating Enzyme E2 I
UFD1: ubiquitin recognition factor in ER associated degradation 1
VEGF: Vascular Endothelial Growth Factor
VRFC: Vascular Risk Factor matched Control
WGCNA: Weighted Gene Co-expression Network Analysis
WNT: Wingless-related Integration Site
ZAP70: Zeta Chain Of T Cell Receptor Associated Protein Kinase 70
ZMPSTE24: zinc metallopeptidase STE24
