# Supplementary material for: Gene expression changes implicate specific peripheral immune responses to Deep and Lobar Intracerebral Hemorrhages in humans
Source: Brain Hemorrhages. Author manuscript; Available in PMC 2023 Mar 16. (PMC10019834; doi:10.1016/j.hest.2022.04.003)

SFigure 1.

Deep and Lobar ICH Subjects Do Not Separate By Time Since Event

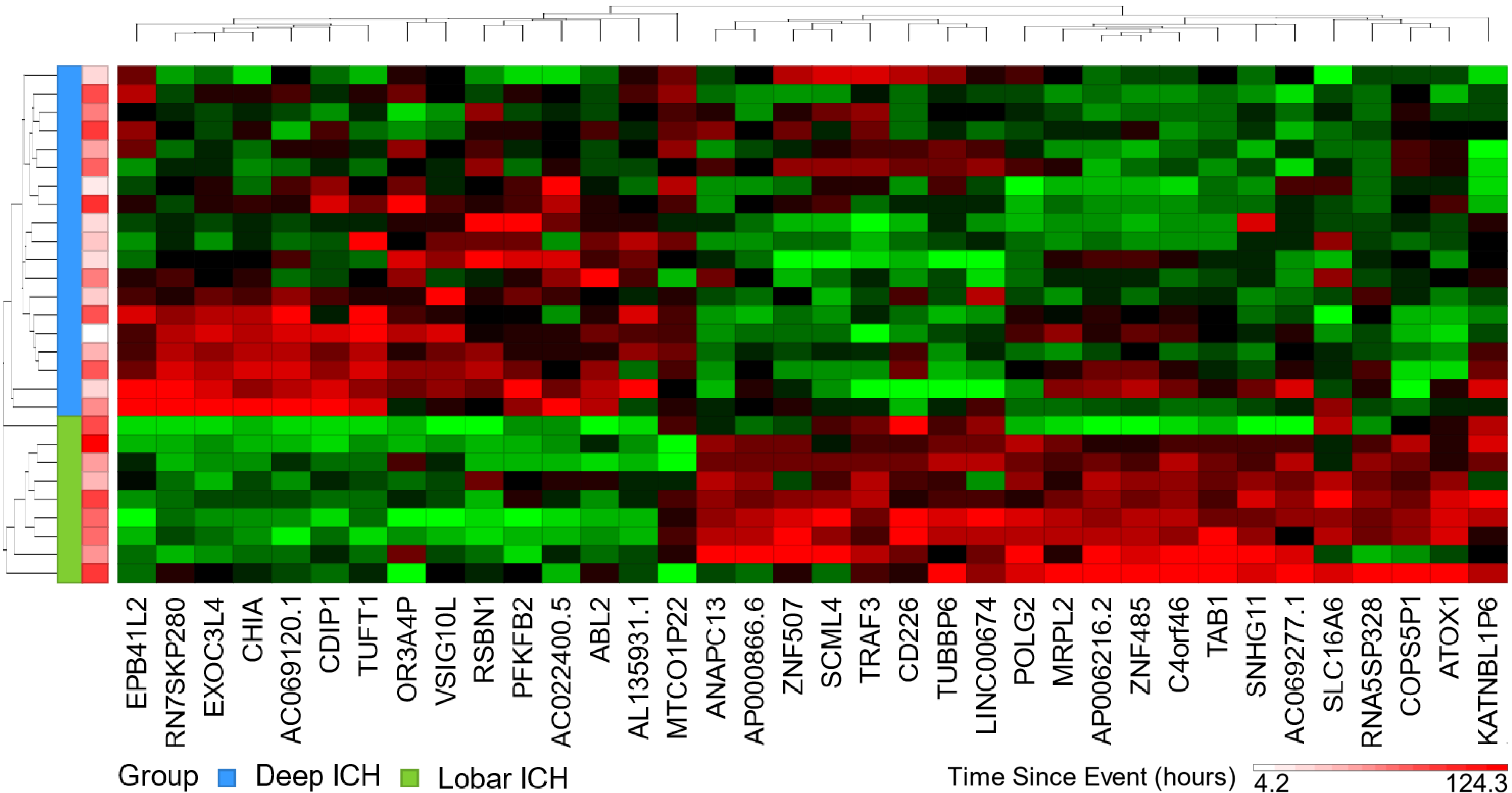

SFigure 2.

Per-Gene List Generation

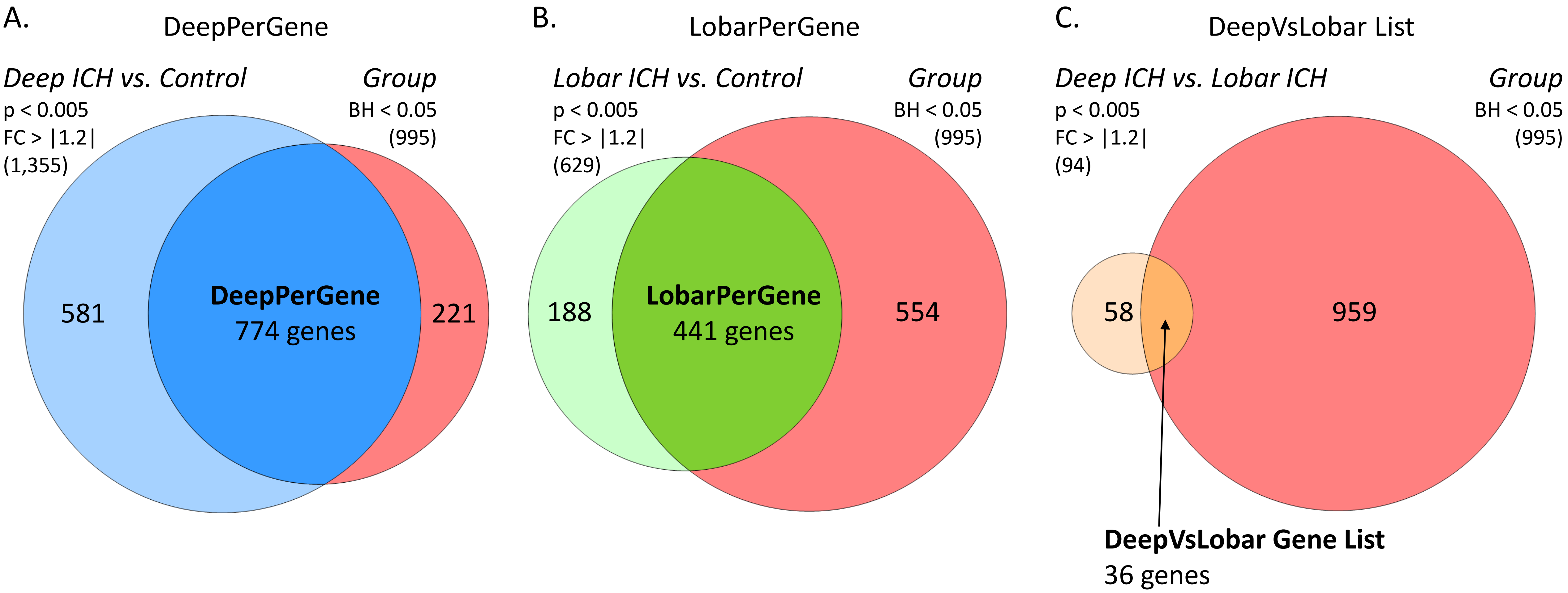

SFigure 3.

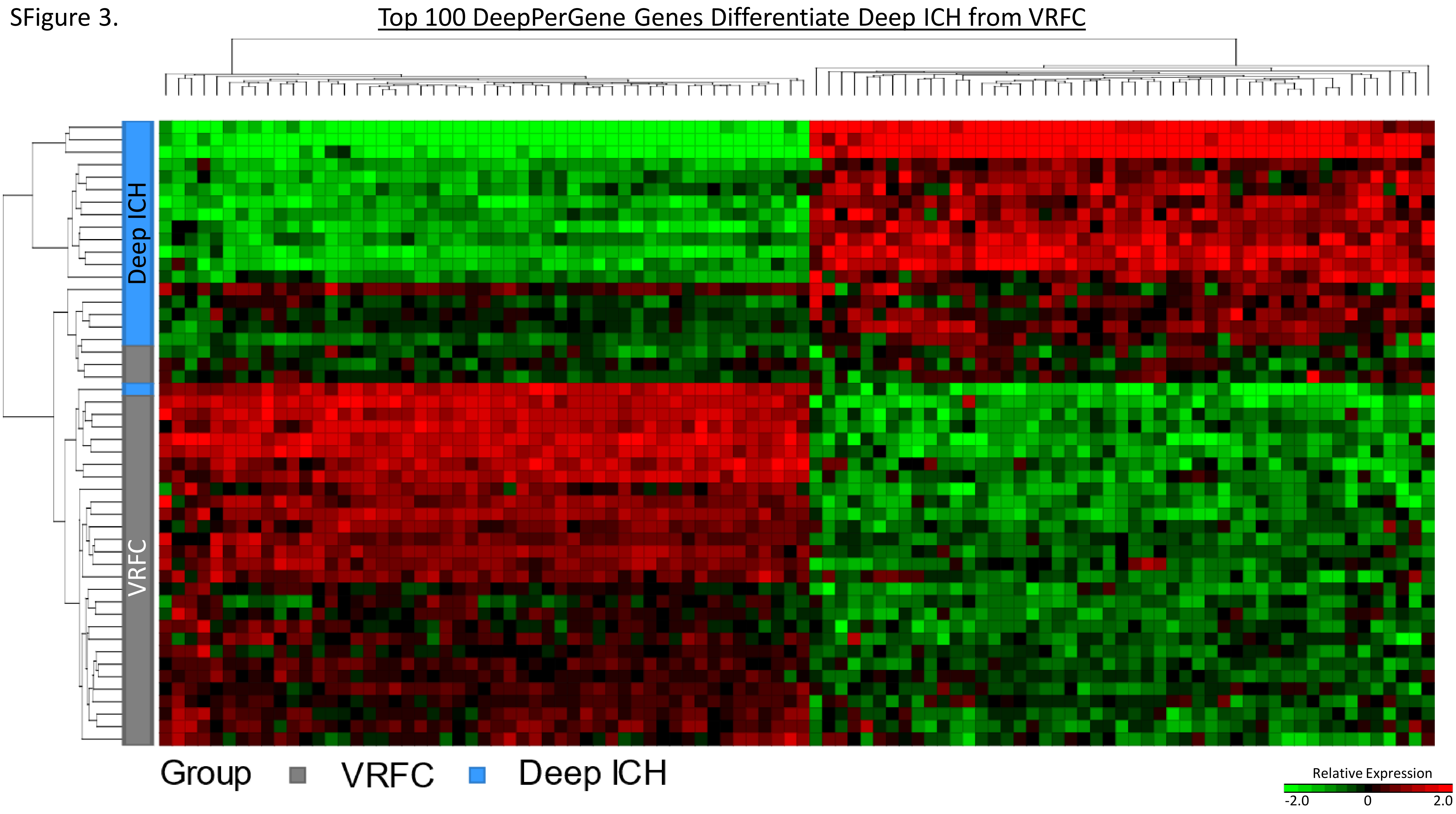
Top 100 DeepPerGene Genes Differentiate Deep ICH from VRFC
Deep ICH
VRFC
Group
VRFC
Deep ICH
Relative Expression
-2.0
0
2.0

SFigure 4.

Top 100 LobarPerGene Genes Differentiate Deep ICH from VRFC

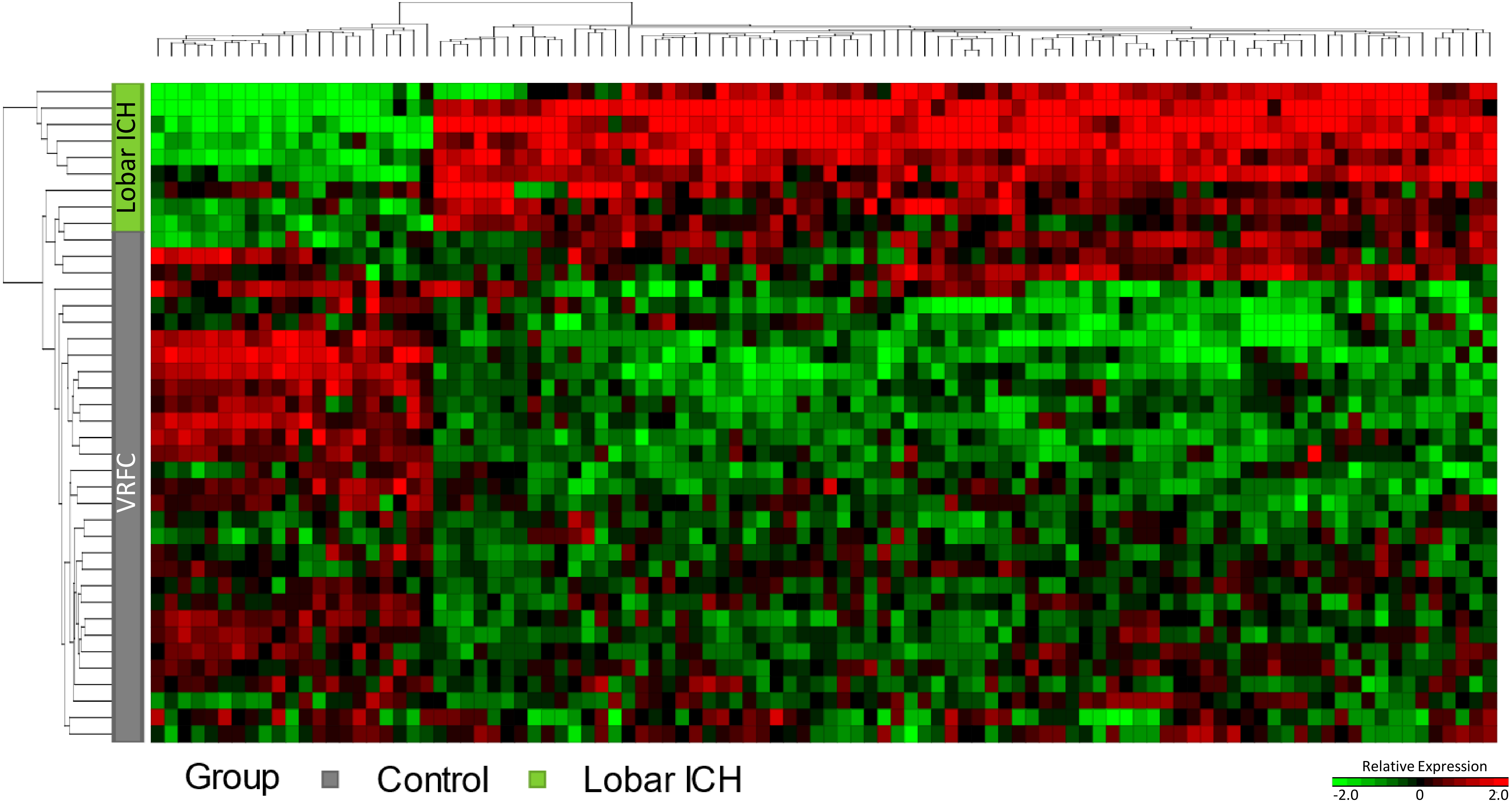

SFigure 5. T Cell Receptor Signaling Pathway with LobarPerGene Expression Predicts Activation of Inflammatory Outputs

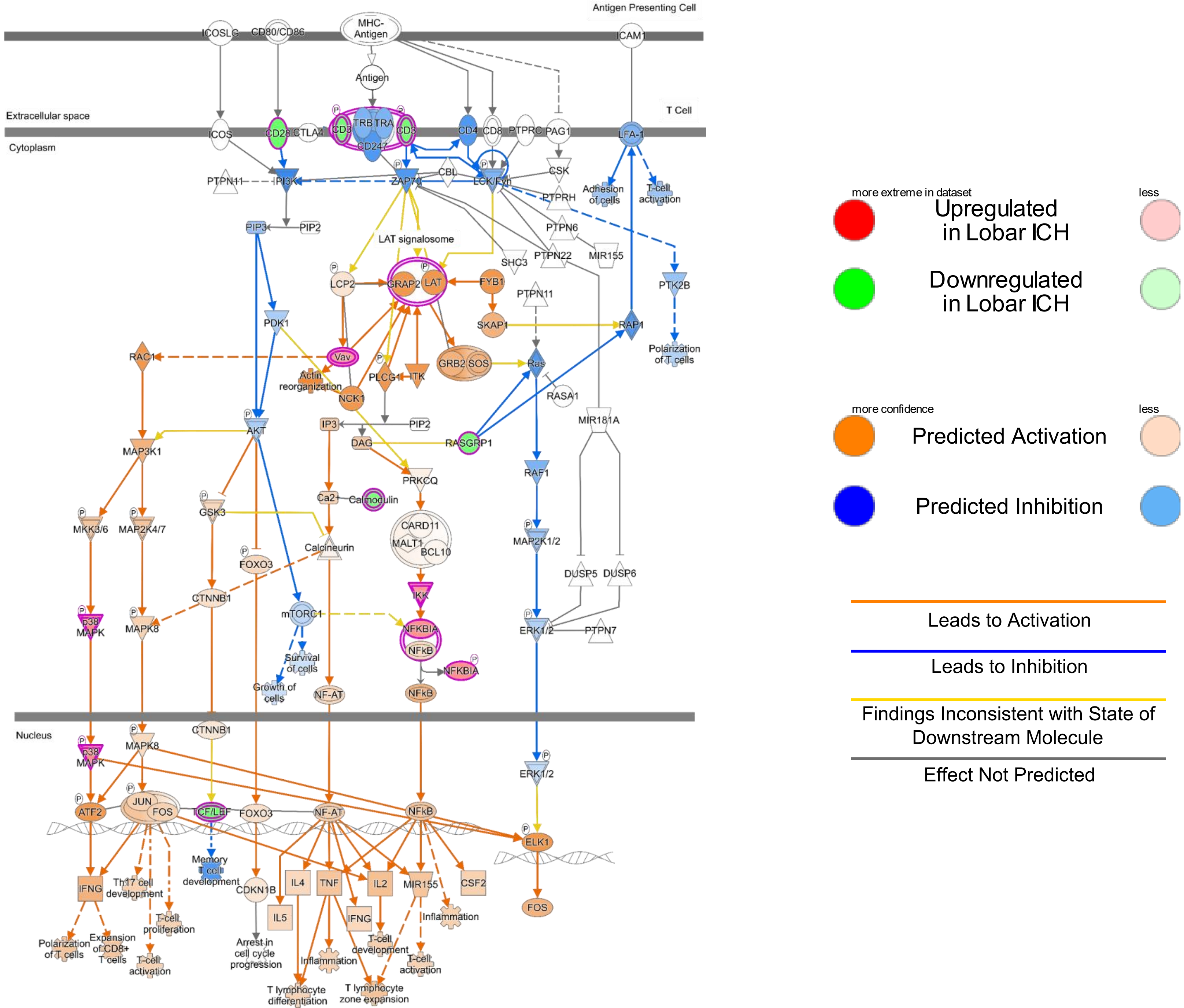

SFigure 6. DeepVsLobar Gene List Differentiates Deep and Lobar ICH from VRFC

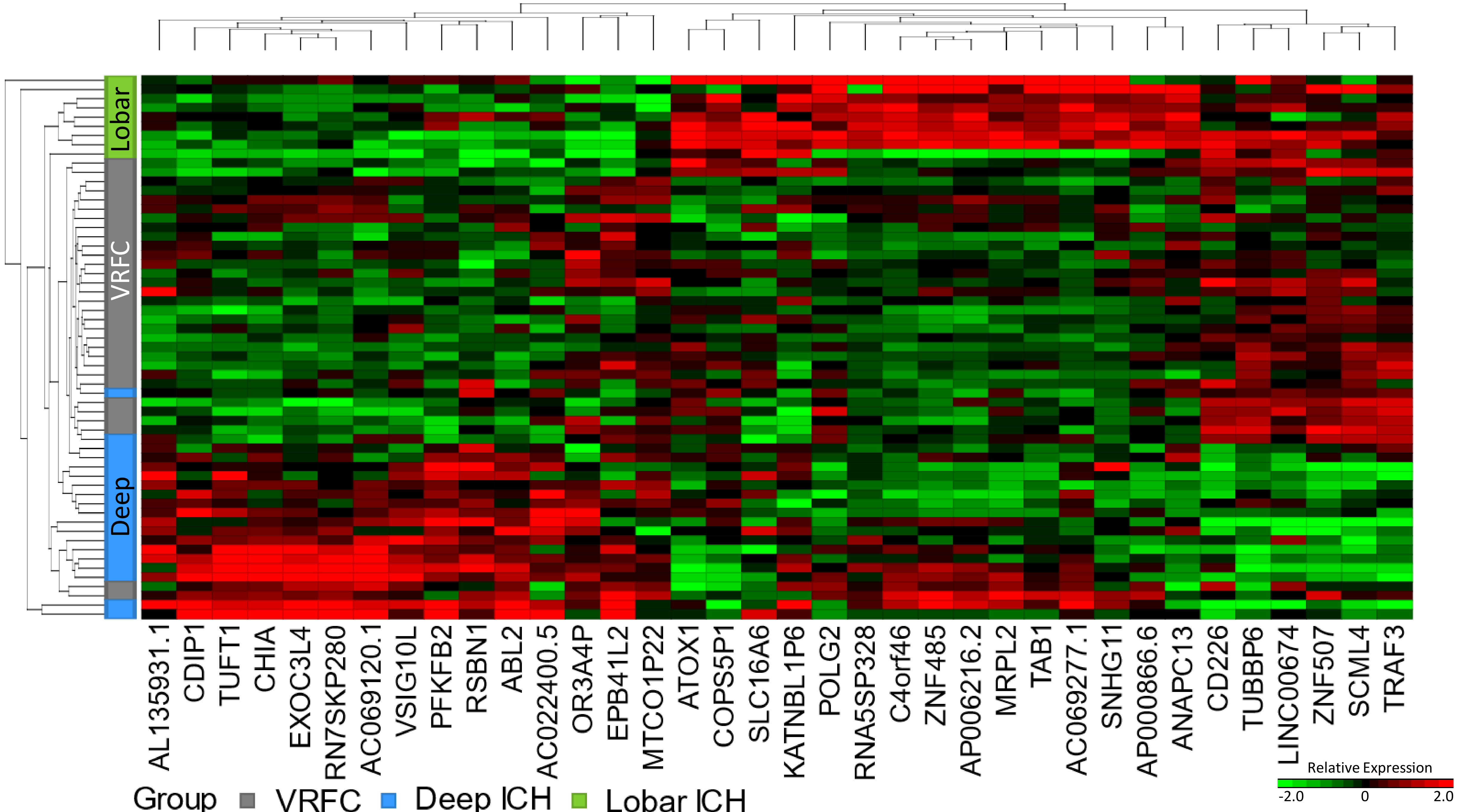

SFigure 7. Deep ICH vs Lobar ICH Genes and Their Involvement in Relevant Canonical Signaling Pathways

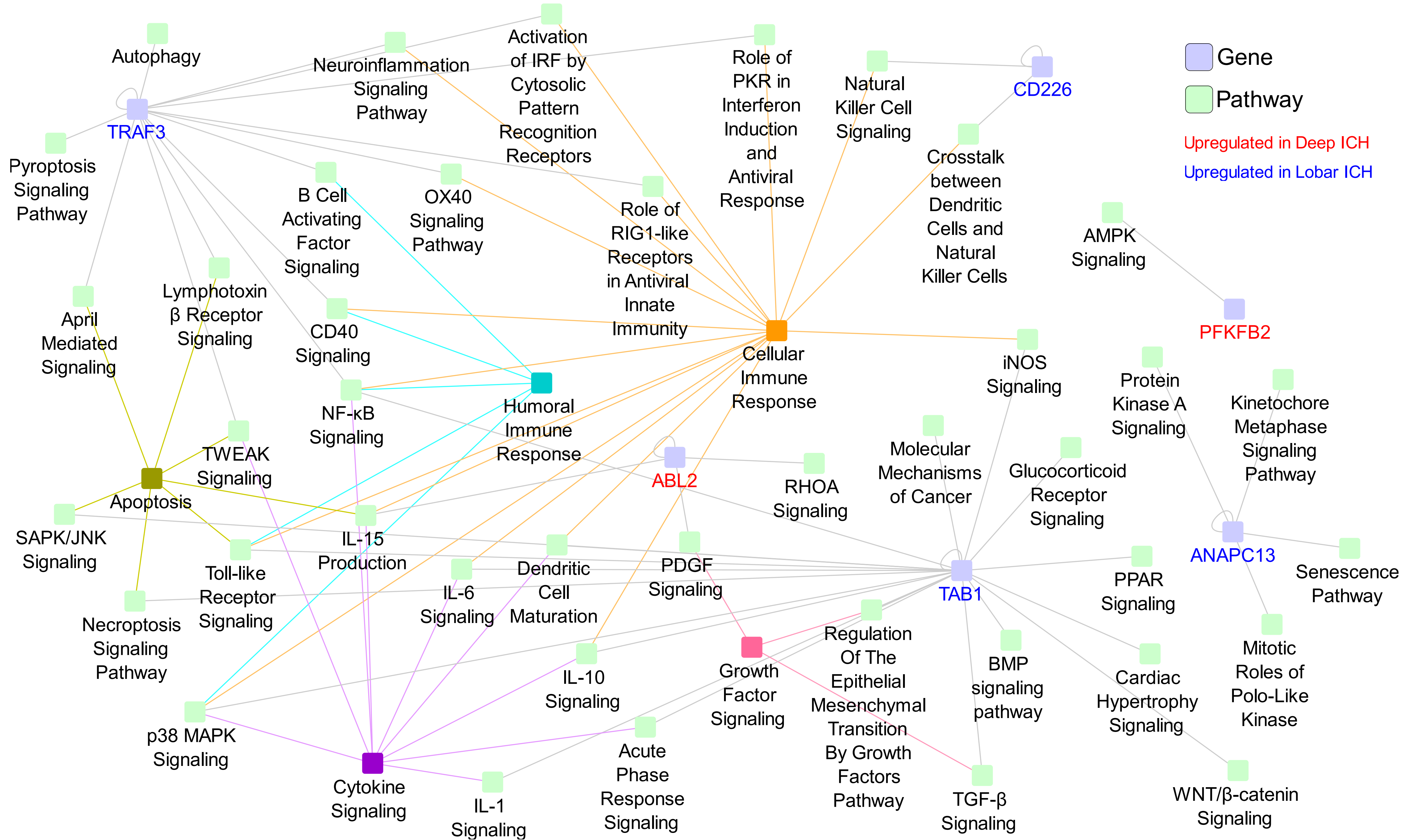

SFigure 8.

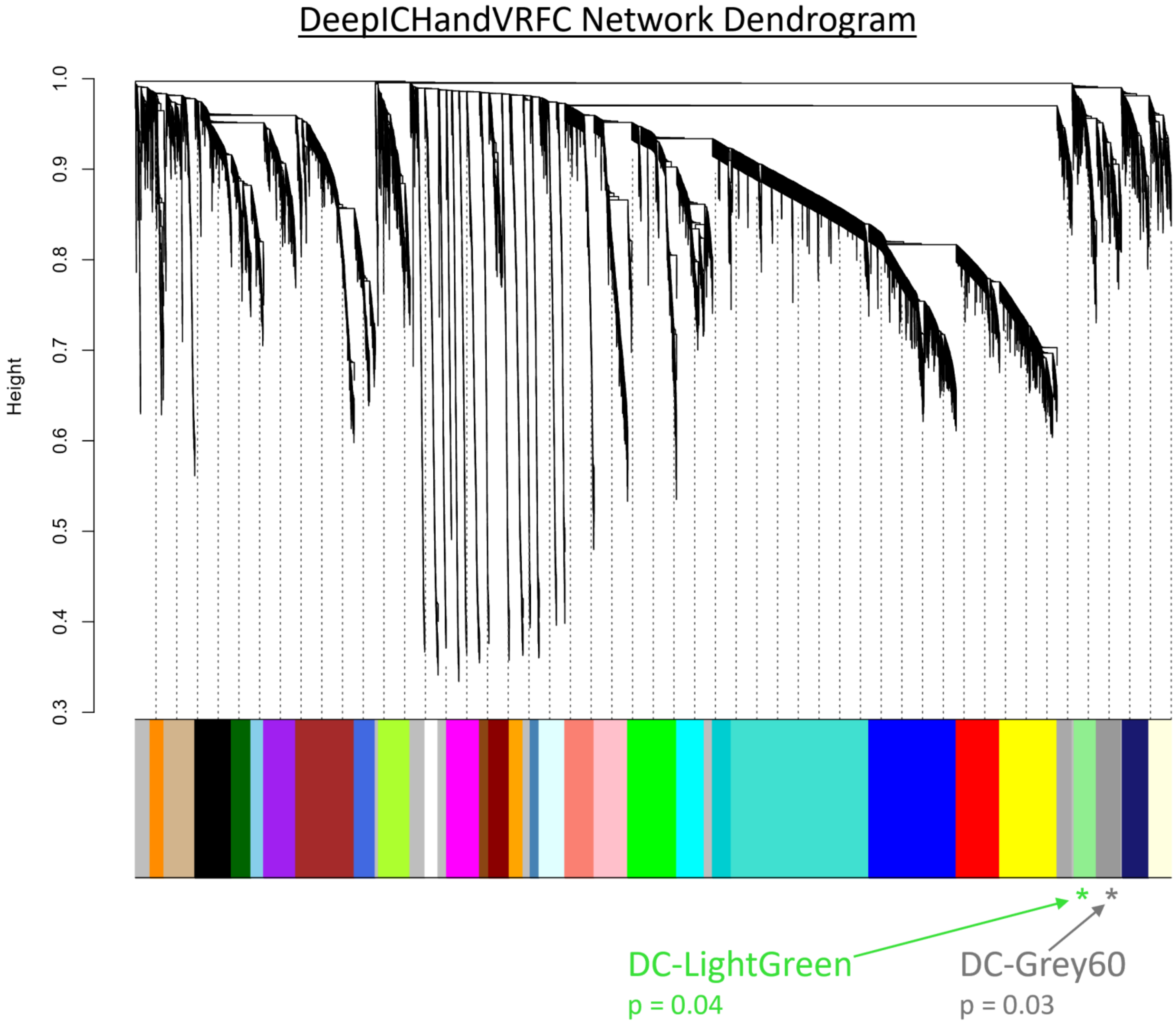

DeepICHandVRFC Network Dendrogram
Height
1.0
0.9
0.8
0.7
0.6
0.5
0.4
0.3
*
*
DC-LightGreen
p = 0.04
DC-Grey60
p = 0.03

SFigure 9. BEX2 Signaling in DC-Grey60 Implicates Increased Cell Death of Dopaminergic Neurons

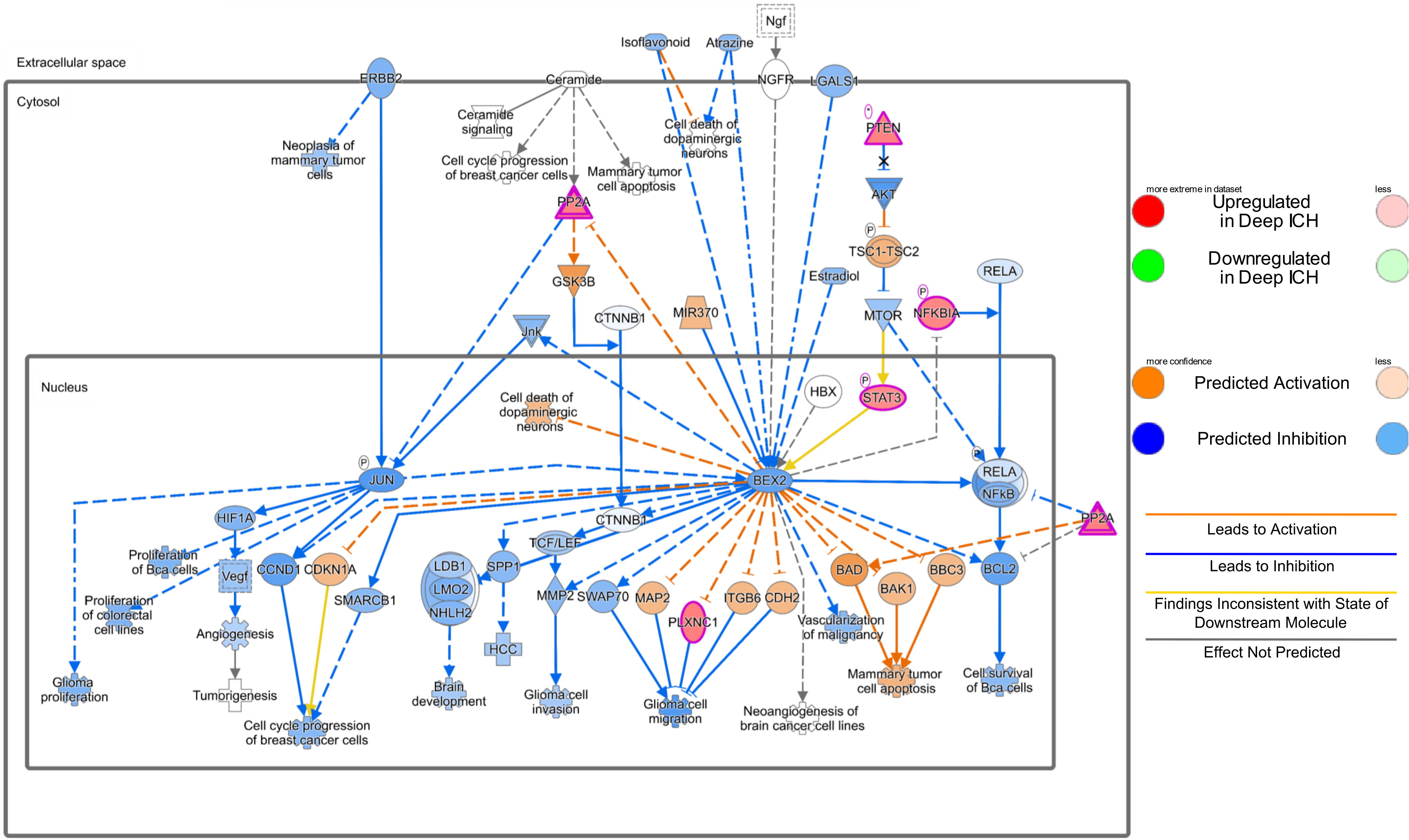

SFigure 10.

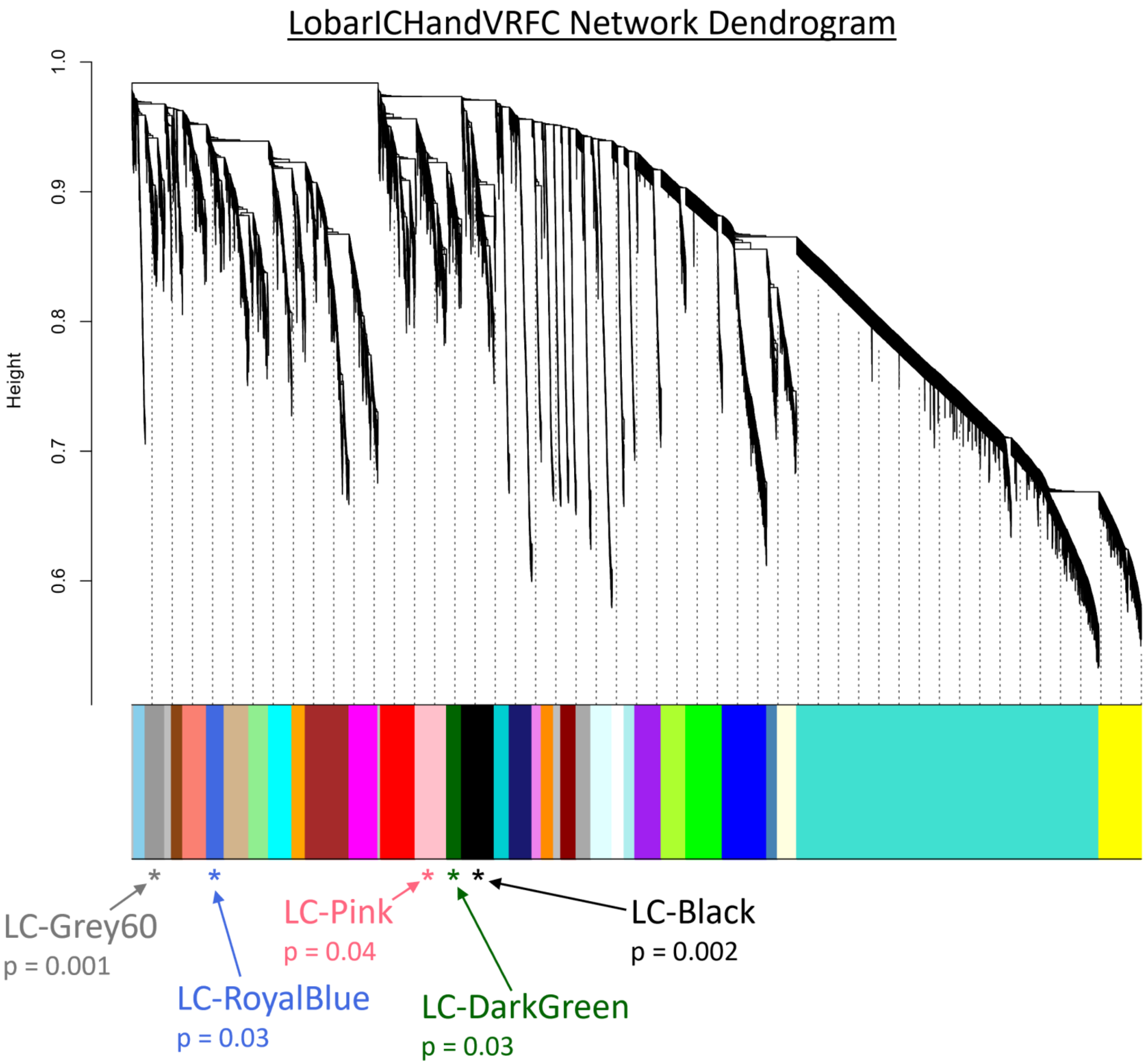
LobarICHandVRFC Network Dendrogram
Height
1.0
0.9
0.8
0.7
0.6
LC-Grey60
p = 0.001
LC-RoyalBlue
p = 0.03
LC-Pink
p = 0.04
LC-DarkGreen
p = 0.03
LC-Black
p = 0.002

SFigure 11.

Amyloid Processing is Predicted Activated in LC-Pink

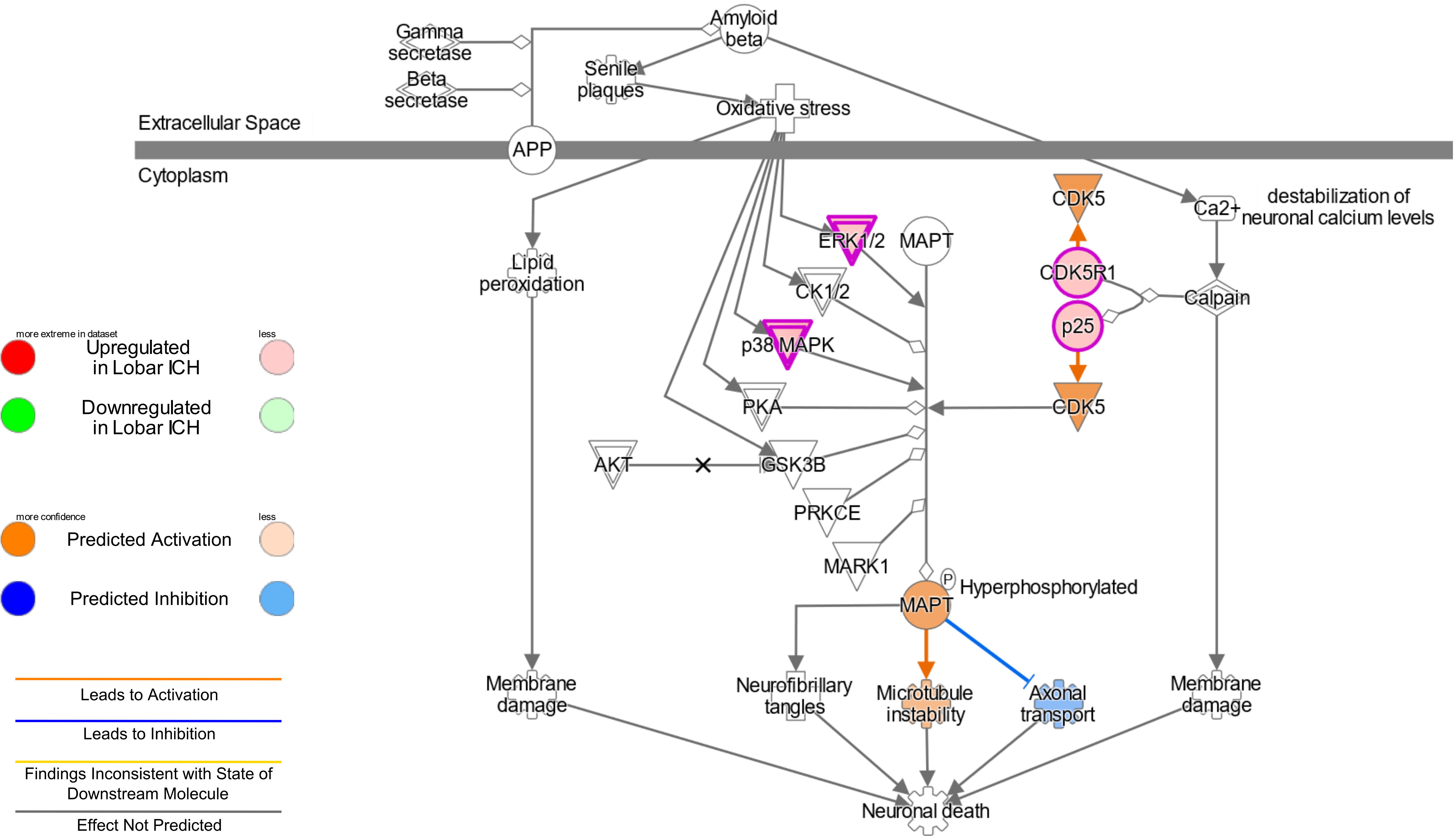

SFigure 12. Venn Diagram of Deep and Lobar ICH Significant Canonical Pathways

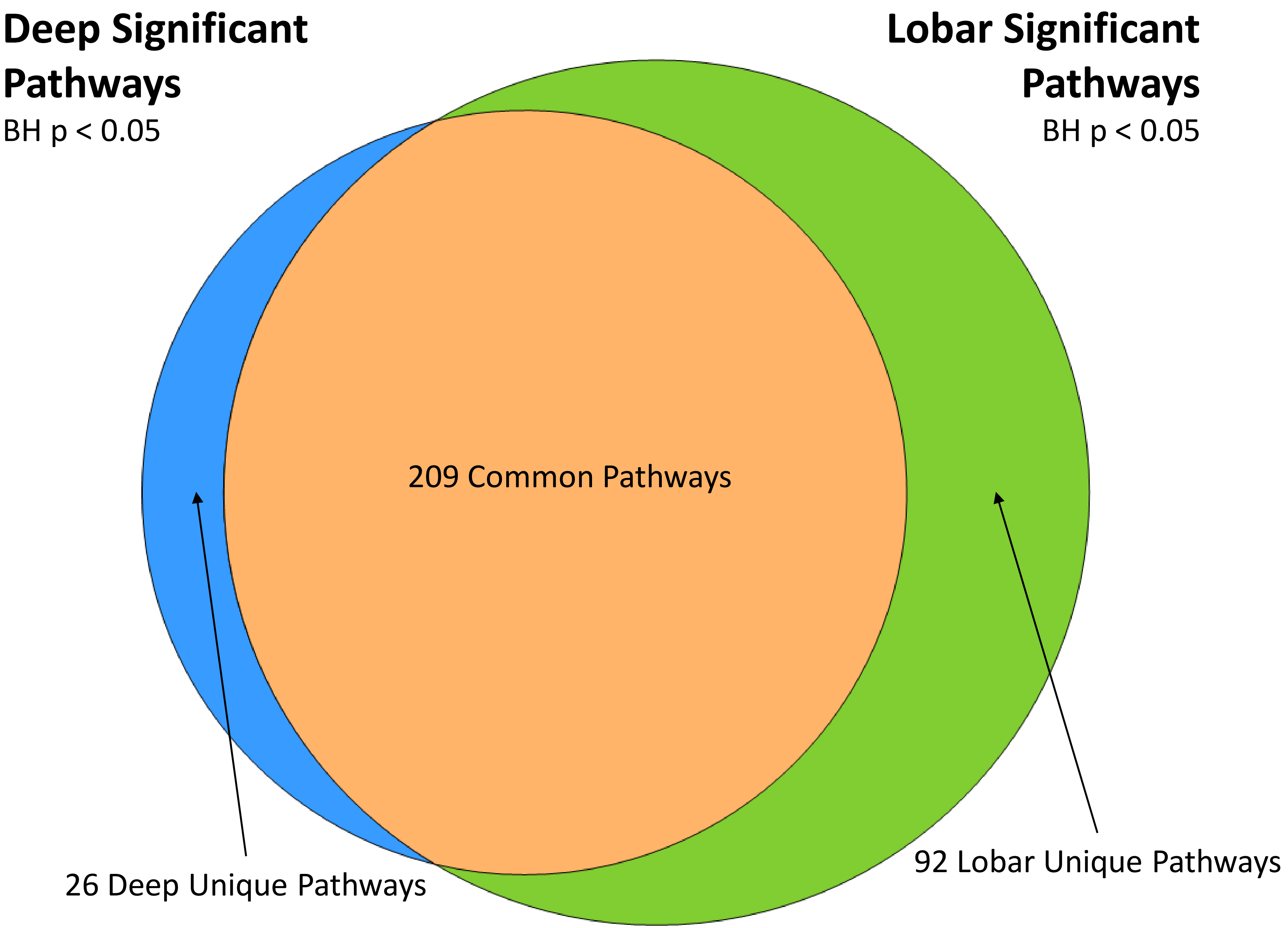

SFigure 13. Heatmap of Significant Sex-Specific Gene List Enrichment in Cell Type Specific Genes

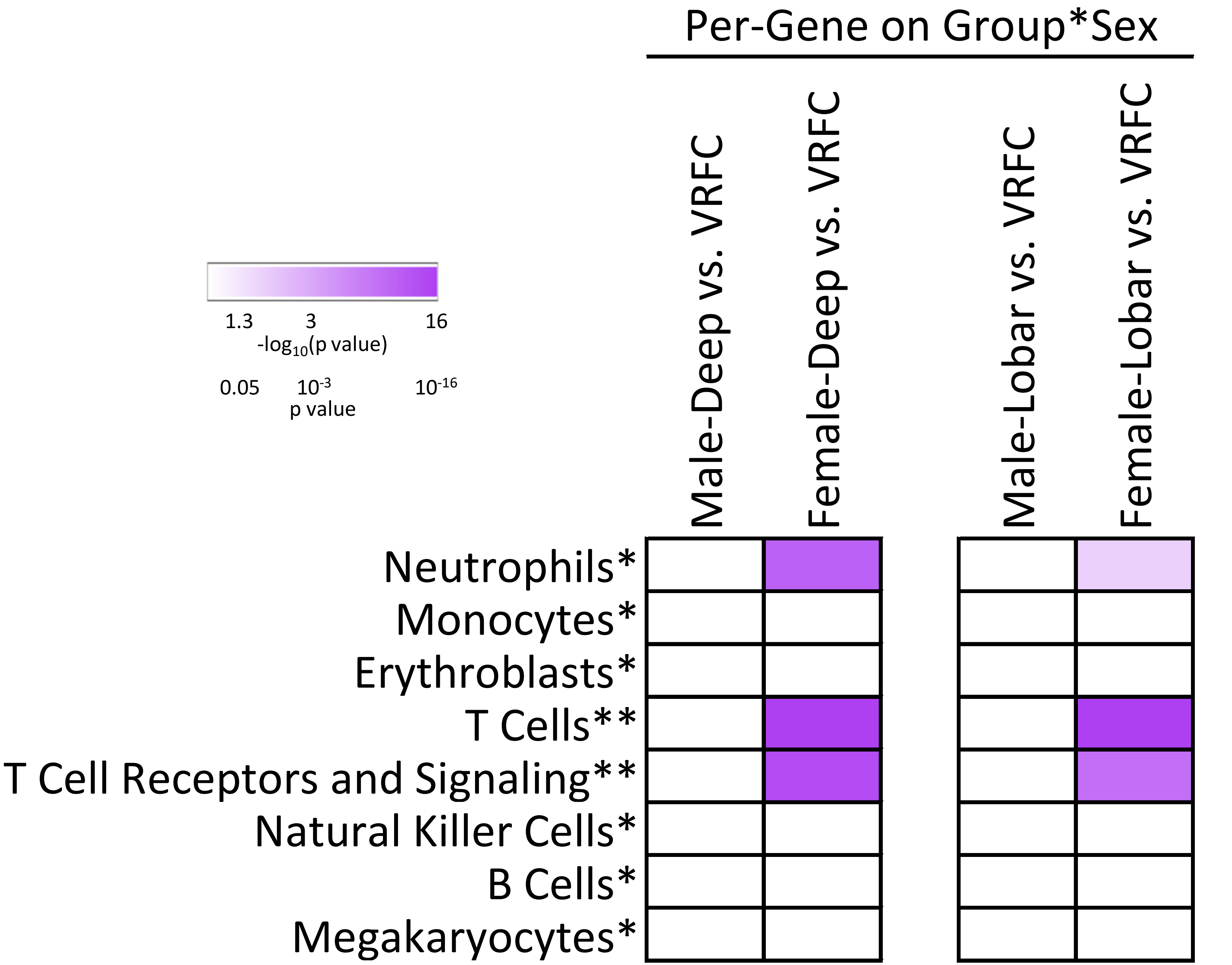

Supplement: 2 [file NIHMS1871387-supplement-2.pdf]
