# Supplementary material for: Gene expression changes implicate specific peripheral immune responses to Deep and Lobar Intracerebral Hemorrhages in humans
Source: Brain Hemorrhages. Author manuscript; Available in PMC 2023 Mar 16. (PMC10019834; doi:10.1016/j.hest.2022.04.003)
Supplement: 3 [file NIHMS1871387-supplement-3.pdf]

Supplemental Methods

Study Subjects and Arrays

The study protocol was approved by the UC Davis and UC San Francisco Institutional Review Boards and the University of Alberta Health Research Ethics Board and adheres to all federal and state regulations related to the protection of human research subjects, including The Common Rule, the principles of The Belmont Report, and Institutional policies and procedures that are founded upon ethical standards of the Declaration of Helsinki. Written informed consent was obtained from all participants or their proxy. Board-certified neurologists diagnosed ICH patients based upon medical histories, exams, and brain imaging. Subjects with Deep ICH had hemorrhages in the basal ganglia, thalamus, cerebellum, and pons/brainstem, while subjects with Lobar ICH had hemorrhages anywhere in the cortex that could extend into adjacent white matter. Blood in the adjacent ventricle may have been present in either location. Controls were matched to stroke location subgroups by vascular risk factors including age, sex, diabetes, hypertension, hyperlipidemia, and race. RNA was isolated and prepared for hybridization as previously described.[1] Quality and purity of the RNA were assessed on a Nanodrop ND-1000 spectrophotometer; RNA integrity was analyzed using the Agilent 2100 Bioanalyzer using the ratio of 28S to 18S and RNA Integrity Number (RIN). Total RNA was hybridized on GeneChip® Human Transcriptome Array (HTA) 2.0 (Affymetrix, Santa Clara, CA) to allow for examination of the coding (mRNA) and some of the non-coding human transcriptome, such as precursor (immature) microRNAs species.

Data Processing and Filtering

GC Correction (GCCN) and Single Space Transformation (SST) were applied to the HTA .CEL files using Array Power Tools (APT; Affymetrix) as we previously described.[2] GCCN-SST transformed .CEL files were then uploaded into Partek Flow software (Partek Inc, St. Louis, MO) where probe sets were mapped to the Human Genome Hg38 using the STAR 2.7.3a aligner and were processed through Partek E/M Quantify to Annotation Model. Nominal read coverage depth was defined as 30 million and default mapping parameters were used. Reads were quantified to the transcriptome (Ensembl Release 102) and RPKM normalized. Genes were then imported into Partek Genomics Suite software (PGS; Partek Inc, St. Louis, MO) for filtering based on expression when divided into Groups (Deep ICH, Lobar ICH, and VRFC). Genes were filtered out of the dataset if they did not have normalized expression values of at least 0.25 in at least 50% of samples in at least one group. This filtered out low expression genes to prevent outliers from driving false positives while retaining genes expressed in only one group. This resulted in a dataset of 23,884 genes.

Differential Expression Analysis

Log_2 (offset 0.0001) transformed expression was modeled by the ANCOVA $Y_{ijk} = \mu + Age + Group_i + Sex_j + Group*Sex_{ij} + \varepsilon_{ijk}$ where Y is the gene expression, μ is the common effect for the whole experiment; Group is Deep ICH, Lobar ICH, or VRFC; Group*Sex is the interaction between Sex and Group; and εi is the random error. Contrasts were calculated with Fisher's Least Significant Difference (LSD).[3]

ICH Locations

The Lobar ICH vs. VRFC gene list (hereafter referred to as LobarPerGene) was defined as the intersection of the Group-significant genes (Benjamini Hochberg False Discovery Rate (BH) multiple test corrected $p < 0.05$) and the Lobar ICH vs. VRFC contrast significant genes ($p < 0.005$ and Fold-Change > |1.2|). The Deep ICH vs. VRFC gene list (hereafter referred to as DeepPerGene) was defined as the intersection of the Group-significant genes (BH corrected $p < 0.05$) and the Deep ICH vs. VRFC contrast significant genes ($p < 0.005$ and Fold-Change > |1.2|). The Deep ICH vs. Lobar ICH gene list (hereafter referred to as DeepVsLobar) was defined as the intersection of the Group-significant genes (BH corrected $p < 0.05$) and the Deep ICH vs. Lobar ICH significant genes ($p < 0.005$ and Fold-Change > |1.2|).

ICH Location Sex Differences

Sex specific gene lists were selected using modified criteria due to the smaller sample size. Contrasts for the Sex*Group interaction were calculated. These contrasts included Deep ICH Males vs. VRFC Males, Deep ICH Females vs. VRFC Females, Lobar ICH Males vs. VRFC Males, and Lobar ICH Females vs. VRFC Females. Significant differences were defined based upon a $p < 0.005$ and Fold-Change > |1.2| for a given contrast. The gene lists were then compared to the opposite sex for a given location comparison: Deep ICH Males vs. VRFC Males compared to Deep ICH Females vs. VRFC Females; Lobar ICH Males vs. VRFC Males compared to Lobar ICH Females vs. VRFC Females. Genes unique to a specific contrast in these comparisons were selected for downstream biological analysis.

Weighted Gene Co-Expression Network Construction and Analysis

The function *goodSamplesGenes* within the Weighted Gene Co-Expression Network Analysis (WGCNA) package was used to identify missing values or zero-variance genes in the datasets in R.[4] Both datasets depicted an approximate scale-free topology, as is expected of gene co-expression networks.[5] Soft-thresholding powers (β) of 14 and 8 were chosen for DeepICHandVRFC and LobarICHandVRFC networks, respectively, to maximize strong correlations between genes while minimizing weak correlations.[5] WGCNA's signed network function was used to consider both positive and negative correlations.[6] Due to the large number of genes being processed, the minimum module size was set at 200 genes. The *cutreeDynamic* function was used instead of a static cutoff to form modules due to its adaptability to identify complex dendrograms that allows for identifying nested modules.[7] Hub genes were defined as

the top 5% of genes per module based on their interconnectivity (kIN—the gene's intramodular connectivity). These genes represent potential master regulators within their modules.[8,9] Module associations with Age, Diabetes, Hypertension, Hypercholesterolemia, Sex, and Group were examined independently ($p < 0.05$).

Cell-Specific Gene Involvement

Enrichment with blood cell type-specific genes was identified by overlapping our gene lists and modules with lists of blood cell type-specific genes.[10,11] Chtanova et al. was used for more comprehensive coverage of T Cell specific genes. Significance of the overlap was quantified using hypergeometric probability testing R function *phyper* with $p < 0.05$ considered significant.

Pathway and Gene Ontology Analyses

Benjamini-Hochberg (BH) multiple-comparison adjusted $p < 0.05$ was selected as a significance threshold for both Canonical Pathways and Disease and Function terms generated by IPA. IPA also determined if the significant pathways were activated or inhibited based on the expression direction (fold change) of the input genes using its pathway activity prediction analysis tool. IPA predicts activation/inhibition states of canonical pathways and disease and function terms using its Z-score algorithm, which compared our uploaded datasets and their associated fold changes with the IPA knowledge base's collection of expression patterns.[12] IPA considers the activation state of key molecules when the Pathway is activated as well as the molecules' causal relationships with each other and with functional outcomes to calculate the overall prediction. It also generates an activity pattern for the molecules and the end-point functions in the pathway. Canonical pathways with $Z \geq 2$ are predicted significantly activated (referenced hereafter as activated), while ones with $Z \leq -2$ are predicted significantly suppressed (referenced hereafter as suppressed). Similarly, IPA identifies upstream transcriptional regulators that could drive the observed expression patterns in the dataset by searching for enrichment in known targets of the regulators. A Z-score is also calculated for molecules identified as upstream regulators. Molecules with $Z \geq 2$ are considered significantly activated, while ones with $Z \leq -2$ are considered significantly suppressed.[13] For gene level analyses, ANCOVA model fold changes on Group contrasts (Deep ICH vs. VRFC; Lobar ICH vs. VRFC) were input for activity prediction. For network level analyses, fold changes were calculated with a simple ANOVA on Group with a contrast specific to the desired comparison (Deep ICH vs. VRFC; Lobar ICH vs. VRFC) for all genes within the significant modules.

We used DAVID Functional Annotation Bioinformatics Resources Database to identify enrichment with relevant biological processes (BP), cellular compartments (CC), and molecular functions (MF) with a significance threshold of EASE-adjusted BH $p < 0.05$.[14,15]

Network Visualization in Cytoscape

The *visantPrepOverall* function was used to generate a list of intramodular gene connections to visualize the module's networks.[16,17] Parameters numint=10,000 and signed=TRUE were selected. Cytoscape was then used to visualize the networks.[18,19] For each network, the minimum weight cutoff was adjusted to ensure the number of nodes and connections were visually distinguishable. Nodes representing cell-type specific genes were colored based on their cell-type and were given labels. Nodes representing hub genes were made larger and given large labels. Edge lengths were arbitrary.

Supplemental Discussion

Due to the length of the discussion in the main manuscript, we present additional discussion of this study's findings in this Supplement.

Blood Cell Response to ICH

Dendritic Cells in Deep and Lobar ICH

Dendritic cells are antigen presenting cells forming the interface between the innate and adaptive immune systems. They degrade proteins into peptides and present them as antigens to T and B Cells, and produce cytokines for T Cell activation.[20] In an ICH mouse model, they infiltrated the brain 12 hours after hemorrhage onset.[21] In this study, Dendritic Cell Maturation was a common significant pathway to both ICH locations, such as in DC-Grey60 (activated), DeepPerGene, LC-RoyalBlue (suppressed), LC-DarkGreen (activated), and LobarPerGene (activated). DeepVsLobar's member *TAB1* is involved in TNF signaling in dendritic cells, where it activates MAPK14[22] and increases expression of various surface receptors and CD proteins. *MAPK14*, which was also present in our data in the DeepPerGene list and in the LobarPerGenelist, plays a role in regulation of pro- and anti-inflammatory signaling in myeloid cells,[23] potentially through Toll-Like Receptor (TLR) regulation.[24] Six TLRs were common between ICH locations; *TLR2* and *TLR4* (involved in Dendritic Cell Maturation) had positive fold changes. In Dendritic Cells, TLRs induce pro-inflammatory cytokine production and T Cell stimulation.[25] DeepPerGene, LC-RoyalBlue and LobarPerGene were all significant for the Communication between Innate and Adaptive Immune Cells pathway, which includes Dendritic Cell stimulation of B and T Cells. In LC-RoyalBlue, *CCR7*, *HLA-DOA* (an MHC II subunit), *PIK3R1*, and *ELP1* (an IKK subunit) all had decreased expression in ICH patients. CCR7 is involved in dendritic cell migration[26] and MHC complexes are involved in antigen presentation.[27] Though both Deep and Lobar ICH had modules where Dendritic Cell Maturation was activated, the additional module in Lobar ICH (LC-RoyalBlue) where Dendritic Cell Maturation was suppressed underscores potential differences in this pathway between ICH locations.

Natural Killer and Natural Killer T Cell Response

Natural Killer T (NKT) Cells are a subset of T Lymphocytes that express TCR and NK Cell surface markers.[28,29] They respond to glycolipids like LPS, producing inflammatory cytokines and influencing the response of other immune cells.[28] Though little is known about the NKT response to ICH, studies have shown their response to ischemic stroke, potentially preventing infections.[30] DeepVsLobar genes *TRAF3* and *CD226* (both downregulated in Deep vs. Lobar ICH) are involved in many NKT functions like Frequency of iNKT1 Cells, Frequency of iNKT2 Cells, Development of Invariant Natural Killer T Cells, Proliferation of Invariant Natural Killer T Cells, and Survival of Invariant Natural Killer T Cells. This gives evidence for potential differences in the NKT response to ICH based on Location. Indeed, Deep ICH gene lists were enriched in functions Development of Natural Killer T Lymphocytes (all genes downregulated in Deep ICH vs. VRFC) and Quantity of Natural Killer T Lymphocytes (downregulated with non-significant Z = -0.89), implicating a potential downregulated NKT response to Deep hemorrhages. Lobar ICH gene lists were enriched in Apoptosis of Invariant Natural Killer T Cells and Arrest in Development of Invariant Natural Killer T Cells with gene *FNIP1* upregulated in Lobar ICH vs. VRFC. This could reduce apoptosis of iNKT cells[31] as FNIP1 is required for NKT development and survival.[31-33] Together, these results show a potential upregulation of NKT cell related pathways in Lobar ICH and a downregulation in Deep ICH. In mice, iNKT modulation (with α-galactosylceramide) was protective against infection after ischemic stroke by shifting production of cytokines from anti-inflammatory IL-10 to pro-inflammatory molecules.[34] In human ischemic stroke, increased levels of IL-10 were associated with patients with worse outcomes and infections.[35] A study of infection after human ICH found an increased risk of infection in Deep ICH compared to Lobar ICH (OR: 1.9; 95% CI: 1.28–2.88).[36] This difference may be associated with differential NKT cell activity, which may be a Deep ICH specific therapeutic target for preventing infection.

Little is known about the Natural Killer (NK) cell response to ICH. They produce cytokines and have cytotoxic functions.[37] One study showed minimal NK cell infiltration into the CNS after ICH,[38] while another showed prominent infiltration in human patients who underwent early hematoma evacuation.[39] Another group found decreased numbers of peripheral NK cells 3 and 14 days after ICH compared to controls.[40] In a previous study, we found a module of ICH associated genes enriched in NK cell specific genes.[2] In this study, DC-Grey60, DeepPerGene, LC-DarkGreen and LC-Pink were significant for Natural Killer Cell Signaling, with all but DeepPerGene activated. *CD226* (in DeepPerGene and LC-RoyalBlue) is a cell surface receptor in the Natural Killer Cell Receptor Signaling pathway. It is involved in NK cell function and regulation of dendritic cells.[41] *CD226* is also a DeepVsLobar gene, implicating different NK cell response mechanisms between ICH locations. This was reinforced by Deep but not Lobar ICH enrichment in other NK cell related functions. These included NK Cell Proliferation, Activation of Natural Killer Cells, Cytolysis of Natural Killer Cells, and Production of Natural Killer Precursor Cells. Though both locations were enriched in NK cell receptor signaling, these results show potential differences in the molecular responses of NK cells depending on hemorrhage location.

B Cell Response

The B Cell response to ICH is also poorly characterized.[42,43] B Cells are involved in cytokine production, pro- and anti-inflammatory signaling, and neuronal survival signaling, and may be involved in pre/post ischemic stroke neuroprotection.[44] There is minimal B Cell infiltration into the CNS after ICH[38] and a reduction of B Cells in peripheral blood after ICH.[40] We have previously shown peripheral differential expression of B Cell Signaling genes in ICH compared to control[1,2] and with respect to ICH and perihematomal edema volumes.[45] In this study, B Cell Receptor Signaling was significant in DC-Grey60 (activated), DeepPerGene, LC-DarkGreen (activated), LC-Pink (activated), and LC-Pink Hubs. Additionally, both Deep and Lobar ICH had modules significant for the function Quantity of B Lymphocytes, activated in DC-Grey60 and LC-Pink modules. This gives additional evidence that B Cells are potentially involved in the human ICH response in both ICH locations.

Erythroblast Response

Erythroblasts are immature nucleated red blood cells (NRBC) which mature into red blood cells (RBC).[46] They can be found in peripheral blood after large hemorrhages, inflammation, and hypoxia[47] and elevated peripheral NRBCs is associated with worse outcomes.[48] NRBCs may be able to respond to viral PAMPs (pathogen-associated molecular patterns) through TLR signaling pathways. CNS hemorrhages in preterm neonates and growth-restricted infants are also associated with elevated NRBC levels.[49,50] We have previously shown erythroblast-specific differential gene expression peripheral blood following human ICH.[2,45] In this study, LC-Black was enriched in Erythroblast cell type specific genes. Lobar ICH enrichment included Differentiation of Erythroblasts (LC-Black Hubs), Quantity of Erythroid Precursor Cells (LC-Black activated and LC-Black Hubs), Erythrocyte Differentiation (LC-Black), and Differentiation of Erythroid Cells (LC-Black and LC-DarkGreen). There was minimal involvement of Deep ICH lists in erythroid cell-related pathways, and no Deep ICH enrichment in erythroblast gene lists, pathways, functions, or GO terms. The growth factor pathway Erythropoietin Signaling was significant in DC-Grey60, DeepPerGene, and LC-DarkGreen. Erythropoietin regulates RBC generation.[46] Increased RBC generation through erythropoiesis may improve outcomes,[51] though this is debated.[52] Further studies are needed to elucidate the role of erythroblasts in ICH of different locations.

Inflammatory Signaling

Cytokine Signaling

After ICH, a large number of cytokines are released, and pro-inflammatory cytokine signaling likely contributes to secondary injury by compromising the blood-brain barrier (BBB) thus allowing for additional edema formation and immune cell invasion.[42,53] In this study, many cytokine signaling pathways were overrepresented in both Deep and Lobar ICH lists (Figure 3A), such as pro-inflammatory IL-17, IL-23, TNFR1, TNFR2, and IL-1 Signaling[53] and anti-inflammatory IL-4, IL-10, and TGF-β Signaling.[53] Activated peripheral blood cells release cytokine TNF-α after stroke,[54] beginning a pro-inflammatory signaling pathway through TNFR1

and TNFR2 in ICH.[53] *TNFRSF10B* (LC-DarkGreen) and *TNFRSF10C* (DC-Grey60 and LC-Pink) are receptors for *TNFSF10* (aka TRAIL; DC-LightGreen, DeepPerGene, and LC-GGrey60), another TNF family ligand. TRAIL signaling is implicated in neuronal apoptosis after TBI[55] and blocking TRAIL-DR5 interactions improved long term neuron survival in a mouse cerebral ischemia model.[56] *TNFSF13B* (aka BAFF), present in DC-LightGreen and LC-Grey60, is a B cell activator involved in their differentiation and replication.[57] Transcripts for 3 TNF-α induced proteins were also members of ICH-location significant modules: *TNFAIP6* in DC-LightGreen and LC-Grey60, *TNFAIP8* in LC-Grey60, and *TNFAIP8L2* in DC-LightGreen. TNF Receptor associated protein coding genes *TRAF1*, *TRAF3*, and *TRAF5* were present in Deep lists but not Lobar lists. In addition, IL-1B signaling genes were also present in the Location-significant modules for both Lobar and Deep ICH. *IL1B* was present in DC-Grey60 and LC-Pink. IL-1B is a strong pro-inflammatory mediator secreted by neurons, microglia, and astrocytes; monocytes responding to the hemorrhage may also increase IL-1B levels.[53] *IL1R1* (IL-1 Receptor 1; present in DC-Grey60 and hub in LC-Pink), *IL1R2* (IL-1 Receptor 2;DC-Grey60 and LC-Pink), *IL1RAP* (Interleukin 1 Receptor Accessory Protein; DC-Grey60 and LC-Pink), and *IL1RN* (Interleukin 1 Receptor Antagonist; DC-LightGreen) were also present. *IL18* (LC-Grey60) is another pro-inflammatory cytokine activated by the NLRP3 Inflammasome.[53] IL-18 increases in hypoxic-ischemia in rats and may contribute to brain injury.[58] Also present were *IL18BP* (Interleukin 18 Binding Protein; LC-RoyalBlue), *IL18R1* (Interleukin 18 Receptor 1; DC-LightGreen, DeepPerGene, and LC-Pink), and *IL18RAP* (Interleukin 18 Receptor Accessory Protein; DC-Grey60 and LC-Pink). Receptors for two anti-inflammatory interleukins were also common: *IL4R* (Interleukin 4 Receptor) was present in DC-Grey60, DeepPerGene, and LC-Pink while *IL10RB* (Interleukin 10 Receptor Subunit Beta) was present in DC-Grey60 and LobarPerGene. IL-4 inhibits the inflammatory response while contributing to differentiation into anti-inflammatory Th2 type T helper cells and M2 type microglial cells.[53] In a rat ICH model, injection of IL-4 induced a shift to M2 type response and decreased neural deficits.[59] In mice, IL-4 treatment improved recovery and hematoma resolution.[60] IL-10 also contributes to Th1/Th2 balance, inhibits TNF production in macrophages, and generally contributes to anti-inflammatory signaling.[53] IL-10 associates with outcomes and could act as a biomarker of long term outcomes for ICH patients.[61] *TGFBR1* (Transforming Growth Factor Beta Receptor 3) and *TGFBR3* (Transforming Growth Factor Beta Receptor 3) were both present in DeepPerGene. TGF-β contributes to Treg cell proliferation and brain repair.[53] Early TGF-β increases are associated with improved 90-day outcomes in human patients[62] and as such is a potential treatment target. The balance between these pro- and anti-inflammatory cytokines likely contributes to the damage-repair processes after ICH regardless of location, and modulation of this system might modulate outcomes.

Growth Factor Signaling

Growth factors (GF) play a role in brain recovery after ICH. Indeed, higher serum levels of various growth factors were associated with better mRS outcomes in human ICH patients at 3 months.[63,64] In this study, many GF signaling pathways were common to Deep and Lobar ICH. The ErbB Signaling pathway was common to both locations (DC-Grey60 activated, DeepPerGene, LC-DarkGreen activated, LC-Pink Hubs), as were sub-pathways ErbB2-ErbB3 Signaling (DC-Grey60 activated, LC-DarkGreen) and ErbB4 Signaling (DC-Grey60 activated,

DeepPerGene, LC-DarkGreen activated, and LC-Pink Hubs). In addition, in this study, Neuregulin Signaling (DC-Grey60, DeepPerGene, LC-Pink activated, and LC-DarkGreen) and Endocannabinoid Developing Neuron Pathway (DC-Grey60 activated, LC-Black suppressed, LC-DarkGreen activated, and LC-Pink activated) were also common between locations. Neuregulin-1 (aka NRG1) signaling through ErbB4 receptors protected against BBB disruption after SAH[65] and promoted recovery after TBI[66] in rodent models. NRG-1 was associated with neuroprotection in rodent models of IS[67-69] and may induce oligodendrocyte precursor proliferation to attenuate injury after ICH.[70] Though many preclinical studies have been performed to research the role of endocannabinoids as an IS therapy, their utility is still unclear.[71] In ICH, a rat model showed treatment with a cannabinoid receptor 2 agonist was able to reduce BBB breakdown.[72] Another common pathway, Erythropoietin Signaling, was significant in DC-Grey60 (activated), DeepPerGene, and LC-DarkGreen (activated). Erythropoietin promotes the differentiation and division of RBC,[73] and was neuroprotective after ICH in animal models.[74-77] FGF Signaling was significant in DeepPerGene and LC-DarkGreen (activated). Elevated FGF23 is a risk factor for ICH but not IS,[78] and protected the BBB after ICH in mouse models.[79] Growth Hormone Signaling was also common (DC-Grey60 activated, DeepPerGene, and LC-Pink). One study showed higher levels of Growth Hormone in ICH patients with worse outcomes and in those who died within 30 days.[80] Insulin-Like Growth Factor (IGF-1) Signaling was significant in DC-Grey60 (activated), DeepPerGene, and LC-DarkGreen (activated). IGF-1 is neuroprotective in rat IS[81] and mouse ICH.[82] Insulin-Like Growth Factor 1 Receptor (*IGF1R*) was an LC-Pink member and was found increased in ICH. IL-7 Signaling was significant in DC-Grey60 (activated), DeepPerGene, LC-Black (suppressed), LC-DarkGreen (activated), LC-Pink (activated) and LC-Pink Hubs. IL-7 is involved in the development, maintenance, and survival of all T Cell subtypes.[83] NGF Signaling was common to both locations (DeepPerGene; both LC-DarkGreen and LC-Pink activated). Treatment with mouse NGF improved outcomes in adult[84] and neonatal[85] human ICH subjects. Other GF pathways including GM-CSF Signaling and HGF Signaling were common between locations (*HGF* was a DC-LightGreen hub, though the module was not significant for the pathway).

Intracellular Signaling

Many intracellular signaling pathways are either regulated by or contribute to the production of cytokines in response to insults like hemorrhagic stroke. TLRs can be expressed on neural and glial cells and can activate the production of pro-inflammatory molecules.[86] Specifically, TLR4 is involved in inflammatory phagocytosis after ICH.[87] Toll-Like Receptor Signaling was found significant in DC-Grey60 (activated), DeepPerGene (activated), LC-DarkGreen (activated), LC-Pink (activated with hub MAPK14), and LobarPerGene. A total of 7 TLRs (*TLR1*,*2*,*4*,*5*,*6*,*8*,*10*) were present in our gene lists; *TLR10* was unique to LC-Pink while all others were common to Deep and Lobar ICH. TLR4 (DC-Grey60, DeepPerGene, LC-DarkGreen) responds to extracellular Danger-associated molecular patterns (DAMPs) including HMGB1, heme, thrombin, and fibrin.[42,43] ICH results in death and lysis of RBC, releasing hemoglobin (and thus heme) into the hematomal and perihematomal regions.[88] Additionally, the HMGB1 Signaling pathway was common between locations and was significant for DC-Grey60 (trend towards activation with Z = 1.67), DeepPerGene, LC-Pink (activated) and LC-Pink Hubs. These among other ICH induced signals activate TLR receptors and consequently begin

signaling cascades including p38 MAPK Signaling and NF-κB Signaling Pathways. Both MAPK and NF-κB are involved in inflammatory and immune signaling.[89] The p38 MAPK Signaling Pathway was significant in DC-Grey60, DC-LightGreen, DeepPerGene (activated), and LC-Pink (activated). The p38 MAPK pathway is involved in inflammatory cytokine production and apoptosis signaling.[90] Activation of p38 induces production of pro-inflammatory cytokines like IL-1, IL-6, and TNF-α.[90] In a rat ischemic stroke model, p38 inhibition was neuroprotective,[91] making it a potential treatment target.[92] Propagermanium reduced edema and improved function in ICH rats, potentially through CCL2-p38 MAPK inhibition.[93] *TAB1* (TGF-Beta Activated Kinase 1 (MAP3K7) Binding Protein 1) was a member of DeepVsLobar and was higher in Lobar ICH. Disrupting TAB1 involvement in p38 signaling could reduce MAPK induced pro inflammatory signaling.[94] NF-κB is a transcription factor responsible for modulation of gene expression in response to environmental stimuli.[95] It is highly expressed after ICH and regulates the expression of various inflammatory cytokines and HO-1, among other genes.[95] The NF-κB Signaling pathway was common to both locations, and was significant in DC-Grey60 (activated), DeepPerGene, LC-DarkGreen (activated), LC-Pink (activated), and LobarPerGene. *NFKB1* (Nuclear Factor Kappa B Subunit 1) was a member of LC-Pink, and NF-κB inhibitors *NFKBIA* (aka IκBα) and *NFKBIZ* (aka IκBζ) were common between locations. IκB binds to NF-κB, thus inhibiting it. Phosphorylation and ubiquitination leads to dissociation of the two and degradation of the inhibitor.[96] Slowing this degradation of inhibitors may be a potential target for treatments. A large number of small molecules are known to target the NF-κB pathway, but few have been examined in ICH.[96] One such small molecule, antioxidant ethyl pyruvate, was neuroprotective and reduced inflammation in an ICH mouse model.[97] However, more research is needed on NF-κB inhibitors in response to both Deep and Lobar ICH. The pathway Role of NFAT in Regulation of the Immune Response was also common between locations, and was significant in DC-Grey60 (activated), DeepPerGene, LC-DarkGreen (activated), LC-Pink (activated), LC-RoyalBlue, and LobarPerGene. NFAT family transcription factors regulate expression of T Cell activation and development genes in response to T Cell Receptor stimulation.[98] They modulate the expression of some of the genes targeted by the above intracellular signaling cascades. *NFATC2* (aka *NFAT1*) and *NFATC3* (AKA *NFAT4*) are DeepPerGene members implicated in the proportion of Th1 and Th2 cells[98] which could modulate damage and repair after ICH. NFAT proteins expressed in ischemic neurons may also contribute to IL-4 production, thus driving microglia to a restorative M2 phenotype.[99] As such, NFAT expression could be a therapeutic anti-inflammatory target after ICH. These intracellular signaling pathways play a significant role in immune and inflammatory responses to both Deep and Lobar hemorrhages and are a common response to ICH.

CREB in ICH

cAMP-response-element binding protein (CREB) is a transcription factor that induces the transcription of GFs, neurotransmitters, and intracellular signaling molecules. These targets are involved in neural protection and plasticity.[100] In ischemic stroke animal models, CREB potentiates functional recovery[101] and in ICH, CREB suppresses neuroinflammation.[102] In this study, the CREB Signaling in Neurons pathway was activated in Deep ICH, though two CREB-related genes were associated with Lobar ICH. Thus, CREB likely plays a role in Deep and Lobar ICH.

Blood Vessel Formation

Angiogenesis describes the expansion of the vascular system from existing vessels. Vasculogenesis describes the *de novo* creation of new vessels.[103] Angiogenesis occurs in response to ischemic stroke,[104] and rat models of ICH show evidence of cerebral angiogenesis.[105] The exact role of vessel formation after stroke is debated - some argue it can improve outcomes,[106] while others argue that angiogenesis-inducing factors are detrimental as they can also increase edema formation.[107] In this study, Deep and Lobar ICH gene lists were enriched in angiogenesis and vasculogenesis functions. These included Angiogenesis (in DC-LightGreen, DC-LightGreen Hubs, and activated in LC-Pink), Angiogenesis of Brain (in DC-Grey60 Hubs and DC-LightGreen Hubs), and Vasculogenesis (in DC-LightGreen and DC-LightGreen Hubs). LC-Pink was also significantly enriched in Inhibition of Angiogenesis by TSP1. The Angiopoietin Signaling Pathway was common between locations, with DeepPerGene and LC-Pink significantly enriched. Angiopoietins are ligands for TIE1 and TIE2, vascular endothelial cell tyrosine kinase receptors involved in angiogenesis.[108] Similarly, other signaling pathways common between locations are implicated in angiogenesis, some of which were activated. These include HGF Signaling,[109] HIF1α Signaling,[110,111] FAK Signaling,[112] Apelin Endothelial Signaling Pathway,[113] and PDGF Signaling.[114] The role of these pathways in the recovery from ICH requires further study to determine whether they would be important treatment targets.

Cell Death

Apoptosis in ICH

Cell death pathways (including apoptosis) can contribute to neurological deficits after ICH.[115-117] In this study, the function Apoptosis was common between locations and significant in DC-Grey60 (suppressed), DC-LightGreen, DeepPerGene (trend towards activation, Z = 1.67), LC-Black (activated), LC-DarkGreen, LC-Grey60 (trend towards suppression, Z = -1.78), LC-Pink (trend towards suppression, Z = -1.84), LC-RoyalBlue Hubs (trend towards activation, Z = 1.87), and LobarPerGene. LC-DarkGreen, LC-Pink, and LobarPerGene were significant for the Apoptosis Signaling Pathway. Many other apoptosis pathways and functions were present in both locations. Apoptosis induced by hemorrhagic products like ROS and thrombin is reported in ICH animal models.[116,118] Caspase 3 (*CASP3*), an LC-Grey60 member, is a neuronal apoptosis executioner.[116,119] *CASP1*, *CASP4*, and *CASP5* were common between locations and are involved in inflammatory regulation.[119] BCL-2 family genes (present in Deep and Lobar ICH lists) are regulators of intrinsic apoptosis pathways and may interact with some caspases.[116,120] They included *BCL2L11* (aka BIM; pro-apoptotic; in DC-LightGreen),[121] *BCL2L13* (aka BCL-RAMBO; pro-apoptotic; in LC-Black),[122] *BCL2L1* (anti-apoptotic; in LC-Black),[123,124] and *BCL2A1* (anti-apoptotic; in DC-LightGreen, DeepPerGene, LC-Grey60, and LobarPerGene).[125] BCL2A1 promotes the survival of specific leukocytes.[125] Other B-Cell Lymphoma genes included *BCL10* (pro-apoptotic; in DC-Grey60 and DeepPerGene and hub in LC-DarkGreen)[126-128] and *BCL6* (anti-apoptotic; in DC-Grey60 and hub in LC-Pink).[129,130] BCL10 induces apoptosis and activates the NF-κB pathway,[126-128] though its effectiveness is debated.[127] In our data, Apoptosis is

suggested to be suppressed in some, while activated in other modules/gene lists in both Deep and Lobar ICH, indicating complex regulation of apoptosis, with some common and some specific genes involved in both ICH locations.

Protein Processing Pathways Are Unique to Lobar ICH

Ubiquitin-Like Modifications

Protein SUMOylation is a reversible post-translational modification (using SUMOs, or small ubiquitin-like modifiers) that changes protein interaction surfaces, modifying protein stability and activity.[131] SUMOylation plays a role in inflammatory responses,[132,133] potentially through NF-κB and TLR signaling,[133] in T Cell activation,[132] and Treg activity.[133] SUMOylation plays a role in neuroprotection after cerebral ischemia / ischemic stroke in mice and rats,[134,135] though little is known about its role in ICH. We previously found that an ICH module was enriched in protein SUMOylation genes.[2] In this study one Lobar ICH module was significant for the SUMOylation Pathway, but none in Deep ICH. Lobar ICH associated with SUMO protein coding genes *SUMO1* and *SUMO4*. These SUMO proteins are activated by an E1 enzyme, transferred to an E2 enzyme like *UBE2I* (also associated with Lobar ICH), and attached to the final substrate / target proteins with the assistance of E3 enzymes.[136] Additionally, *SUMO1P3*, also associated with Lobar ICH, is implicated in proliferation and migration in cancer subtypes as an oncogene.[137-139] *NEDD4L*, a NEDD4 family E3 enzyme,[140] also associated with Lobar ICH. NEDD4L deletion worsens outcomes in ischemic stroke mouse models.[141] These combined results implicate alterations in SUMOylation and NEDDylation as potential Lobar ICH treatment targets.

Enrichment in RNA Processing, Trafficking, Splicing, and Degradation is Lobar ICH-Specific

Splicing dysfunction is associated with many diseases, and the minor spliceosome is implicated in stress-induced gene expression regulation.[142] We have previously shown differential alternative splicing in ICH[1,143] and have found enrichment in alternative splicing processes at the gene and network level in ICH.[2,45] Here, we found enrichment in various RNA processing, splicing, and degradation processes in Lobar but not Deep ICH, implicating potential differences based on ICH location. LC-RoyalBlue was found significantly enriched in GO terms RNA Processing, RNA Binding, ATP-dependent RNA helicase activity, poly(A) RNA Binding and function term Processing of RNA. LC-RoyalBlue Hubs were significantly enriched in functions Binding of RNA Fragment, Deadenylation of mRNA, Deadenylation of mRNA Fragment, Destabilization of mRNA, Localization of mRNA, Metabolism of RNA, Repression of mRNA, Splicing of hnRNA, and Splicing of RNA. LC-Black, a module where the eigengene was down-regulated in Lobar ICH vs. VRFC, was significantly enriched in Inhibition of ARE-Mediated mRNA Degradation Pathway. No RNA processing or splicing terms were significant for any of the Deep ICH lists. LC-RoyalBlue, another module where the eigengene was down-regulated in Lobar ICH vs. VRFC, had 8 nucleotide helicases (*MCM3* (hub), *DDX19A*, *DDX5*, *DHX9*, *G3BP1*

(hub), *DHX36*, *DDX21*, and *DHX29*). RNA Helicases are enzymes involved in trafficking mRNA between processing areas of the cell, removing them from the pool of actively translated transcripts,[144] and transcriptional regulation.[145-148] G3BP1 can trigger formation of stress granules (a form of ribonucleoprotein (RNP) granules), which are RNA-protein assemblies, in response to cellular stress.[145] DHX9 is involved not only in RNA processing and trafficking but also in DNA replication regulation.[147] DDX5 cooperates with hnRNPs to regulate splicing. It also regulates expression of genes involved in cell differentiation.[146] hnRNP family proteins work cooperatively on RNA processing and splicing functions.[149] *HNRNPH1* (LC-RoyalBlue Hub) regulates gene splicing.[150,151] Some of its target genes are involved in splicing, MAPK Signaling, and Ubiquitin Mediated Proteolysis.[151] Another LC-RoyalBlue hub *SYNCRIP* (aka *HNRNPQ*) is linked to splicing with *HNRNPR* (also an LC-RoyalBlue member).[150,152,153] LC-RoyalBlue Hub *TARDBP* is a paralog of *SYNCRIP* that plays a role in splicing.[154] It splices autophagy related genes[155] and is involved in cellular stress granule response and RNA storage.[156] LC-RoyalBlue also contained *HNRNPA1* pseudogenes (as currently annotated), *HNRNPH1* pseudogenes, *TARDBP* pseudogenes, and a *HNRNPR* pseudogene. Though little is known about pseudogene function, some can be processed into short interfering RNAs and can modulate transcription or translation through RNA interference,[157] thus forming another potential mechanism by which LC-RoyalBlue regulates RNA. Thus, Lobar ICH was found to be enriched in many RNA processing, trafficking, and splicing pathways that were not found in Deep ICH. These processes were enriched in the two modules (LC-RoyalBlue and LC-Black), where genes were downregulated in Lobar ICH vs. Control. Moreover, LC-RoyalBlue had no significant overlap with either of the two Deep ICH modules, signifying it is highly Lobar-specific module. Though it is likely splicing plays a role in Deep ICH, these results point to potential differential alternative splicing between ICH locations. Transcript- or alternative splicing-level analyses could unveil additional differences between Deep and Lobar ICH responses.

Sex Differences in Immune Response to Deep and Lobar Hemorrhages

Sex differences in the human immune and inflammatory systems are well characterized. These include TLR pathways, immunoglobulin activity, and leukocyte counts and production.[158] Male ICH subjects also have higher rates of hematoma expansion[159] and higher 90 day mortality rates[159,160] than females. Additionally, sex differences exist in peripheral blood gene expression in healthy subjects,[161] emphasizing the importance of sex as a factor in transcriptomic studies. Though we have previously shown transcriptome level sex differences in Ischemic Stroke (IS),[162-166] sex differences in the ICH transcriptome are not well characterized. To further our understanding of how sex impacts patient responses to ICH, we conducted a pilot analysis on sex differences in Deep and Lobar ICH.

Male ICH Response

Male-DvC (Male-Deep ICH vs. Male-Control) and Male-LvC (Male-Lobar ICH vs. Male-Control) lists had minimal enrichment in pathways, functions or terms and no enrichment in cell-type specific lists. Male-DvC was enriched in the Apelin Liver Signaling and DNA Methylation

and Transcriptional Repression Signaling pathways as well as functions Gene Silencing, Sprouting Angiogenesis, Stabilization of Synapse, and Activation-induced cell death of CD4+ T-lymphocytes. Male-LvC was not enriched in any pathways or functions. Of the 27 Male-DvC genes, 3 were miRNA (*MIR19A*, *MIR495*, and *MIR4777*) which were all upregulated in Deep ICH vs. VRFC. Inhibition of mature *MIR19A* was neuroprotective in preclinical IS studies[167-169] and could play a similar role after ICH. In mice, inhibition of mature *MIR495* miRNA promoted vascularization and blood flow recovery after IS.[170] Another Male-DvC member, Endothelin 2 (*EDN2*), is a pro-inflammatory chemoattractant of neutrophils and macrophages.[171] A variable-region T Cell receptor subunit, *TRBV29-1*, was also present in Male-DvC. Male-LvC was significant for the GO term Protein Binding. Male-DvC was not significant for any GO terms. Male-LvC genes *NRF2*, *DNAJB11*, *MAP3K1*, and *NQO2* are involved in the NRF2-Mediated Oxidative Stress Response pathway. No Male-DvC genes were involved in the pathway. *CD48* was present in Male-LvC. It is highly expressed in B and T cells.[172] *CD48*, *KIR2DL4*, and *MAP3K1*, involved in the NK Cell Signaling pathway, were differentially expressed in Male-LvC. Male-LvC also had 6 peptidase encoding genes upregulated, including *HTRA2*, *PSMA6*, *SPPL2A*, *TMEM59*, *UFD1*, and *ZMPSTE24*. HTRA2 cleaves APP, preventing mitochondrial accumulation and inhibiting the ability of Aβ plaques to form.[173] SPPL2A is implicated in AD risk, and cleaves proteins involved in neurodegeneration and immune system function.[174] TMEM59 is involved in APP shedding and downregulates Aβ formation by inhibiting α- and β-secretase activity on the precursor protein.[175]

Female ICH Response

Female-DvC (Female-Deep ICH vs. Female-Control) and Female-LvC (Female-Lobar ICH vs. Female-Control) lists were both highly enriched in various immune and inflammatory pathways already discussed. Both were significantly enriched in Neutrophil, T Cell, and T Cell Receptor and Signaling specific gene lists. In healthy patients, these immune cell types tend to have higher count and phagocytic ability in Females.[158] Female-DvC was enriched in GF signaling pathways ErbB4, GM-CSF, Growth Hormone, HGF, IL-7, Neuregulin, NGF, and PDGF Signaling. Female-LvC had no significant enrichment in GF signaling pathways. Common cytokine pathways included TREM1 Signaling (activated in Deep and Lobar ICH), Regulation of IL-2 Expression in Activated and Anergic T Lymphocytes (suppressed in both), T Helper Cell Differentiation, Th1 Pathway, Th2 Pathway, Th1 and Th2 Activation Pathway, Dendritic Cell Maturation, CCR3 Signaling in Eosinophils, and CCR5 Signaling in Macrophages. Pathways for IL-(1,2,3,22) Signaling, Inflammasome (activated), Chemokine Signaling, HMGB1 Signaling, and GO term Apoptotic Process were unique to Female-DvC. GM-CSF, IL-2, IL-3, and IL-7 are all involved in regulation of the adaptive immune system,[176] giving more evidence for changes in T and B Cell behavior after both Deep and Lobar ICH in Females. Also common to both locations were T Cell Receptor Signaling (suppressed in both), B Cell Receptor Signaling, Natural Killer Cell Signaling, fMLP Signaling in Neutrophils, and Leukocyte Extravasation Signaling. 4-1BB Signaling in T Lymphocytes, Endocannabinoid Cancer Inhibition Pathway, Response to Lipopolysaccharide, Positive Regulation of IL-4, and MyD88-dependent toll-like receptor signaling pathway were unique to Female-LvC. NF-κB Signaling, TLR Signaling (activated in both), Neuroinflammation Signaling (activated in both), and iNOS Signaling (activated in both) were other common pathways important for the human immune response to

ICH. These results are similar to our findings in Ischemic Stroke where Female differentially expressed genes were enriched in a number of immune and inflammatory pathways and GO terms.[162-164]

References

1. Stamova B, Ander BP, Jickling G, et al. The intracerebral hemorrhage blood transcriptome in humans differs from the ischemic stroke and vascular risk factor control blood transcriptomes. *J Cereb Blood Flow Metab*. Sep 2019;39(9):1818-1835. doi:10.1177/0271678X18769513
2. Durocher M, Ander BP, Jickling G, et al. Inflammatory, regulatory, and autophagy co-expression modules and hub genes underlie the peripheral immune response to human intracerebral hemorrhage. *J Neuroinflammation*. Mar 5 2019;16(1):56. doi:10.1186/s12974-019-1433-4
3. Tamhane AC, Dunlop DD. *Statistics and Data Analysis: From Elementary to Intermediate*. Prentice Hall; 2000.
4. Langfelder P, Horvath S. WGCNA: an R package for weighted correlation network analysis. *BMC Bioinformatics*. Dec 29 2008;9:559. doi:10.1186/1471-2105-9-559
5. Zhang B, Horvath S. A general framework for weighted gene co-expression network analysis. *Stat Appl Genet Mol Biol*. 2005;4:Article17. doi:10.2202/1544-6115.1128
6. Langfelder P. *Signed vs. Unsigned Topological Overlap Matrix Technical Report*. 2013. 12/5/2013. https://horvath.genetics.ucla.edu/html/CoexpressionNetwork/Rpackages/WGCNA/TechnicalReports/signedTOM.pdf
7. Langfelder P, Zhang B, Horvath S. Defining clusters from a hierarchical cluster tree: the Dynamic Tree Cut package for R. *Bioinformatics*. Mar 1 2008;24(5):719-20. doi:10.1093/bioinformatics/btm563
8. Yang Y, Han L, Yuan Y, Li J, Hei N, Liang H. Gene co-expression network analysis reveals common system-level properties of prognostic genes across cancer types. *Nat Commun*. 2014;5:3231. doi:10.1038/ncomms4231
9. Langfelder P, Mischel PS, Horvath S. When is hub gene selection better than standard meta-analysis? *PLoS One*. 2013;8(4):e61505. doi:10.1371/journal.pone.0061505
10. Watkins NA, Gusnanto A, de Bono B, et al. A HaemAtlas: characterizing gene expression in differentiated human blood cells. *Blood*. May 7 2009;113(19):e1-9. doi:10.1182/blood-2008-06-162958
11. Chtanova T, Newton R, Liu SM, et al. Identification of T cell-restricted genes, and signatures for different T cell responses, using a comprehensive collection of microarray datasets. *J Immunol*. Dec 15 2005;175(12):7837-47. doi:10.4049/jimmunol.175.12.7837
12. Ingenuity Downstream Effects Analysis in IPA. Accessed 2/2/22, http://pages.ingenuity.com/rs/ingenuity/images/0812%20downstream_effects_analysis_whitepaper.pdf
13. Ingenuity Upstream Regulator Analysis in IPA. Accessed 2/2/22, http://pages.ingenuity.com/rs/ingenuity/images/0812%20upstream_regulator_analysis_whitepaper.pdf
14. Huang da W, Sherman BT, Lempicki RA. Bioinformatics enrichment tools: paths toward the comprehensive functional analysis of large gene lists. *Nucleic Acids Res*. Jan 2009;37(1):1-13. doi:10.1093/nar/gkn923
15. Huang da W, Sherman BT, Lempicki RA. Systematic and integrative analysis of large gene lists using DAVID bioinformatics resources. *Nat Protoc*. 2009;4(1):44-57. doi:10.1038/nprot.2008.211
16. Miller JA, Horvath S, Geschwind DH. Divergence of human and mouse brain transcriptome highlights Alzheimer disease pathways. *Proc Natl Acad Sci U S A*. Jul 13 2010;107(28):12698-703. doi:10.1073/pnas.0914257107

17. Hu Z, Snitkin ES, DeLisi C. VisANT: an integrative framework for networks in systems biology. *Brief Bioinform*. Jul 2008;9(4):317-25. doi:10.1093/bib/bbn020
18. Shannon P, Markiel A, Ozier O, et al. Cytoscape: a software environment for integrated models of biomolecular interaction networks. *Genome Res*. Nov 2003;13(11):2498-504. doi:10.1101/gr.1239303
19. yFiles Layout Algorithms for Cytoscape. yWorks. Accessed 11/3/21, https://www.yworks.com/products/yfiles-layout-algorithms-for-cytoscape
20. Mellman I. Dendritic cells: master regulators of the immune response. *Cancer Immunol Res*. Sep 2013;1(3):145-9. doi:10.1158/2326-6066.CIR-13-0102
21. Hammond MD, Ai Y, Sansing LH. Gr1+ Macrophages and Dendritic Cells Dominate the Inflammatory Infiltrate 12 Hours After Experimental Intracerebral Hemorrhage. *Transl Stroke Res*. Jul 2012;3(1):s125-s131. doi:10.1007/s12975-012-0174-9
22. Ge B, Gram H, Di Padova F, et al. MAPKK-independent activation of p38alpha mediated by TAB1-dependent autophosphorylation of p38alpha. *Science*. Feb 15 2002;295(5558):1291-4. doi:10.1126/science.1067289
23. Kim C, Sano Y, Todorova K, et al. The kinase p38 alpha serves cell type-specific inflammatory functions in skin injury and coordinates pro- and anti-inflammatory gene expression. *Nat Immunol*. Sep 2008;9(9):1019-27. doi:10.1038/ni.1640
24. Huang G, Shi LZ, Chi H. Regulation of JNK and p38 MAPK in the immune system: signal integration, propagation and termination. *Cytokine*. Dec 2009;48(3):161-9. doi:10.1016/j.cyto.2009.08.002
25. Hemmi H, Akira S. TLR signalling and the function of dendritic cells. *Chem Immunol Allergy*. 2005;86:120-135. doi:10.1159/000086657
26. Riol-Blanco L, Sanchez-Sanchez N, Torres A, et al. The chemokine receptor CCR7 activates in dendritic cells two signaling modules that independently regulate chemotaxis and migratory speed. *J Immunol*. Apr 1 2005;174(7):4070-80. doi:10.4049/jimmunol.174.7.4070
27. Guermonprez P, Valladeau J, Zitvogel L, Thery C, Amigorena S. Antigen presentation and T cell stimulation by dendritic cells. *Annu Rev Immunol*. 2002;20:621-67. doi:10.1146/annurev.immunol.20.100301.064828
28. Wu L, Van Kaer L. Natural killer T cells in health and disease. *Front Biosci (Schol Ed)*. Jan 1 2011;3:236-51. doi:10.2741/s148
29. Kronenberg M. Toward an understanding of NKT cell biology: progress and paradoxes. *Annu Rev Immunol*. 2005;23:877-900. doi:10.1146/annurev.immunol.23.021704.115742
30. Cui Y, Wan Q. NKT Cells in Neurological Diseases. *Front Cell Neurosci*. 2019;13:245. doi:10.3389/fncel.2019.00245
31. Park H, Tsang M, Iritani BM, Bevan MJ. Metabolic regulator Fnip1 is crucial for iNKT lymphocyte development. *Proc Natl Acad Sci U S A*. May 13 2014;111(19):7066-71. doi:10.1073/pnas.1406473111
32. Pei B, Zhao M, Miller BC, et al. Invariant NKT cells require autophagy to coordinate proliferation and survival signals during differentiation. *J Immunol*. Jun 15 2015;194(12):5872-84. doi:10.4049/jimmunol.1402154
33. Yang G, Driver JP, Van Kaer L. The Role of Autophagy in iNKT Cell Development. *Front Immunol*. 2018;9:2653. doi:10.3389/fimmu.2018.02653
34. Wong CH, Jenne CN, Lee WY, Leger C, Kubes P. Functional innervation of hepatic iNKT cells is immunosuppressive following stroke. *Science*. Oct 7 2011;334(6052):101-5. doi:10.1126/science.1210301
35. Wong CH, Jenne CN, Tam PP, et al. Prolonged Activation of Invariant Natural Killer T Cells and TH2-Skewed Immunity in Stroke Patients. *Front Neurol*. 2017;8:6. doi:10.3389/fneur.2017.00006

36. Lord AS, Langefeld CD, Sekar P, et al. Infection after intracerebral hemorrhage: risk factors and association with outcomes in the ethnic/racial variations of intracerebral hemorrhage study. *Stroke*. Dec 2014;45(12):3535-42. doi:10.1161/STROKEAHA.114.006435
37. Vivier E, Tomasello E, Baratin M, Walzer T, Ugolini S. Functions of natural killer cells. *Nat Immunol*. May 2008;9(5):503-10. doi:10.1038/ni1582
38. Mracsko E, Javidi E, Na SY, Kahn A, Liesz A, Veltkamp R. Leukocyte invasion of the brain after experimental intracerebral hemorrhage in mice. *Stroke*. Jul 2014;45(7):2107-14. doi:10.1161/STROKEAHA.114.005801
39. Li Z, Li M, Shi SX, et al. Brain transforms natural killer cells that exacerbate brain edema after intracerebral hemorrhage. *J Exp Med*. Dec 7 2020;217(12)doi:10.1084/jem.20200213
40. Zhang J, Shi K, Li Z, et al. Organ- and cell-specific immune responses are associated with the outcomes of intracerebral hemorrhage. *FASEB J*. Jan 2018;32(1):220-229. doi:10.1096/fj.201700324R
41. Huang Z, Qi G, Miller JS, Zheng SG. CD226: An Emerging Role in Immunologic Diseases. *Front Cell Dev Biol*. 2020;8:564. doi:10.3389/fcell.2020.00564
42. Mracsko E, Veltkamp R. Neuroinflammation after intracerebral hemorrhage. *Front Cell Neurosci*. 2014;8:388. doi:10.3389/fncel.2014.00388
43. Tschoe C, Bushnell CD, Duncan PW, Alexander-Miller MA, Wolfe SQ. Neuroinflammation after Intracerebral Hemorrhage and Potential Therapeutic Targets. *J Stroke*. Jan 2020;22(1):29-46. doi:10.5853/jos.2019.02236
44. Selvaraj UM, Poinsatte K, Torres V, Ortega SB, Stowe AM. Heterogeneity of B Cell Functions in Stroke-Related Risk, Prevention, Injury, and Repair. *Neurotherapeutics*. Oct 2016;13(4):729-747. doi:10.1007/s13311-016-0460-4
45. Durocher M, Knepp B, Yee A, et al. Molecular Correlates of Hemorrhage and Edema Volumes Following Human Intracerebral Hemorrhage Implicate Inflammation, Autophagy, mRNA Splicing, and T Cell Receptor Signaling. *Transl Stroke Res*. Oct 2021;12(5):754-777. doi:10.1007/s12975-020-00869-y
46. Moras M, Lefevre SD, Ostuni MA. From Erythroblasts to Mature Red Blood Cells: Organelle Clearance in Mammals. *Front Physiol*. 2017;8:1076. doi:10.3389/fphys.2017.01076
47. Schwartz SO, Stansbury F. Significance of nucleated red blood cells in peripheral blood; analysis of 1,496 cases. *J Am Med Assoc*. Apr 17 1954;154(16):1339-40. doi:10.1001/jama.1954.02940500019007
48. Purtle SW, Horkan CM, Moromizato T, Gibbons FK, Christopher KB. Nucleated red blood cells, critical illness survivors and postdischarge outcomes: a cohort study. *Crit Care*. Jun 21 2017;21(1):154. doi:10.1186/s13054-017-1724-z
49. Steurer MA, Berger TM. Massively elevated nucleated red blood cells and cerebral or pulmonary hemorrhage in severely growth-restricted infants--is there more than coincidence? *Neonatology*. 2008;94(4):314-9. doi:10.1159/000151654
50. Zhang H, Chen M, Liu Y, et al. Paroxetine combined with fluorouracil plays a therapeutic role in mouse models of colorectal cancer with depression through inhibiting IL-22 expression to regulate the MAPK signaling pathway. *Exp Ther Med*. Dec 2020;20(6):240. doi:10.3892/etm.2020.9370
51. Sheth KN, Gilson AJ, Chang Y, et al. Packed red blood cell transfusion and decreased mortality in intracerebral hemorrhage. *Neurosurgery*. May 2011;68(5):1286-92. doi:10.1227/NEU.0b013e31820cccb2
52. Roh DJ, Carvalho Poyraz F, Magid-Bernstein J, et al. Red Blood Cell Transfusions and Outcomes After Intracerebral Hemorrhage. *J Stroke Cerebrovasc Dis*. Dec 2020;29(12):105317. doi:10.1016/j.jstrokecerebrovasdis.2020.105317
53. Zhu H, Wang Z, Yu J, et al. Role and mechanisms of cytokines in the secondary brain injury after intracerebral hemorrhage. *Prog Neurobiol*. Jul 2019;178:101610. doi:10.1016/j.pneurobio.2019.03.003

54. Ferrarese C, Mascarucci P, Zoia C, et al. Increased cytokine release from peripheral blood cells after acute stroke. *J Cereb Blood Flow Metab*. Sep 1999;19(9):1004-9. doi:10.1097/00004647-199909000-00008
55. Fang Y, Lu J, Wang X, et al. HIF-1alpha Mediates TRAIL-Induced Neuronal Apoptosis via Regulating DcR1 Expression Following Traumatic Brain Injury. *Front Cell Neurosci*. 2020;14:192. doi:10.3389/fncel.2020.00192
56. Cui M, Wang L, Liang X, et al. Blocking TRAIL-DR5 signaling with soluble DR5 reduces delayed neuronal damage after transient global cerebral ischemia. *Neurobiol Dis*. Aug 2010;39(2):138-47. doi:10.1016/j.nbd.2010.03.018
57. TNFSF13B TNF superfamily member 13b NCBI. Accessed 2/2/22, https://www.ncbi.nlm.nih.gov/gene/10673
58. Hedtjarn M, Leverin AL, Eriksson K, Blomgren K, Mallard C, Hagberg H. Interleukin-18 involvement in hypoxic-ischemic brain injury. *J Neurosci*. Jul 15 2002;22(14):5910-9. doi:20026587
59. Yang J, Ding S, Huang W, et al. Interleukin-4 Ameliorates the Functional Recovery of Intracerebral Hemorrhage Through the Alternative Activation of Microglia/Macrophage. *Front Neurosci*. 2016;10:61. doi:10.3389/fnins.2016.00061
60. Xu J, Chen Z, Yu F, et al. IL-4/STAT6 signaling facilitates innate hematoma resolution and neurological recovery after hemorrhagic stroke in mice. *Proc Natl Acad Sci U S A*. Dec 22 2020;117(51):32679-32690. doi:10.1073/pnas.2018497117
61. Garcia JM, Stillings SA, Leclerc JL, et al. Role of Interleukin-10 in Acute Brain Injuries. *Front Neurol*. 2017;8:244. doi:10.3389/fneur.2017.00244
62. Taylor RA, Chang CF, Goods BA, et al. TGF-beta1 modulates microglial phenotype and promotes recovery after intracerebral hemorrhage. *J Clin Invest*. Jan 3 2017;127(1):280-292. doi:10.1172/JCI88647
63. Katsuki H. Exploring neuroprotective drug therapies for intracerebral hemorrhage. *J Pharmacol Sci*. 2010;114(4):366-78. doi:10.1254/jphs.10r05cr
64. Sobrino T, Arias S, Rodriguez-Gonzalez R, et al. High serum levels of growth factors are associated with good outcome in intracerebral hemorrhage. *J Cereb Blood Flow Metab*. Dec 2009;29(12):1968-74. doi:10.1038/jcbfm.2009.182
65. Qian H, Dou Z, Ruan W, He P, Zhang JH, Yan F. ErbB4 Preserves Blood-Brain Barrier Integrity via the YAP/PIK3CB Pathway After Subarachnoid Hemorrhage in Rats. *Front Neurosci*. 2018;12:492. doi:10.3389/fnins.2018.00492
66. Deng W, Luo F, Li BM, Mei L. NRG1-ErbB4 signaling promotes functional recovery in a murine model of traumatic brain injury via regulation of GABA release. *Exp Brain Res*. Dec 2019;237(12):3351-3362. doi:10.1007/s00221-019-05680-2
67. Xu Z, Ford GD, Croslan DR, et al. Neuroprotection by neuregulin-1 following focal stroke is associated with the attenuation of ischemia-induced pro-inflammatory and stress gene expression. *Neurobiol Dis*. Aug 2005;19(3):461-70. doi:10.1016/j.nbd.2005.01.027
68. Xu Z, Croslan DR, Harris AE, Ford GD, Ford BD. Extended therapeutic window and functional recovery after intraarterial administration of neuregulin-1 after focal ischemic stroke. *J Cereb Blood Flow Metab*. Apr 2006;26(4):527-35. doi:10.1038/sj.jcbfm.9600212
69. Li Y, Xu Z, Ford GD, et al. Neuroprotection by neuregulin-1 in a rat model of permanent focal cerebral ischemia. *Brain Res*. Dec 12 2007;1184:277-83. doi:10.1016/j.brainres.2007.09.037
70. Kang M, Yao Y. Oligodendrocytes in intracerebral hemorrhage. *CNS Neurosci Ther*. Oct 2019;25(10):1075-1084. doi:10.1111/cns.13193
71. Kolb B, Saber H, Fadel H, Rajah G. The endocannabinoid system and stroke: A focused review. *Brain Circ*. Jan-Mar 2019;5(1):1-7. doi:10.4103/bc.bc_29_18

72. Wang Z, Li Y, Cai S, Li R, Cao G. Cannabinoid receptor 2 agonist attenuates bloodbrain barrier damage in a rat model of intracerebral hemorrhage by activating the Rac1 pathway. *Int J Mol Med*. Nov 2018;42(5):2914-2922. doi:10.3892/ijmm.2018.3834
73. Schoener B, Borger J. Erythropoietin Stimulating Agents. *StatPearls*. 2022.
74. Grasso G, Graziano F, Sfacteria A, et al. Neuroprotective effect of erythropoietin and darbepoetin alfa after experimental intracerebral hemorrhage. *Neurosurgery*. Oct 2009;65(4):763-9; discussion 769-70. doi:10.1227/01.NEU.0000347475.73347.5F
75. Chu H, Ding H, Tang Y, Dong Q. Erythropoietin protects against hemorrhagic blood-brain barrier disruption through the effects of aquaporin-4. *Lab Invest*. Sep 2014;94(9):1042-53. doi:10.1038/labinvest.2014.84
76. Seyfried DM, Han Y, Yang D, Ding J, Chopp M. Erythropoietin promotes neurological recovery after intracerebral haemorrhage in rats. *Int J Stroke*. Aug 2009;4(4):250-6. doi:10.1111/j.1747-4949.2009.00292.x
77. Yu Z, Tang L, Chen L, Li J, Wu W, Hu C. Erythropoietin reduces brain injury after intracerebral hemorrhagic stroke in rats. *Mol Med Rep*. Nov 2013;8(5):1315-22. doi:10.3892/mmr.2013.1666
78. Wright CB, Dong C, Stark M, et al. Plasma FGF23 and the risk of stroke: the Northern Manhattan Study (NOMAS). *Neurology*. May 13 2014;82(19):1700-6. doi:10.1212/WNL.0000000000000410
79. Huang B, Krafft PR, Ma Q, et al. Fibroblast growth factors preserve blood-brain barrier integrity through RhoA inhibition after intracerebral hemorrhage in mice. *Neurobiol Dis*. Apr 2012;46(1):204-14. doi:10.1016/j.nbd.2012.01.008
80. Zweifel C, Katan M, Schuetz P, et al. Growth hormone and outcome in patients with intracerebral hemorrhage: a pilot study. *Biomarkers*. Sep 2011;16(6):511-6. doi:10.3109/1354750X.2011.599074
81. Serhan A, Boddeke E, Kooijman R. Insulin-Like Growth Factor-1 Is Neuroprotective in Aged Rats With Ischemic Stroke. *Front Aging Neurosci*. 2019;11:349. doi:10.3389/fnagi.2019.00349
82. Nowrangi DS, McBride D, Manaenko A, Dixon B, Tang J, Zhang JH. rhIGF-1 reduces the permeability of the blood-brain barrier following intracerebral hemorrhage in mice. *Exp Neurol*. Feb 2019;312:72-81. doi:10.1016/j.expneurol.2018.11.009
83. Barata JT, Durum SK, Seddon B. Flip the coin: IL-7 and IL-7R in health and disease. *Nat Immunol*. Dec 2019;20(12):1584-1593. doi:10.1038/s41590-019-0479-x
84. An S, Jia Y, Tian Y, et al. Mouse nerve growth factor promotes neurological recovery in patients with acute intracerebral hemorrhage: A proof-of-concept study. *J Neurol Sci*. Nov 15 2020;418:117069. doi:10.1016/j.jns.2020.117069
85. Pei LN, Liu XH, Zhang H, Zhu J, Gao Z, Bi MZ. Clinical efficacy of mouse nerve growth factor plus nimodipine in neonatal intracranial hemorrhage and its effect on plasma PAF, CNP, MMP-2, and neurological function. *Eur Rev Med Pharmacol Sci*. Jan 2021;25(1):215-221. doi:10.26355/eurrev_202101_24387
86. Tajalli-Nezhad S, Karimian M, Beyer C, Atlasi MA, Azami Tameh A. The regulatory role of Toll-like receptors after ischemic stroke: neurosteroids as TLR modulators with the focus on TLR2/4. *Cell Mol Life Sci*. Feb 2019;76(3):523-537. doi:10.1007/s00018-018-2953-2
87. Ashayeri Ahmadabad R, Khaleghi Ghadiri M, Gorji A. The role of Toll-like receptor signaling pathways in cerebrovascular disorders: the impact of spreading depolarization. *J Neuroinflammation*. Apr 7 2020;17(1):108. doi:10.1186/s12974-020-01785-6
88. Madangarli N, Bonsack F, Dasari R, Sukumari-Ramesh S. Intracerebral Hemorrhage: Blood Components and Neurotoxicity. *Brain Sci*. Nov 9 2019;9(11)doi:10.3390/brainsci9110316
89. McGuire VA, Arthur JS. Subverting Toll-Like Receptor Signaling by Bacterial Pathogens. *Front Immunol*. 2015;6:607. doi:10.3389/fimmu.2015.00607

90. Zarubin T, Han J. Activation and signaling of the p38 MAP kinase pathway. *Cell Res*. Jan 2005;15(1):11-8. doi:10.1038/sj.cr.7290257
91. Barone FC, Irving EA, Ray AM, et al. Inhibition of p38 mitogen-activated protein kinase provides neuroprotection in cerebral focal ischemia. *Med Res Rev*. Mar 2001;21(2):129-45. doi:10.1002/1098-1128(200103)21:2<129::aid-med1003>3.0.co;2-h
92. Barone FC, Feuerstein GZ. Inflammatory mediators and stroke: new opportunities for novel therapeutics. *J Cereb Blood Flow Metab*. Aug 1999;19(8):819-34. doi:10.1097/00004647-199908000-00001
93. Guo F, Xu D, Lin Y, et al. Chemokine CCL2 contributes to BBB disruption via the p38 MAPK signaling pathway following acute intracerebral hemorrhage. *FASEB J*. Jan 2020;34(1):1872-1884. doi:10.1096/fj.201902203RR
94. Burton JC, Grimsey NJ. Ubiquitination as a Key Regulator of Endosomal Signaling by GPCRs. *Front Cell Dev Biol*. 2019;7:43. doi:10.3389/fcell.2019.00043
95. Wagner KR. Modeling intracerebral hemorrhage: glutamate, nuclear factor-kappa B signaling and cytokines. *Stroke*. Feb 2007;38(2 Suppl):753-8. doi:10.1161/01.STR.0000255033.02904.db
96. Gupta SC, Sundaram C, Reuter S, Aggarwal BB. Inhibiting NF-kappaB activation by small molecules as a therapeutic strategy. *Biochim Biophys Acta*. Oct-Dec 2010;1799(10-12):775-87. doi:10.1016/j.bbagrm.2010.05.004
97. Su X, Wang H, Zhu L, Zhao J, Pan H, Ji X. Ethyl pyruvate ameliorates intracerebral hemorrhage-induced brain injury through anti-cell death and anti-inflammatory mechanisms. *Neuroscience*. Aug 15 2013;245:99-108. doi:10.1016/j.neuroscience.2013.04.032
98. Lee JU, Kim LK, Choi JM. Revisiting the Concept of Targeting NFAT to Control T Cell Immunity and Autoimmune Diseases. *Front Immunol*. 2018;9:2747. doi:10.3389/fimmu.2018.02747
99. Ting SM, Zhao X, Zheng X, Aronowski J. Excitatory pathway engaging glutamate, calcineurin, and NFAT upregulates IL-4 in ischemic neurons to polarize microglia. *J Cereb Blood Flow Metab*. Mar 2020;40(3):513-527. doi:10.1177/0271678X19838189
100. Sakamoto K, Karelina K, Obrietan K. CREB: a multifaceted regulator of neuronal plasticity and protection. *J Neurochem*. Jan 2011;116(1):1-9. doi:10.1111/j.1471-4159.2010.07080.x
101. Caracciolo L, Marosi M, Mazzitelli J, et al. CREB controls cortical circuit plasticity and functional recovery after stroke. *Nat Commun*. Jun 8 2018;9(1):2250. doi:10.1038/s41467-018-04445-9
102. Lin L, Yihao T, Zhou F, et al. Inflammatory Regulation by Driving Microglial M2 Polarization: Neuroprotective Effects of Cannabinoid Receptor-2 Activation in Intracerebral Hemorrhage. *Front Immunol*. 2017;8:112. doi:10.3389/fimmu.2017.00112
103. Drake CJ. Embryonic and adult vasculogenesis. *Birth Defects Res C Embryo Today*. Feb 2003;69(1):73-82. doi:10.1002/bdrc.10003
104. Arai K, Jin G, Navaratna D, Lo EH. Brain angiogenesis in developmental and pathological processes: neurovascular injury and angiogenic recovery after stroke. *FEBS J*. Sep 2009;276(17):4644-52. doi:10.1111/j.1742-4658.2009.07176.x
105. Tang T, Liu XJ, Zhang ZQ, et al. Cerebral angiogenesis after collagenase-induced intracerebral hemorrhage in rats. *Brain Res*. Oct 17 2007;1175:134-42. doi:10.1016/j.brainres.2007.08.028
106. Greenberg DA. Poststroke angiogenesis, pro: making the desert bloom. *Stroke*. May 2015;46(5):e101-2. doi:10.1161/STROKEAHA.114.007641
107. Adamczak J, Hoehn M. Poststroke angiogenesis, con: dark side of angiogenesis. *Stroke*. May 2015;46(5):e103-4. doi:10.1161/STROKEAHA.114.007642
108. Fagiani E, Christofori G. Angiopoietins in angiogenesis. *Cancer Lett*. Jan 1 2013;328(1):18-26. doi:10.1016/j.canlet.2012.08.018

109. You WK, McDonald DM. The hepatocyte growth factor/c-Met signaling pathway as a therapeutic target to inhibit angiogenesis. *BMB Rep*. Dec 31 2008;41(12):833-9. doi:10.5483/bmbrep.2008.41.12.833
110. Krock BL, Skuli N, Simon MC. Hypoxia-induced angiogenesis: good and evil. *Genes Cancer*. Dec 2011;2(12):1117-33. doi:10.1177/1947601911423654
111. Hashimoto T, Shibasaki F. Hypoxia-inducible factor as an angiogenic master switch. *Front Pediatr*. 2015;3:33. doi:10.3389/fped.2015.00033
112. Zhao X, Guan JL. Focal adhesion kinase and its signaling pathways in cell migration and angiogenesis. *Adv Drug Deliv Rev*. Jul 18 2011;63(8):610-5. doi:10.1016/j.addr.2010.11.001
113. Helker CS, Eberlein J, Wilhelm K, et al. Apelin signaling drives vascular endothelial cells toward a pro-angiogenic state. *Elife*. Sep 21 2020;9doi:10.7554/eLife.55589
114. Zhang J, Zhang H, Chen Y, et al. Plateletderived growth factor D promotes the angiogenic capacity of endothelial progenitor cells. *Mol Med Rep*. Jan 2019;19(1):125-132. doi:10.3892/mmr.2018.9692
115. Andreone BJ, Larhammar M, Lewcock JW. Cell Death and Neurodegeneration. *Cold Spring Harb Perspect Biol*. Feb 3 2020;12(2)doi:10.1101/cshperspect.a036434
116. Bobinger T, Burkardt P, H BH, Manaenko A. Programmed Cell Death after Intracerebral Hemorrhage. *Curr Neuropharmacol*. 2018;16(9):1267-1281. doi:10.2174/1570159X15666170602112851
117. Fang Y, Gao S, Wang X, et al. Programmed Cell Deaths and Potential Crosstalk With Blood-Brain Barrier Dysfunction After Hemorrhagic Stroke. *Front Cell Neurosci*. 2020;14:68. doi:10.3389/fncel.2020.00068
118. Xi G, Strahle J, Hua Y, Keep RF. Progress in translational research on intracerebral hemorrhage: is there an end in sight? *Prog Neurobiol*. Apr 2014;115:45-63. doi:10.1016/j.pneurobio.2013.09.007
119. Fan TJ, Han LH, Cong RS, Liang J. Caspase family proteases and apoptosis. *Acta Biochim Biophys Sin (Shanghai)*. Nov 2005;37(11):719-27. doi:10.1111/j.1745-7270.2005.00108.x
120. Marsden VS, O'Connor L, O'Reilly LA, et al. Apoptosis initiated by Bcl-2-regulated caspase activation independently of the cytochrome c/Apaf-1/caspase-9 apoptosome. *Nature*. Oct 10 2002;419(6907):634-7. doi:10.1038/nature01101
121. Koenig MN, Naik E, Rohrbeck L, et al. Pro-apoptotic BIM is an essential initiator of physiological endothelial cell death independent of regulation by FOXO3. *Cell Death Differ*. Nov 2014;21(11):1687-95. doi:10.1038/cdd.2014.90
122. Kataoka T, Holler N, Micheau O, et al. Bcl-rambo, a novel Bcl-2 homologue that induces apoptosis via its unique C-terminal extension. *J Biol Chem*. Jun 1 2001;276(22):19548-54. doi:10.1074/jbc.M010520200
123. Opferman JT, Kothari A. Anti-apoptotic BCL-2 family members in development. *Cell Death Differ*. Jan 2018;25(1):37-45. doi:10.1038/cdd.2017.170
124. Loo LSW, Soetedjo AAP, Lau HH, et al. BCL-xL/BCL2L1 is a critical anti-apoptotic protein that promotes the survival of differentiating pancreatic cells from human pluripotent stem cells. *Cell Death Dis*. May 18 2020;11(5):378. doi:10.1038/s41419-020-2589-7
125. Vogler M. BCL2A1: the underdog in the BCL2 family. *Cell Death Differ*. Jan 2012;19(1):67-74. doi:10.1038/cdd.2011.158
126. Chiarini A, Liu D, Armato U, Dal Pra I. Bcl10 crucially nucleates the pro-apoptotic complexes comprising PDK1, PKCzeta and caspase-3 at the nuclear envelope of etoposide-treated human cervical carcinoma C4-I cells. *Int J Mol Med*. Sep 2015;36(3):845-56. doi:10.3892/ijmm.2015.2290
127. Gehring T, Seeholzer T, Krappmann D. BCL10 - Bridging CARDs to Immune Activation. *Front Immunol*. 2018;9:1539. doi:10.3389/fimmu.2018.01539

128. Ruland J, Duncan GS, Elia A, et al. Bcl10 is a positive regulator of antigen receptor-induced activation of NF-kappaB and neural tube closure. *Cell*. Jan 12 2001;104(1):33-42. doi:10.1016/s0092-8674(01)00189-1
129. Kurosu T, Fukuda T, Miki T, Miura O. BCL6 overexpression prevents increase in reactive oxygen species and inhibits apoptosis induced by chemotherapeutic reagents in B-cell lymphoma cells. *Oncogene*. Jul 17 2003;22(29):4459-68. doi:10.1038/sj.onc.1206755
130. Kumagai T, Miki T, Kikuchi M, et al. The proto-oncogene Bc16 inhibits apoptotic cell death in differentiation-induced mouse myogenic cells. *Oncogene*. Jan 14 1999;18(2):467-75. doi:10.1038/sj.onc.1202306
131. Geiss-Friedlander R, Melchior F. Concepts in sumoylation: a decade on. *Nat Rev Mol Cell Biol*. Dec 2007;8(12):947-56. doi:10.1038/nrm2293
132. Karhausen J, Ulloa L, Yang W. SUMOylation Connects Cell Stress Responses and Inflammatory Control: Lessons From the Gut as a Model Organ. *Front Immunol*. 2021;12:646633. doi:10.3389/fimmu.2021.646633
133. Adorisio S, Fierabracci A, Muscari I, et al. SUMO proteins: Guardians of immune system. *J Autoimmun*. Nov 2017;84:21-28. doi:10.1016/j.jaut.2017.09.001
134. Stankovic-Valentin N, Melchior F. Control of SUMO and Ubiquitin by ROS: Signaling and disease implications. *Mol Aspects Med*. Oct 2018;63:3-17. doi:10.1016/j.mam.2018.07.002
135. Yang W, Sheng H, Wang H. Targeting the SUMO pathway for neuroprotection in brain ischaemia. *Stroke Vasc Neurol*. Sep 2016;1(3):101-107. doi:10.1136/svn-2016-000031
136. Johnson ES. Protein modification by SUMO. *Annu Rev Biochem*. 2004;73:355-82. doi:10.1146/annurev.biochem.73.011303.074118
137. Guo Y, Wang Y, Ma Y, Chen G, Yue P, Li Y. Upregulation of lncRNA SUMO1P3 promotes proliferation, invasion and drug resistance in gastric cancer through interacting with the CNBP protein. 10.1039/C9RA09497K. *RSC Advances*. 2020;10(10):6006-6016. doi:10.1039/C9RA09497K
138. Tian C, Jin Y, Shi S. Long non-coding RNA SUMO1P3 may promote cell proliferation, migration, and invasion of pancreatic cancer via EMT signaling pathway. *Oncol Lett*. Nov 2018;16(5):6109-6115. doi:10.3892/ol.2018.9378
139. Deng D, Mo Y, Xue L, Shao N, Cao J. Long non-coding RNA SUMO1P3 promotes tumour progression by regulating cell proliferation and invasion in glioma. *Exp Ther Med*. May 2021;21(5):491. doi:10.3892/etm.2021.9922
140. NEDD4L NEDD4 like E3 ubiquitin protein ligase. NCBI. Accessed 2/1/22, https://www.ncbi.nlm.nih.gov/gene/23327
141. Kim T, Chokkalla AK, Vemuganti R. Deletion of ubiquitin ligase Nedd4l exacerbates ischemic brain damage. *J Cereb Blood Flow Metab*. May 2021;41(5):1058-1066. doi:10.1177/0271678X20943804
142. Scotti MM, Swanson MS. RNA mis-splicing in disease. *Nat Rev Genet*. Jan 2016;17(1):19-32. doi:10.1038/nrg.2015.3
143. Dykstra-Aiello C, Jickling GC, Ander BP, et al. Intracerebral Hemorrhage and Ischemic Stroke of Different Etiologies Have Distinct Alternatively Spliced mRNA Profiles in the Blood: a Pilot RNA-seq Study. *Transl Stroke Res*. Aug 2015;6(4):284-9. doi:10.1007/s12975-015-0407-9
144. Bourgeois CF, Mortreux F, Auboeuf D. The multiple functions of RNA helicases as drivers and regulators of gene expression. *Nat Rev Mol Cell Biol*. Jul 2016;17(7):426-38. doi:10.1038/nrm.2016.50
145. Yang P, Mathieu C, Kolaitis RM, et al. G3BP1 Is a Tunable Switch that Triggers Phase Separation to Assemble Stress Granules. *Cell*. Apr 16 2020;181(2):325-345 e28. doi:10.1016/j.cell.2020.03.046
146. Dardenne E, Polay Espinoza M, Fattet L, et al. RNA helicases DDX5 and DDX17 dynamically orchestrate transcription, miRNA, and splicing programs in cell differentiation. *Cell Rep*. Jun 26 2014;7(6):1900-13. doi:10.1016/j.celrep.2014.05.010

147. Lee T, Pelletier J. The biology of DHX9 and its potential as a therapeutic target. *Oncotarget*. Jul 5 2016;7(27):42716-42739. doi:10.18632/oncotarget.8446
148. Chen MC, Tippana R, Demeshkina NA, et al. Structural basis of G-quadruplex unfolding by the DEAH/RHA helicase DHX36. *Nature*. Jun 2018;558(7710):465-469. doi:10.1038/s41586-018-0209-9
149. Huelga SC, Vu AQ, Arnold JD, et al. Integrative genome-wide analysis reveals cooperative regulation of alternative splicing by hnRNP proteins. *Cell Rep*. Feb 23 2012;1(2):167-78. doi:10.1016/j.celrep.2012.02.001
150. Martinez-Contreras R, Cloutier P, Shkreta L, Fisette JF, Revil T, Chabot B. hnRNP proteins and splicing control. *Adv Exp Med Biol*. 2007;623:123-47. doi:10.1007/978-0-387-77374-2_8
151. Uren PJ, Bahrami-Samani E, de Araujo PR, et al. High-throughput analyses of hnRNP H1 dissects its multi-functional aspect. *RNA Biol*. 2016;13(4):400-11. doi:10.1080/15476286.2015.1138030
152. Han SP, Tang YH, Smith R. Functional diversity of the hnRNPs: past, present and perspectives. *Biochem J*. Sep 15 2010;430(3):379-92. doi:10.1042/BJ20100396
153. Geuens T, Bouhy D, Timmerman V. The hnRNP family: insights into their role in health and disease. *Hum Genet*. Aug 2016;135(8):851-67. doi:10.1007/s00439-016-1683-5
154. Donde A, Sun M, Ling JP, et al. Splicing repression is a major function of TDP-43 in motor neurons. *Acta Neuropathol*. Nov 2019;138(5):813-826. doi:10.1007/s00401-019-02042-8
155. Torres P, Ramirez-Nunez O, Romero-Guevara R, et al. Cryptic exon splicing function of TARDBP interacts with autophagy in nervous tissue. *Autophagy*. 2018;14(8):1398-1403. doi:10.1080/15548627.2018.1474311
156. McDonald KK, Aulas A, Destroismaisons L, et al. TAR DNA-binding protein 43 (TDP-43) regulates stress granule dynamics via differential regulation of G3BP and TIA-1. *Hum Mol Genet*. Apr 1 2011;20(7):1400-10. doi:10.1093/hmg/ddr021
157. Pink RC, Wicks K, Caley DP, Punch EK, Jacobs L, Carter DR. Pseudogenes: pseudo-functional or key regulators in health and disease? *RNA*. May 2011;17(5):792-8. doi:10.1261/rna.2658311
158. Klein SL, Flanagan KL. Sex differences in immune responses. *Nat Rev Immunol*. Oct 2016;16(10):626-38. doi:10.1038/nri.2016.90
159. Marini S, Morotti A, Ayres AM, et al. Sex differences in intracerebral hemorrhage expansion and mortality. *J Neurol Sci*. Aug 15 2017;379:112-116. doi:10.1016/j.jns.2017.05.057
160. Marini S, Morotti A, Lena UK, et al. Men Experience Higher Risk of Pneumonia and Death After Intracerebral Hemorrhage. *Neurocrit Care*. Feb 2018;28(1):77-82. doi:10.1007/s12028-017-0431-6
161. Jansen R, Batista S, Brooks AI, et al. Sex differences in the human peripheral blood transcriptome. *BMC Genomics*. Jan 17 2014;15:33. doi:10.1186/1471-2164-15-33
162. Tian Y, Stamova B, Jickling GC, et al. Effects of gender on gene expression in the blood of ischemic stroke patients. *J Cereb Blood Flow Metab*. May 2012;32(5):780-91. doi:10.1038/jcbfm.2011.179
163. Stamova B, Jickling GC, Ander BP, et al. Gene expression in peripheral immune cells following cardioembolic stroke is sexually dimorphic. *PLoS One*. 2014;9(7):e102550. doi:10.1371/journal.pone.0102550
164. Stamova B, Tian Y, Jickling G, et al. The X-chromosome has a different pattern of gene expression in women compared with men with ischemic stroke. *Stroke*. Feb 2012;43(2):326-34. doi:10.1161/STROKEAHA.111.629337
165. Dykstra-Aiello C, Jickling GC, Ander BP, et al. Altered Expression of Long Noncoding RNAs in Blood After Ischemic Stroke and Proximity to Putative Stroke Risk Loci. *Stroke*. Dec 2016;47(12):2896-2903. doi:10.1161/STROKEAHA.116.013869

166. Dykstra-Aiello C, Sharp FR, Jickling GC, et al. Alternative Splicing of Putative Stroke/Vascular Risk Factor Genes Expressed in Blood Following Ischemic Stroke Is Sexually Dimorphic and Cause-Specific. *Front Neurol*. 2020;11:584695. doi:10.3389/fneur.2020.584695
167. Zhou F, Wang YK, Zhang CG, Wu BY. miR-19a/b-3p promotes inflammation during cerebral ischemia/reperfusion injury via SIRT1/FoxO3/SPHK1 pathway. *J Neuroinflammation*. May 29 2021;18(1):122. doi:10.1186/s12974-021-02172-5
168. Chai Z, Gong J, Zheng P, Zheng J. Inhibition of miR-19a-3p decreases cerebral ischemia/reperfusion injury by targeting IGFBP3 in vivo and in vitro. *Biol Res*. Apr 20 2020;53(1):17. doi:10.1186/s40659-020-00280-9
169. Ge XL, Wang JL, Liu X, Zhang J, Liu C, Guo L. Inhibition of miR-19a protects neurons against ischemic stroke through modulating glucose metabolism and neuronal apoptosis. *Cell Mol Biol Lett*. 2019;24:37. doi:10.1186/s11658-019-0160-2
170. Welten SM, Bastiaansen AJ, de Jong RC, et al. Inhibition of 14q32 MicroRNAs miR-329, miR-487b, miR-494, and miR-495 increases neovascularization and blood flow recovery after ischemia. *Circ Res*. Sep 26 2014;115(8):696-708. doi:10.1161/CIRCRESAHA.114.304747
171. Davenport AP, Hyndman KA, Dhaun N, et al. Endothelin. *Pharmacol Rev*. Apr 2016;68(2):357-418. doi:10.1124/pr.115.011833
172. CD48. The Human Protein Atlas. Accessed 2/2/22, https://www.proteinatlas.org/ENSG00000117091-CD48
173. Kooistra J, Milojevic J, Melacini G, Ortega J. A new function of human HtrA2 as an amyloid-beta oligomerization inhibitor. *J Alzheimers Dis*. 2009;17(2):281-94. doi:10.3233/JAD-2009-1037
174. Novikova G, Kapoor M, Tcw J, et al. Integration of Alzheimer's disease genetics and myeloid genomics identifies disease risk regulatory elements and genes. *Nat Commun*. Mar 12 2021;12(1):1610. doi:10.1038/s41467-021-21823-y
175. Ullrich S, Munch A, Neumann S, Kremmer E, Tatzelt J, Lichtenthaler SF. The novel membrane protein TMEM59 modulates complex glycosylation, cell surface expression, and secretion of the amyloid precursor protein. *J Biol Chem*. Jul 2 2010;285(27):20664-74. doi:10.1074/jbc.M109.055608
176. Turner MD, Nedjai B, Hurst T, Pennington DJ. Cytokines and chemokines: At the crossroads of cell signalling and inflammatory disease. *Biochim Biophys Acta*. Nov 2014;1843(11):2563-2582. doi:10.1016/j.bbamcr.2014.05.014
